# Effectiveness of non-pharmaceutical public health interventions against COVID-19: A systematic review and meta-analysis

**DOI:** 10.1371/journal.pone.0260371

**Published:** 2021-11-23

**Authors:** Shabnam Iezadi, Kamal Gholipour, Saber Azami-Aghdash, Akbar Ghiasi, Aziz Rezapour, Hamid Pourasghari, Fariba Pashazadeh

**Affiliations:** 1 Hospital Management Research Center, Health Management Research Institute, Iran University of Medical Science, Tehran, Iran; 2 Social Determinants of Health Research Center, School of Management and Medical Informatics, Tabriz University of Medical Sciences, Tabriz, Iran; 3 Tabriz Health Service Management Research Center, Tabriz University of Medical Sciences, Tabriz, Iran; 4 HEB School of Business & Administration, University of the Incarnate Word, San Antonio, Texas, United States of America; 5 Health Management and Economics Research Center, Health Management Research Institute, Iran University of Medical Sciences, Tehran, Iran; 6 Research Center of Evidence-Based Medicine (EBM), Tabriz University of Medical Sciences, Tabriz, Iran; Post Graduate Institute of Medical Education and Research, INDIA

## Abstract

Non-Pharmaceutical Public Health Interventions (NPHIs) have been used by different countries to control the spread of the COVID-19. Despite available evidence regarding the effectiveness of NPHSs, there is still no consensus about how policymakers can trust these results. Studies on the effectiveness of NPHSs are single studies conducted in specific communities. Therefore, they cannot individually prove if these interventions have been effective in reducing the spread of the infection and its adverse health outcomes. In this systematic review, we aimed to examine the effects of NPHIs on the COVID-19 case growth rate, death growth rate, Intensive Care Unit (ICU) admission, and reproduction number in countries, where NPHIs have been implemented. We searched relevant electronic databases, including Medline (via PubMed), Scopus, CINAHL, Web of Science, etc. from late December 2019 to February 1, 2021. The key terms were primarily drawn from Medical Subject Heading (MeSh and Emtree), literature review, and opinions of experts. Peer-reviewed quasi-experimental studies were included in the review. The PROSPERO registration number is CRD42020186855. Interventions were NPHIs categorized as lockdown, stay-at-home orders, social distancing, and other interventions (mask-wearing, contact tracing, and school closure). We used PRISMA 2020 guidance for abstracting the data and used Cochrane Effective Practice and Organization of Practice (EPOC) Risk of Bias Tool for quality appraisal of the studies. Hartung-Knapp-Sidik-Jonkman random-effects model was performed. Main outcomes included COVID-19 case growth rate (percentage daily changes), COVID-19 mortality growth rate (percentage daily changes), COVID-19 ICU admission (percentage daily changes), and COVID-19 reproduction number changes. Our search strategies in major databases yielded 12,523 results, which decreased to 7,540 articles after eliminating duplicates. Finally, 35 articles qualified to be included in the systematic review among which 23 studies were included in the meta-analysis. Although studies were from both low-income and high-income countries, the majority of them were from the United States (13 studies) and China (five studies). Results of the meta-analysis showed that adoption of NPHIs has resulted in a 4.68% (95% CI, -6.94 to -2.78) decrease in daily case growth rates, 4.8% (95 CI, -8.34 to -1.40) decrease in daily death growth rates, 1.90 (95% CI, -2.23 to -1.58) decrease in the COVID-19 reproduction number, and 16.5% (95% CI, -19.68 to -13.32) decrease in COVID-19 daily ICU admission. A few studies showed that, early enforcement of lockdown, when the incidence rate is not high, contributed to a shorter duration of lockdown and a lower increase of the case growth rate in the post-lockdown era. The majority of NPHIs had positive effects on restraining the COVID-19 spread. With the problems that remain regarding universal access to vaccines and their effectiveness and considering the drastic impact of the nationwide lockdown and other harsh restrictions on the economy and people’s life, such interventions should be mitigated by adopting other NPHIs such as mass mask-wearing, patient/suspected case isolation strategies, and contact tracing. Studies need to address the impact of NPHIs on the population’s other health problems than COVID-19.

## Introduction

It has been more than a year since the outbreak of Coronavirus infection (COVID-19), which has become a major global health threat. For about 12 months after the onset of the pandemic, there was no reliable vaccine or treatment to control or treat the disease. Currently, although there are some effective vaccines, not all countries across the globe have access to the vaccines. As a result, it is still important to slow down the spread of the infection by Non-Pharmaceutical Public Health Interventions (NPHIs) [1]. Various NPHIs have been used by different countries to control the spread of the disease, including patient isolation, contact tracing, lockdown, quarantine, travel ban, social distancing, school closing and mass gathering containment. It is widely agreed that NPHIs could reduce the transmission of infection until global immunization is achieved [2].

COVID-19 pandemic has had serious consequences worldwide both directly by affecting the health of individuals and indirectly as a result of implementing NPHIs by governments. For instance, a study showed that cancer diagnoses decreased by 39% in 2020 compared to previous years [3]. Another study in the UK concluded that delayed diagnosis of cancer due to COVID-19 may increase the number of preventable cancer deaths [4]. The other study in a province of Pakistan showed that one out of every two children in the urban areas and a higher rate of children in rural areas have missed regular vaccinations for other infectious diseases during the lockdown [5].

Similar to many crises [6], COVID-19 also has worsened inequities in different societies, causing dramatically higher unfavourable health outcomes and economic losses among deprived countries due to higher vulnerabilities to both the disease and also nature of the public health measures [7,8]. Literature shows that the racial minorities in many countries are at higher risk of infection [9,10], and NPHIs may have a greater effect on reducing the spread of the infection in white populations [9]. A policy brief by International Growth Centre reported that a higher portion of the population in sub-Saharan Africa has suffered massive poverty as a result of lockdown [11]. Socioeconomically deprived populations are particularly vulnerable to the NPHIs targeting the COVID-19 because of their low access to electronic communication tools, more stressful lifestyle, comorbidities due to weaker immune system, and types of occupation such as post office, restaurant workers, cashiers, etc. [12].

Despite some negative impacts of NPHIs, particularly lockdown [13], adoption of these measures have been vital to control the spread of COVID-19. Previous literature has shown the effectiveness of NPHIs such as hand hygiene, wearing facemasks, and contact-reducing strategies in controlling the spread of different infectious influenza-like diseases [14,15]. There is also evidence showing the effectiveness of NPHIs in controlling COVID-19 in both high- and low-income countries [16,17].

Despite available evidence regarding the effectiveness of NPHs, there is still no consensus about how policymakers can trust these results. On the one hand, devastating consequences of NPHIs and on the other hand, the success of the NPHIs in combating and preventing COVID-19-related hospitalization and death. There are some important questions like have there been any significant decrease in the spread of COVID-19 and its adverse health outcomes due to the implementation of NPHIs? Have benefits offset negative effects?

Researchers have used different types of studies or different measures to investigate the impact of NPHIs. As a result, it has become challenging to evaluate the effectiveness of different interventions. For example, several mathematical modelling and ecological studies using secondary data indicated that social distancing and lockdown have been effective measures in reducing the number of COVID-19 deaths [14,18,19]. A study demonstrated the effectiveness of social distancing, spring semester postponing, and contact tracing [20]. Another study has focused on different aspect of the measures and concluded that the NPHIs in Italy were not timely and effective enough [17]. All these studies are single studies conducted in specific communities. Therefore, they cannot individually prove if these interventions have been effective in reducing the incidence, mortality, morbidity, and other negative health outcomes of the disease. Additionally, it is difficult for policymakers to use a large number of studies to guide their further actions in controlling the pandemic. Thus, it seems that conducting a systematic review of literature and meta-analysis regarding the effectiveness of different NPHIs could be beneficial for researchers and policymakers. In this systematic review and meta-analysis, we aimed to examine the effects of NPHIs on the COVID-19 case growth rate, Intensive Care Unit (ICU) admission, mortality growth rate, and reproduction number (R) in countries, where NPHIs have been implemented.

## Methods

This systematic review and meta-analysis is the first phase of a broader review, where the protocol was already published [21] and the PROSPERO registration number is CRD42020186855. We used the PRISMA 2020 statement: an updated guideline for reporting systematic reviews to design the study and outline the final report [22].

### Inclusion and exclusion criteria

#### Population

All countries implemented any of NPHIs to control the COVID-19 in their communities.

#### Intervention

Interventions were the NPHIs using suppression strategies, mitigation strategies, or both strategies simultaneously, at the community level categorized as lockdown, stay-at-home, social distancing, and other interventions (mask-wearing, contact tracing, and school closure). The definition of the interventions is summarized in S1 Table.

#### Comparator

We consider two types of comparison including, comparison of the outcomes before and after the adoption of NPHIs in a community/country, and, comparison of the outcomes between communities that have adopted NPHIs with those that have not adopted NPHIs.

#### Outcome

Main outcomes included COVID-19 case growth rate (percentage daily changes), COVID-19 mortality growth rate (percentage daily changes), COVID-19 ICU admission (percentage daily changes), and reproduction number changes.

#### Study design

Peer-reviewed, quasi-experimental studies on the effects of NPHIs on selected outcomes including retrospective and prospective cohort studies, cross-sectional studies, and interrupted times-series studies were included in the review.

#### Exclusion criteria

Studies were excluded from our literature review based on the following criteria:

▪ Mathematical modelling studies that have used simulations, predictions, and scenarios to examine the effects.
▪ Studies targeting only the special group of populations rather than the community, for example, patients with cancer, pregnant women, etc.
▪ Studies that did not provide complete information to assess the effects were excluded from the meta-analysis while were reported in the descriptive analysis.

### Search strategy

We classified key terms in three main domains, including NPHIs, COVID-19, and effectiveness. These terms were primarily drawn from Medical Subject Heading (MeSh and Emtree), initial literature review, and opinions of experts on the topic and were finalized through a pilot search. The search strategy was designed by the contribution of a well-experienced medical librarian (FP) and four researchers in the field of health services research and public health (SI, KG, SA, and AG). Full search strategies are available in the S2 Table. We also contacted the corresponding authors of the studies with incomplete information on the effect size, nevertheless, we did not receive any additional information.

### Information sources

We searched relevant electronic databases, including Medline (via PubMed), Cochrane Library, Scopus, CINAHL, ProQuest, Embase, and Web of Science, (from late December 2019 to February 1, 2021). We also carefully reviewed references of articles found and citation lists of the relevant studies, gray literature (Gray.net), preprint databases, the website of WHO, and other relevant sources of evidence.

### Screening and data extraction

After gathering all search results and entering them into EndNoteX8 software and removing the duplicate results, three investigators separately (AR, HP, AG) reviewed the title/abstract of the results using eligibility criteria. Two investigators (SI, KG) carefully reviewed the full texts of the relevant studies in an iterative way to determine the eligible studies. To avoid deviating from the study objectives, the investigators used eligibility criteria and had the research question in mind through the whole process of title/abstract screening and reviewing the full texts. Any disagreements in every stage were solved by discussing with the rest of the team. Three investigators (SI, KG, and SA) extracted the data using a data extraction table, which was prepared in advance and was modified and finalized after a pilot testing on a sample of three papers. The full data extraction sheet completed in detail is available in S3 Table.

### Critical appraisal of individual sources of evidence

Two investigators (SI and KG) independently reviewed the methodology of the eligible papers to assure the internal validity of the included studies. Since all the included papers were quasi-experimental studies that have assessed the effects of NPHIs, we used the Cochrane Effective Practice and Organization of Practice (EPOC) Risk of Bias Tool. Using the EPOC risk of bias tool for interrupted time-series studies, the investigators scored the studies as “low-risk”, “high-risk”, and “unclear-risk” for each of seven standard criteria. Moreover, the investigators used the EPOC risk of bias tool for studies with a separate control group scoring the studies as “low-risk”, “high-risk”, and “unclear-risk” for each of nine specified standard criteria (Table in S4 and S5 Tables) [23].

### Data analysis

#### Descriptive synthesis

Based on the information extracted from the studies, we provided a brief and narrative explanation of the outcome measures as primary findings of the review.

#### Quantitative synthesis

We used data points from every observational study to perform random-effects meta-analysis using Stata (StataCorp, version 16). We used daily percentage change and 95% confidence interval performing the Hartung-Knapp-Sidik-Jonkman random-effects model. We conducted summary estimates to examine the heterogeneity as a prerequisite to meta-analysis. We used forest plots to visually present the extent of heterogeneity among studies. We computed variance between studies by I2 statistics to examine statistical heterogeneity (I2 ranges between 0 and 100% with values of 0–25% demonstrate low heterogeneity, and 75–100% illustrate considerable heterogeneity). We used a random-effect model for analysis, where I2 was greater than 50%. We have reported the results of the Tablau2 and Cochran Q test to show heterogeneity, and a P-value of < 0.05 is considered a statistically significant level. We used the Funnel plot and Egger’s regression test to evaluate the possibility of publication bias and a P-value of < 0.1 was considered a statistically significant level. We also used the Trim-and-Fill test with the linear estimator method in case of any publication bias. We conducted subgroup analysis based on each group of interventions.

Some studies reported outcome measures based on weakly changes. Using the compound growth rate formula, we converted those measures to a daily basis. DGR = (1+WGR)^1/7^–1; where DGR is the daily growth rate and WGR is the weekly growth rate.

## Results

Our search strategies in major databases yielded 12,523 results, which decreased to 7,361 articles after eliminating duplicates. We excluded 74 papers from the systematic review due to using modelling studies/simulation/scenario (n = 56), exploring the irrelevant outcomes (n = 2), theoretical paper (n = 2), a specific small population (n = 1), irrelevant comparison (n = 2), multicounty studies in which there were no country-level data or several countries were considered as a single unit of observation (n = 3), and not being peer-reviewed (n = 8). Additionally, 12 studies were not included in the meta-analysis due to inadequate data for estimating the effect size [24–35]. Through the precise and vigorous review of the full texts of the relevant studies, we found that 35 articles qualified to be included in the systematic review based on the previously mentioned eligibility criteria [24–58].

**Fig 1:**
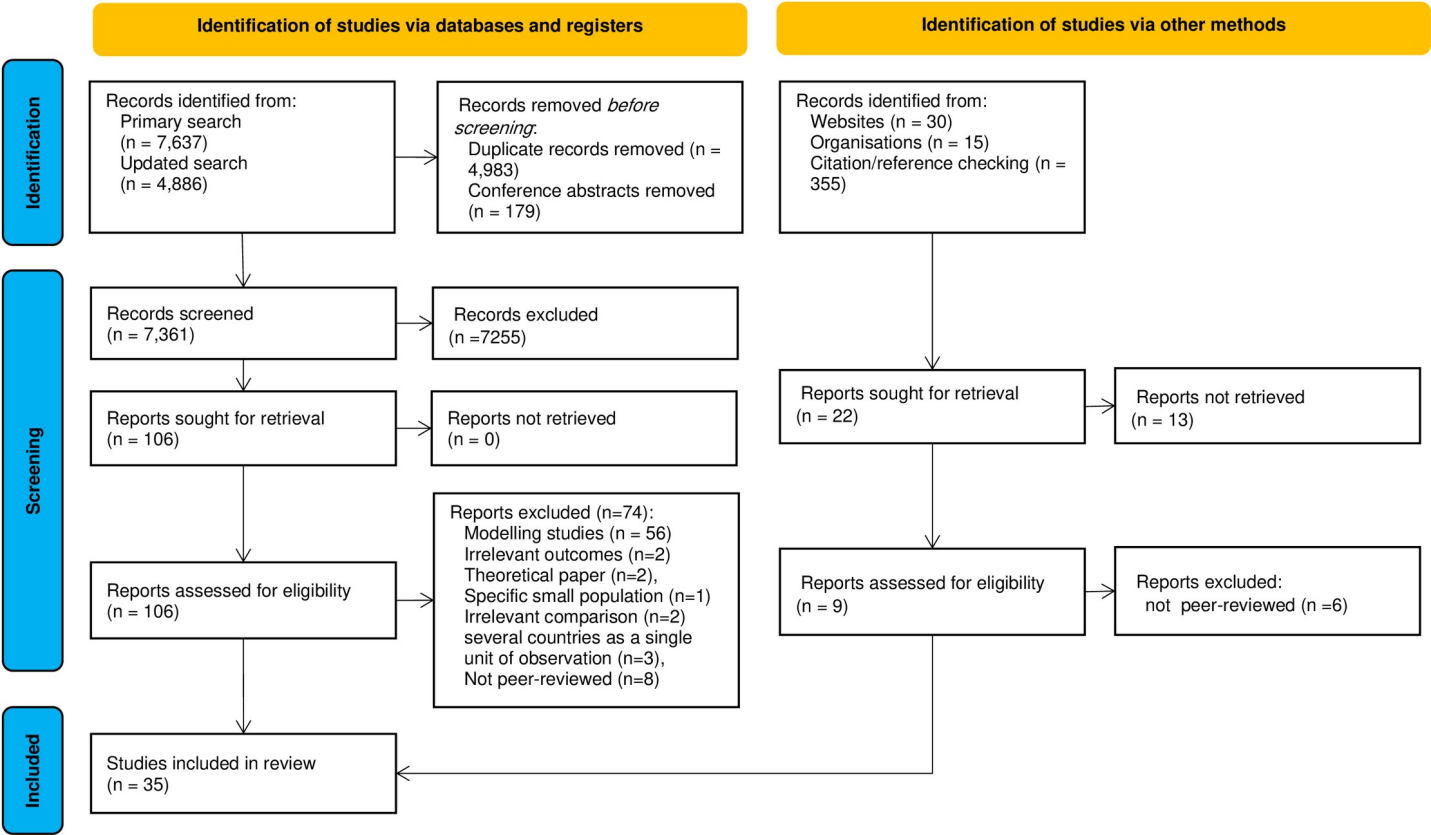
PRISMA flow diagram for systematic reviews.

The PRISMA diagram showing the detailed information on search results and selection process is presented in Fig 1.

Although studies were from both low-income and high-income countries, the majority of them were from the United States (US) (13 studies) and China (five studies). Studies have targeted a wide range of NPHIs in the context of COVID-19 individually, including school closure (two studies), shelter-in-place order (two studies), social distancing (eight studies), lockdown (11 studies), stay-at-home rule (six studies), quarantine (four studies), mass screening (one study), universal symptom survey (one study), and face-masking recommendation (one study), and in combination with each other (five studies).

Almost all studies were observational quasi-experimental studies, the majority of them were time-series analyses and just two studies were comparisons between control and intervention groups [30,58]. Therefore, most studies did score high-risk for a few items including “item 1: intervention independent of other changes” and “item 3: intervention unlikely to affect data collection” through the quality appraisal of the included studies. The results of the quality appraisal are presented in the S4 and S5 Tables.

Concerning the impacts of lockdown, the majority of the studies showed reductions in different COVID-19 indicators during and in the final stages of the lockdown or immediately after a lockdown was lifted. For instance, one study in Spain showed a maximum reduction of 8.85% for incidence rate, 10.20% for hospital admission rates, 20.71% for ICU admission rates, and 19.00% for death rates. The minimum level of reductions were 1.42%, 2.62%, 3.74%, 1.88%, and 2.90% for daily incidence rates, daily hospital admission rates, daily ICU admission rates, daily death rates, and daily recovered rates, retrospectively [33]. Another study in Spain and Italy showed that the daily growth rate of the new cases, deaths, and ICU admissions were approximately decreased by 70%, 69%, and 63% in Spain and by 42%, 58%, and 77% in Italy, respectively. This study also illustrated that a more restrictive lockdown in the next step decreased the rate of the new cases, deaths, and ICU admissions by 2.7, 1.8, and 5.6 percentages in Spain, and 2.0, 0.2, and 16.8 percentages in Italy, retrospectively [51]. In India, two studies showed slight effects of lockdown on decreasing case growth rate [45,47] and three studies showed the decrease of reproduction rate before and after lockdown including (all numbers in the parentheses show 95% confidence intervals) 5.72 (4.34, 7.37) to 1.37 (1.25, 1.5); 2.55 (2.11, 3.05) to 2.41 (1.99, 2.88); 1.72 (1.38, 2.11) to 2.05 (1.91, 2.18) [45]; 2.51 (2.06, 3.14) to 1.83 (1.71, 1.93) [47]; and, 3.36 (3.03, 3.71) to 1.27 (1.26, 1.28) [54]. One study in Brazil showed a range of 87% to 93% decreases in daily incidence trend and 87% to 94% decreases in daily death trend as the results of lockdown enforcements [50]. The results of a study on 27 countries showed that 15 days after the lockdown, trends daily cases of COVID-19 decreased, however, no significant decrease was observed in the mortality growth rate [28].

Regarding the social distancing measures, a study in the US showed that implementation of four social distancing policies, including shelter-in-place order, restaurant/gym/entertainment closures, no large events, and school closure, decreased the growth rate by 12.0% after twenty-one days or more. According to this study, the case growth rate decreased by 8.6% points (P<0.005) as a result of shelter-in-place order and by 5.2% points as a result of restaurant/gym/entertainment closures, after twenty-one days onward. However, no large events and school closure did not reveal a statistically significant effect on case growth rate when they were examined individually [40]. A few studies showed reductions in the case growth rate in the US including a 0.9% decrease as a result of social distancing measures [49], 3.1% as a result of statewide restrictions on internal movement [49], and 90% reduction as a result of face-masking recommendation [52]. In China, the greatest decrease of reproductive number, from 3.61 [3.49, 3.73] to 1.37 (95% CI,1.34, 1.40), was attributed to cordons sanitaire, traffic restriction, and home quarantine [31]. In contrast, a study in Portugal did not report a notable effect of contact tracing and quarantine of close contacts on reducing the number of secondary cases of COVID-19 [58].

**Fig 2:**
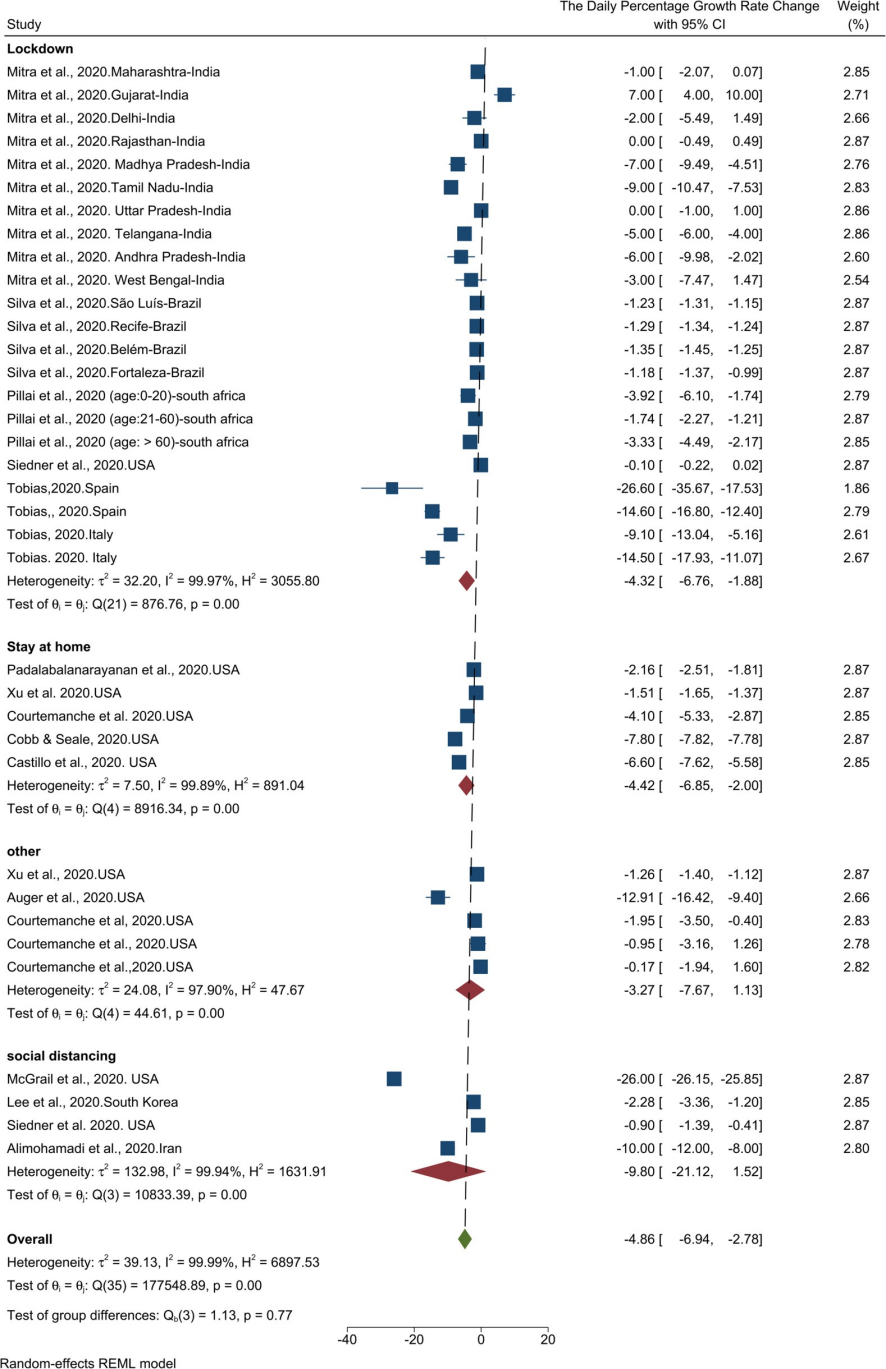
Results of the meta-analysis of the effects of NPHIs on the percentage changes of the COVID-19 daily case growth rate in communities based on a random effect model.

Meta-analysis of the results of the 14 studies showed that adoption of NPHIs has resulted in a 4.86% (95% CI, -6.94 to -2.78) decrease in daily case growth rate in the communities (Table 1). The results of the heterogeneity analysis showed high heterogeneity among studies. The results of the publication bias assessment indicated that there is a high probability of publication bias (Fig in S1 Fig). The results of the Trim-and- Fill test also show that seven cases were probably missed so that by imputing those studies and their effect, the size of the change would decrease from -4.86 to -6.23 (95% CI, -8.23 to -4.30). Fig 2 illustrates the results of the effect of NPHIs on the COVID-19 daily case growth rate.

**Table 1:** Results of the meta-analysis, heterogeneity analysis, and publication bias assessment.

Variable	Type of intervention	Number of studies (cases)	Summary estimates (change) [95% CI]	Heterogeneity (I^2^%)	Publication bias-Egger test (P-value)
**Daily case growth rate**	Lockdown	5 (22)	-4.32% [-6.76 to—1.88]	99.9	0.008
	Social distancing	4(4)	-9.80% [-21.12 to 1.52	99.9	
	Stay at home	5(5)	-4.42% [-6.85 to -2	99.8	
	Other	3(5)	-3.27% [-7.67 to 1.13]	97.9	
	**Overall**	**14 (36)**	**-4.86% [-6.94to -2.78]**	**99.9**	
**Daily mortality growth rate**	Lockdown	3(9)	-7.29% [-13.14 to -1.44]	100	0.001
	Social distancing	2(2)	-0.09% [-1.57 to 1.75	92.5	
	Stay at home	2(2)	-1.42% [-2.46 to -0.37]	0.00	
	Other	2(2)	-6.37% [-16.47 to 3.73]	97.3	
	**Overall**	**7(15)**	**-4.87% [-8.34 to -1.40]**	**100**	
**Reproduction Number (R)**	Lockdown	4(14)	-1.61 [-1.97 to -1.25]	98.8	0.98
	Social distancing	2(10)	-2.36 [-2.81 to -1.91]	100	
	Other	2(2)	-0.86 [-1.78 to +0.06]	99.9	
	**Overall**	**6(17)**	**-1.90 [-2.23 to -1.58]**	**100**	
* Daily ICU admissions	**Overall**	**2(4)**	**-16.50% [-19.68 to -13.32]**	**98.9**	0.82

CI: Confidence Interval.

* Due to the small number of studies in this section, the subgroup analysis was not performed.

**Fig 3:**
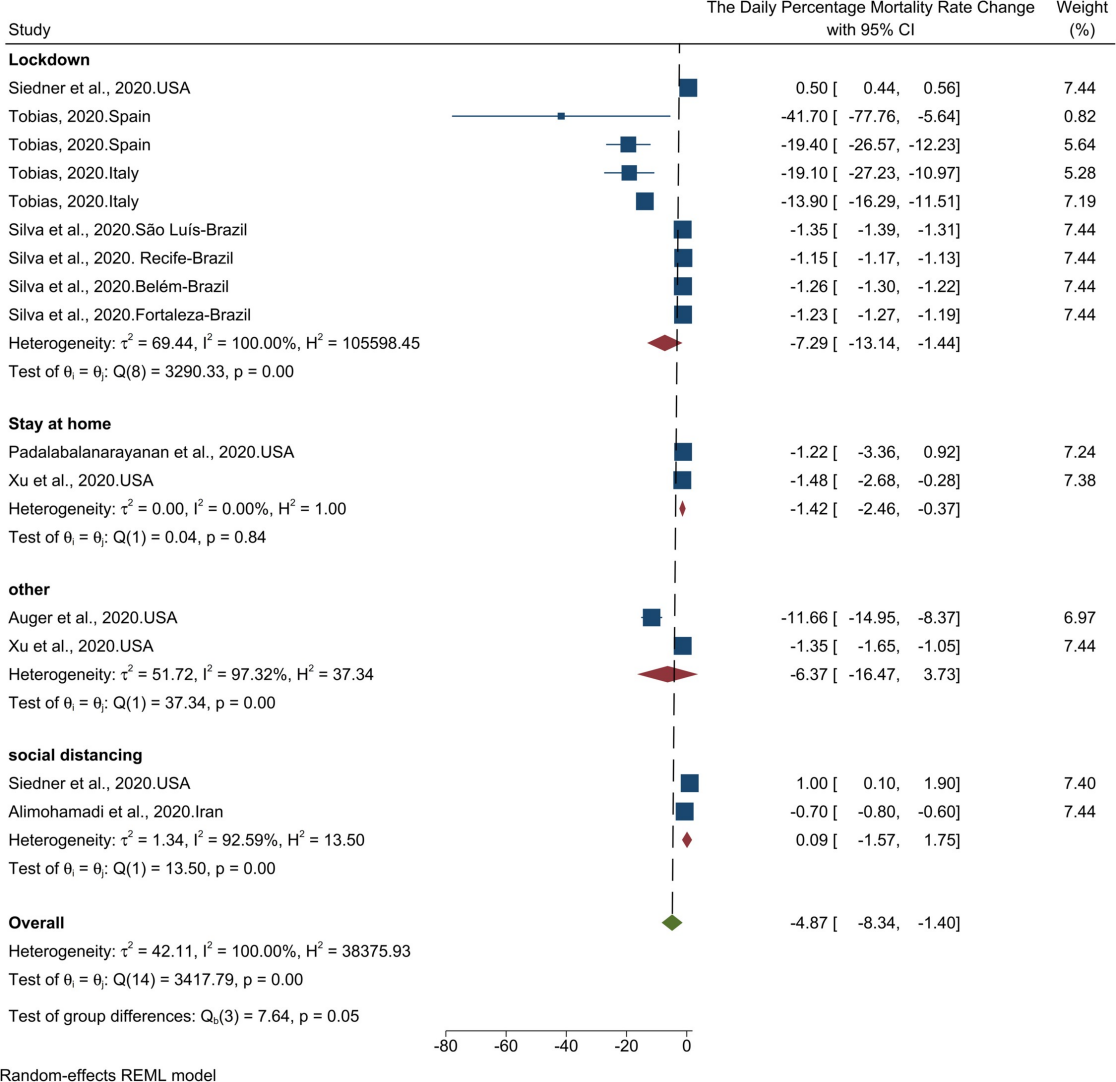
Results of the meta-analysis of the effect of NPHIs on the daily percentage changes of COVID-19 mortality growth rate in communities based on a random effect model.

The results of the meta-analysis of seven studies showed a 4.87% (95% CI, -8.34 to -1.40) decrease in daily death growth rate (Table 1). The results of the heterogeneity analysis showed that there is a high heterogeneity among studies. The results of the publication bias assessment indicated that there is a high probability of publication bias (Fig in S2 Fig). The results of the Trim-and-Fill test also showed that one case was probably missed so that by imputing that study and its effect, the size of the change would increase to -4.5 (95% CI, -8.05 to -1.08). Fig 3 illustrates the results of the effect of NPHIs on COVID-19 daily mortality growth rate.

**Fig 4:**
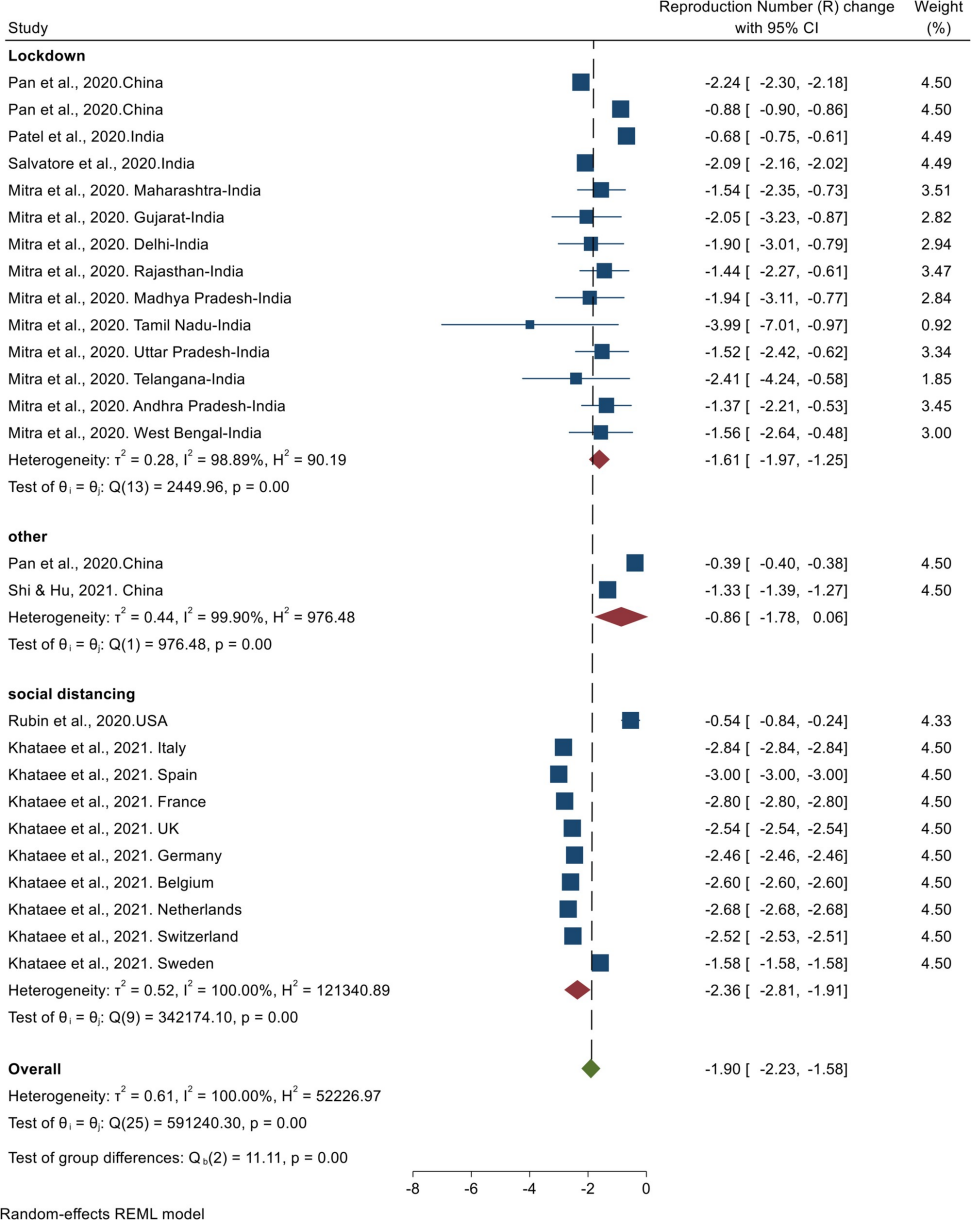
Results of the meta-analysis of the effects of NPHIs on changes in the Covid-19 reproduction number in the communities based on a random effect model.

The results of the meta-analysis of seven studies showed that NPHIs have resulted in a 1.90 (95% CI, -2.23 to -1.58) decrease in the COVID-19 reproduction number (Table 1). The results of the heterogeneity analysis showed high heterogeneity among studies. The results of the publication bias assessment showed no possibility of publication bias (Fig in S3 Fig). Fig 4 shows the results of the effect of NPHIs on the COVID-19 reproduction number.

**Fig 5:**
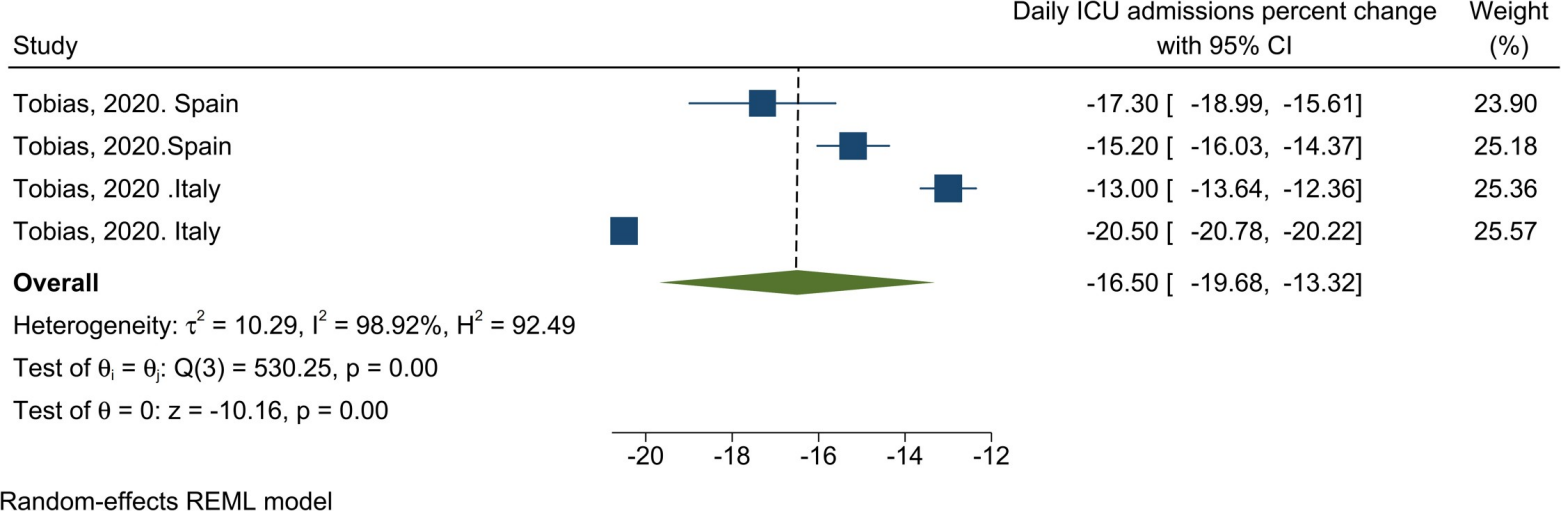
Results of the meta-analysis of the effects of NPHIs on the percentage of the changes in Covid-19 ICU admission in communities based on a random effect model.

The results of the meta-analysis of two studies showed a 16.50% (95% CI, -19.68 to -13.32) decrease in COVID-19 ICU admission as a result of NPHIs (Table 1). The results of the heterogeneity analysis showed high heterogeneity among studies. The results of the publication bias assessment showed no possibility of publication bias (Fig in S4 Fig). Fig 5 shows the results of the effect of NPHIs on COVID-19 daily ICU admission.

## Discussion

We conducted this systematic review and meta-analysis to address the effectiveness of various NPHIs in controlling the COVID-19 pandemic. Although we targeted experimental and quasi-experimental studies in the review, almost all included studies used interrupted times-series analysis to assess the impact of the NPHIs on controlling the unprecedented pandemics. Therefore, the results should be interpreted with caution because most studies could not have considered many causal factors when analyzing the data.

Most studies showed that NPHIs have been effective in controlling the spread of the disease and results of the meta-synthesis showed 4.9% decrease in case growth rate and death growth rate attributed to COVID-19, 16.5% decrease in ICU admission, and 1.4 change in reproduction number. Moreover, studies also showed that the spread of the disease increased after lifting the lockdown. All NPHIs have been shown to have positive effects on controlling the infection, however, it is not possible to figure out which intervention has been the most effective, due to complications regarding measuring the unique impact of implementing a specific measure [59].

We found several factors such as political, geographical, and epidemiological factors may influence the effectiveness of the NPHIs in the era of COVID-19 and above all is the timing of the interventions [60]. It is very important to contain the spread of the infection at the very early stage of the outbreak. At later stages, no NPHIs, even if implemented harshly, might be very effective [27]. For instance, in many Italian regions, a saturation of the ICUs was attributed to the late adoption of the NPHIs [27]. Moreover, studies showed that when authorities enforced NPHIs at the early stages of the pandemic, when the cumulative incidence of COVID-19 was slow, greater decreases in incidence and mortality of COVID-19 were shown [33,38,41,51,60]. The later NPHIs are adopted, the more stringent the interventions need to be to curb COVID-19 and would be anyways inadequate to prevent catastrophic outcomes. Additionally, communities that enacted early NPHIs, such as lockdown, visibly mitigated consequences even by experiencing a shorter intervention period [33], which is a very important point considering the highly destructive economic influence of lockdown. As a result, real-time detection of the changes in the pattern of the pandemic curve is crucial to evaluate the adopted interventions and plan for future pandemics [52].

Besides the timing of the interventions, other factors such as demographics characteristics and densities of population, mobility rates, variations in testing accessibility and testing strategies, and climate characteristics [44] contribute to the spread of the COVID-19 and may impact the effectiveness of NPHIs [39,61]. For example, studies from the US found that NPHIs are much more effective in reducing the COVID-19 growth rate in regions with larger populations or those with higher population density [39]. Another study from Spain corroborates these findings showing that despite the adoption of the lockdown, a higher daily mortality rate was most possibly attributed to larger demographic density and higher mobility rate before the lockdown [33]. Nevertheless, just a limited number of studies, and almost those from the US and China have considered these factors in the analysis. In addition, the impediment of the spread of COVID-19 could have been a result of the gradual growth of herd immunity in a community that makes it difficult to consider when evaluating the effects of the interventions [62]. In fact, alongside the geographic and demographic factors, disease factors such as acquired immunity after infections and the possibility of viral mutations determine some outcomes such as time-varying reproduction numbers [47]. All these factors make it complicated to have a precise judgment on the effectiveness of NPHIs.

Another point worth to be discussed in this systematic review is related to the role of the capacity of the countries’ health data infrastructures in conducting robust studies to assess the effectiveness of the NPHIs in pandemics. Collecting and analyzing data on infection transmission in different settings are necessary to evaluate the effectiveness of NPHIs [59]. Our systematic review highlights the critical role of data infrastructure in pandemics. The more robust the data infrastructure in a country is the more reliable and rigorous studies on the effectiveness of the NPHIs are. Robust studies may assist policymakers to guide their decisions in controlling the pandemics. In this systematic review, we realized that most studies were from European countries, the US, and China that have a relatively suitable data infrastructure. Most of these studies, especially those from the US, have had considered various types of covariates in the analysis that could have contributed to providing relatively strong proof of the effectiveness of the NPHIs. Studies from the US have used several sources of data, including Johns Hopkins COVID-19 interactive dashboard, Centers for Disease Control and Prevention (CDC), and state and local public health departments. Many included studies from low-resource countries, in contrast, have just used the publicly available data, whose validity and reliability are questionable. Data under-reporting due to the mass panic is the most possible reason to question the publicly available data from countries with poor data infrastructure. Even in the US some magnitude of underreporting for new cases and deaths was reported [49,63,64]. The capacity of a country to assess, monitor, and interpret health outcomes is considerably tailored to the existence of a robust data infrastructure. The importance of the health data infrastructure has been also proven in previous epidemics such as Ebola, Zika, and Influenza [11].

### Limitations

There are some limitations that we believe might have influenced the results of our systematic review. First and the main limitation was the observational nature of the included studies that could rule out causal inference [29,31]. Moreover, due to the unprecedented nature of the pandemic and ethical considerations, clinical trials were not possible under such public health emergencies [31]. Although including different types of covariates in the analysis could have alleviated this limitation, not all studies have used a completed number of possible covariates in the analysis. Second, another potential limitation is the possible confounding effect of COVID-19 diagnosis testing considerations on the outcomes. In many studies, the NPHIs coincided with an increased testing capacity which could question the observed epidemiological patterns across the study. Unfortunately, only a few studies addressed this issue and examined the possible effects of the changes in testing capacity on the desirable outcomes [41]. Third, as we discussed earlier, the number of confirmed cases is dependent on the country’s testing strategies and the number of tests performed. Unless the number of tests performed on suspected individuals around a country is known, the certainty of the country’s actual total number of infected individuals is under question [40]. Fourth, NPHIs are highly dependent on contextual factors. Nevertheless, in the meta-analysis, we dismissed contextual factors and merged the results of the interventions from different countries. Finally, we excluded non-English language studies as well as the studies with incomplete information to calculate the effect size from the analysis. Despite all these limitations, this systematic review provides a comprehensive insight into the potential impact of the NPHIs on controlling the COVID-19 pandemics.

## Conclusion

The majority of NPHIs had positive effects on restraining the COVID-19 spread. We found significant decreases in COVID-19 case growth rate, death growth rate, and reproduction number during and in the later stage of the lockdown. However, it was challenging for countries to maintain this path after the lockdown was lifted. The early enforcement of lockdown, when the incidence rate is not high, can contribute to a shorter duration of lockdown and a lower increase of the case growth rate in the post-lockdown era.

Considering the negative impact of the nationwide lockdown and other harsh restrictions on the economy and people’s life, such interventions should be mitigated by adopting other NPHIs such as mass mask-wearing, patient/suspected case isolation strategies, and contact tracing. With the problems that remain regarding universal access to vaccines and their effectiveness, more public health strategies are needed not only to flatten the epidemic curve but to maintain it flat as well. This is particularly important when thinking of preparing for upcoming pandemic waves and future epidemics. The results of this systematic review and meta-analysis could aid policymakers in planning future public health interventions and is vital to understand the impact of NPHIs adopted by countries.

Although almost all studies showed significant, positive effects of NPHIs on controlling the COVID-19 pandemic, we cannot dismiss the side impact of stringent restricting interventions on people, such as compliance with medications, healthy lifestyle habits among patients with non-communicable disease, delay in cancer diagnosis, and mental health problems. Further research using a wide range of covariates are needed to evaluate the effectiveness of NPHIs in different countries. Studies need to address the impact of NPHIs on the population’s other health problems than COVID-19 as well.

## Supporting information

S1 Fig Results of the funnel plot, daily percentage growth rate change. (TIF)

S2 Fig Results of the funnel plot, daily percentage mortality rate change. (TIF)

S3 Fig Results of the funnel plot, reproduction number change. (TIF)

S4 Fig Results of the funnel plot, daily ICU admissions percent change. (TIF)

S1 Table Description of interventions. (DOCX)

S2 Table Search strategies. (DOCX)

S3 Table Extraction table. (DOCX)

S4 Table Results of the EPOK risk of bias assessment for interrupted time series studies. (DOCX)

S5 Table Results of the EPOK risk of bias assessment for studies with a separate control group. (DOCX)

S6 Table PRISMA 2020 checklist. (DOCX)
