# Supplementary figures and images for: Effectiveness of non-pharmaceutical public health interventions against COVID-19: A systematic review and meta-analysis
Source: PLoS One. 2021 Nov 23;16(11):e0260371. doi: 10.1371/journal.pone.0260371 (PMC8610259; doi:10.1371/journal.pone.0260371)

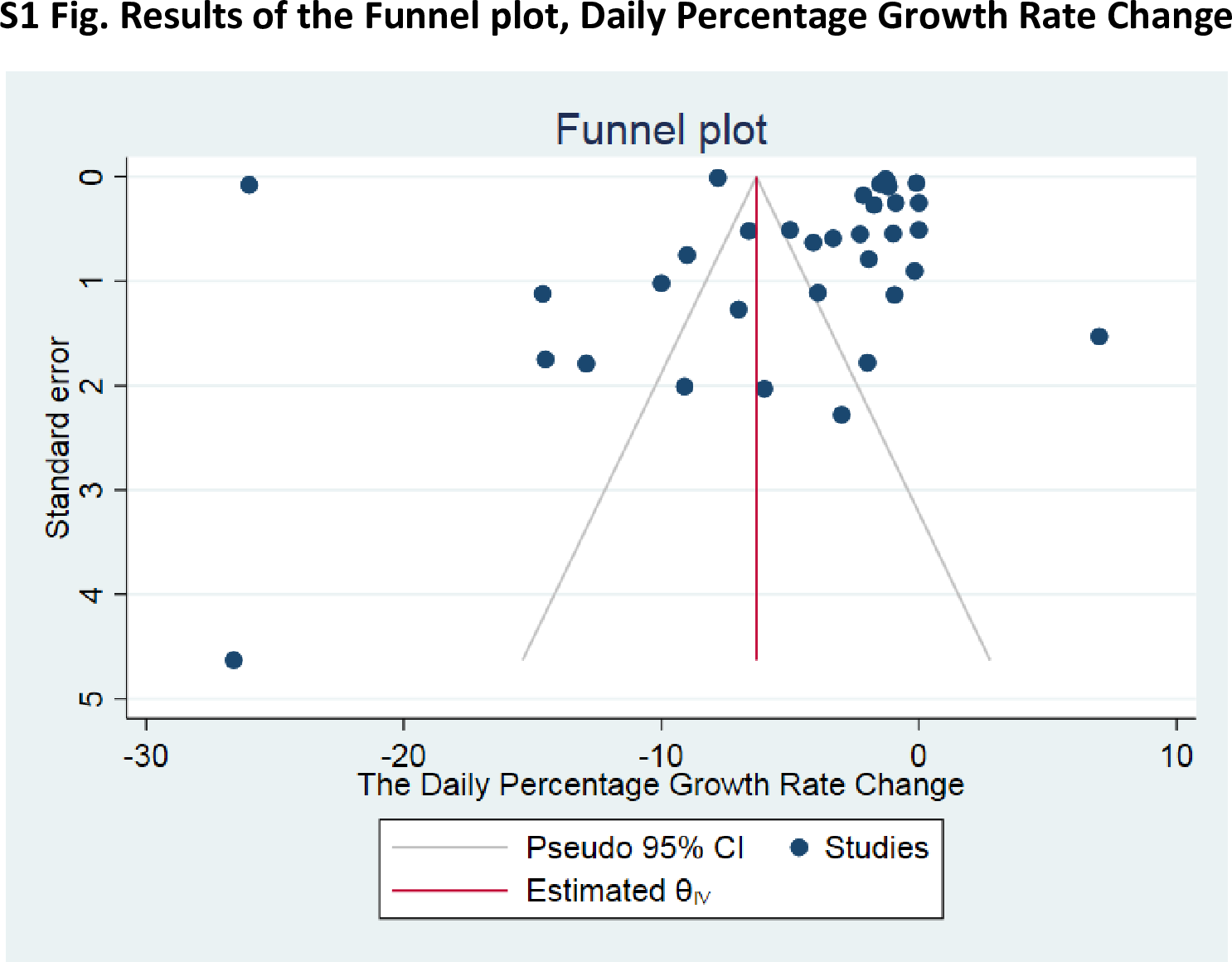

Supplement: S1 Fig — (TIF) [file pone.0260371.s001.tif]

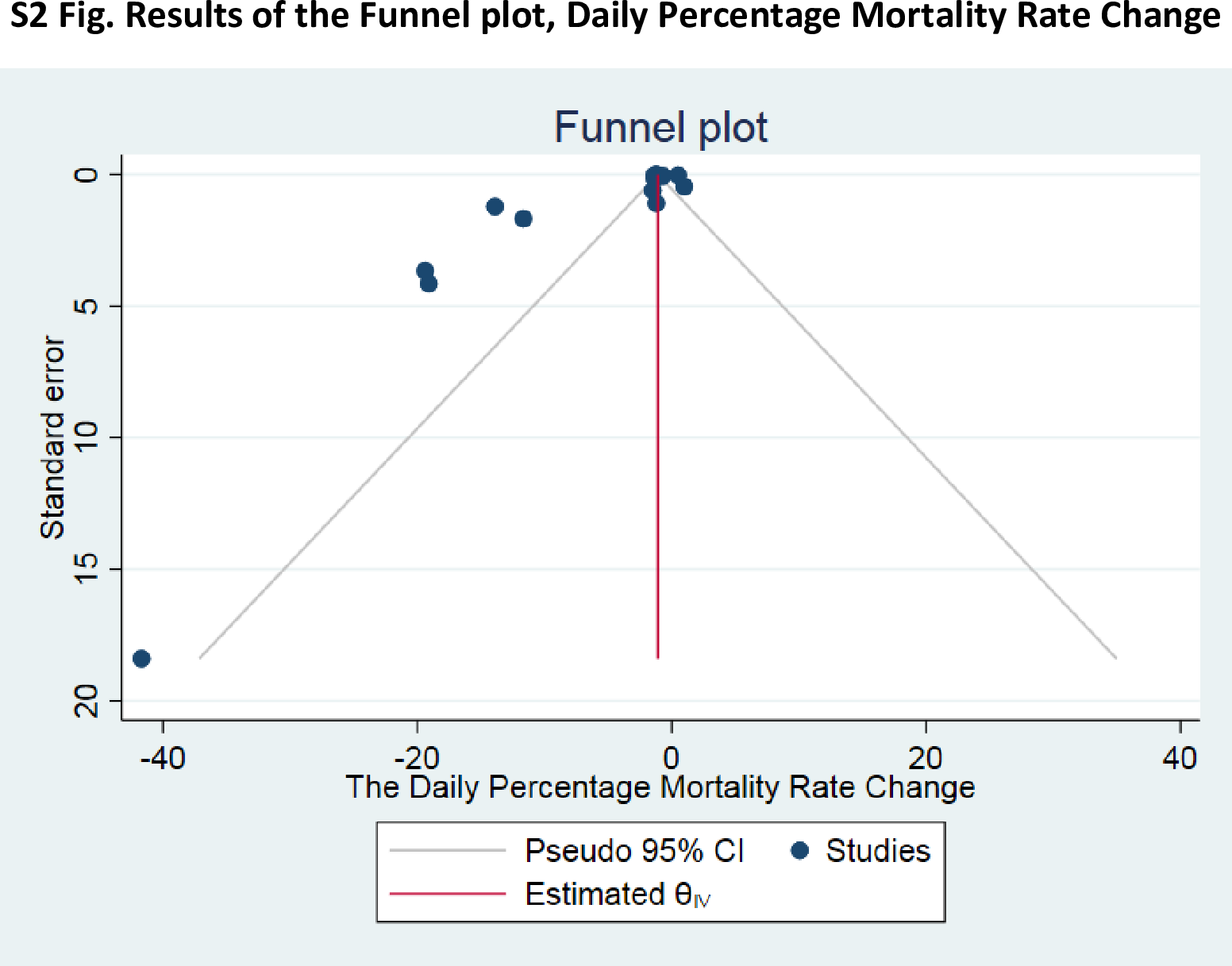

Supplement: S2 Fig — (TIF) [file pone.0260371.s002.tif]

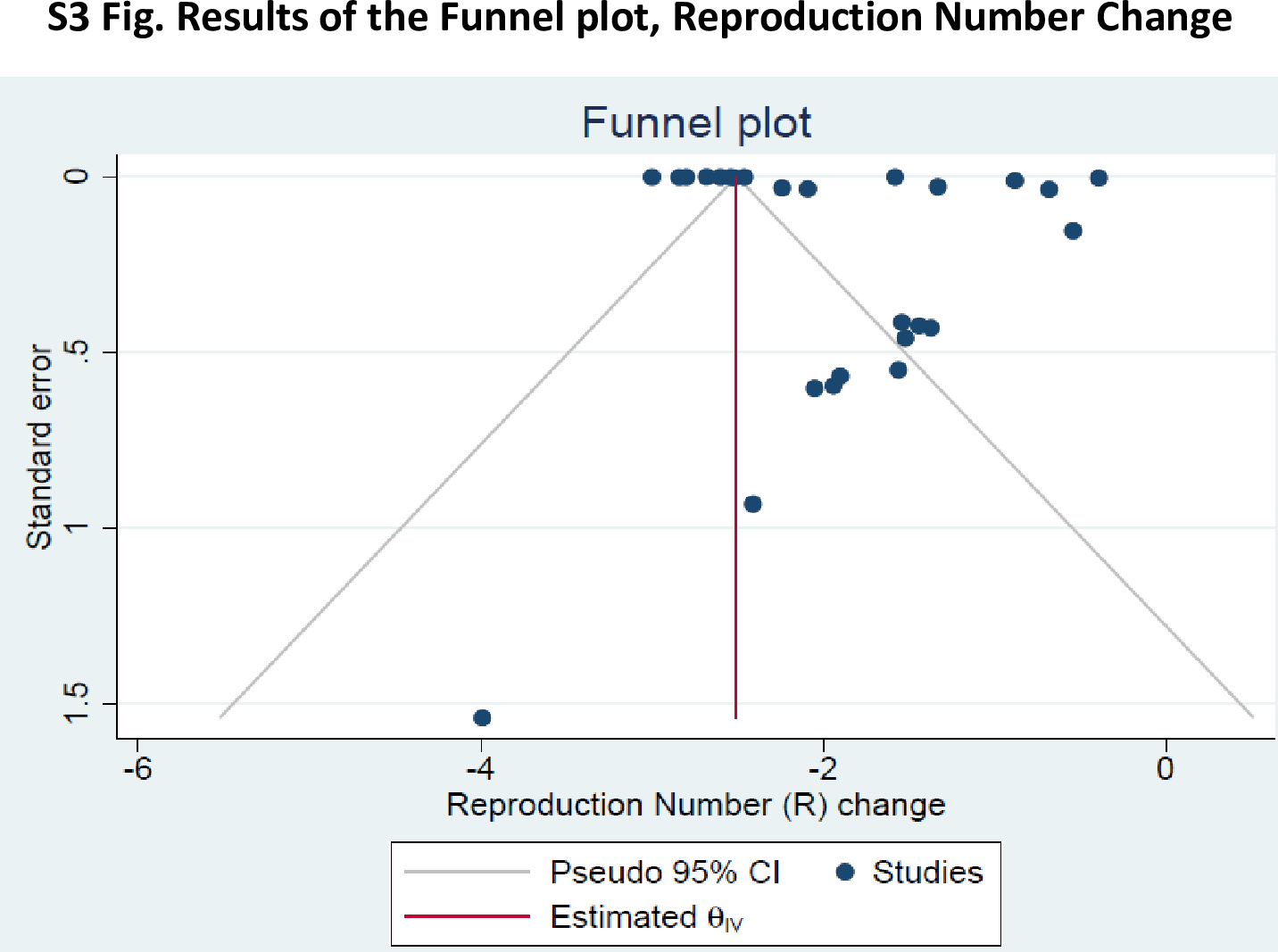

Supplement: S3 Fig — (TIF) [file pone.0260371.s003.tif]

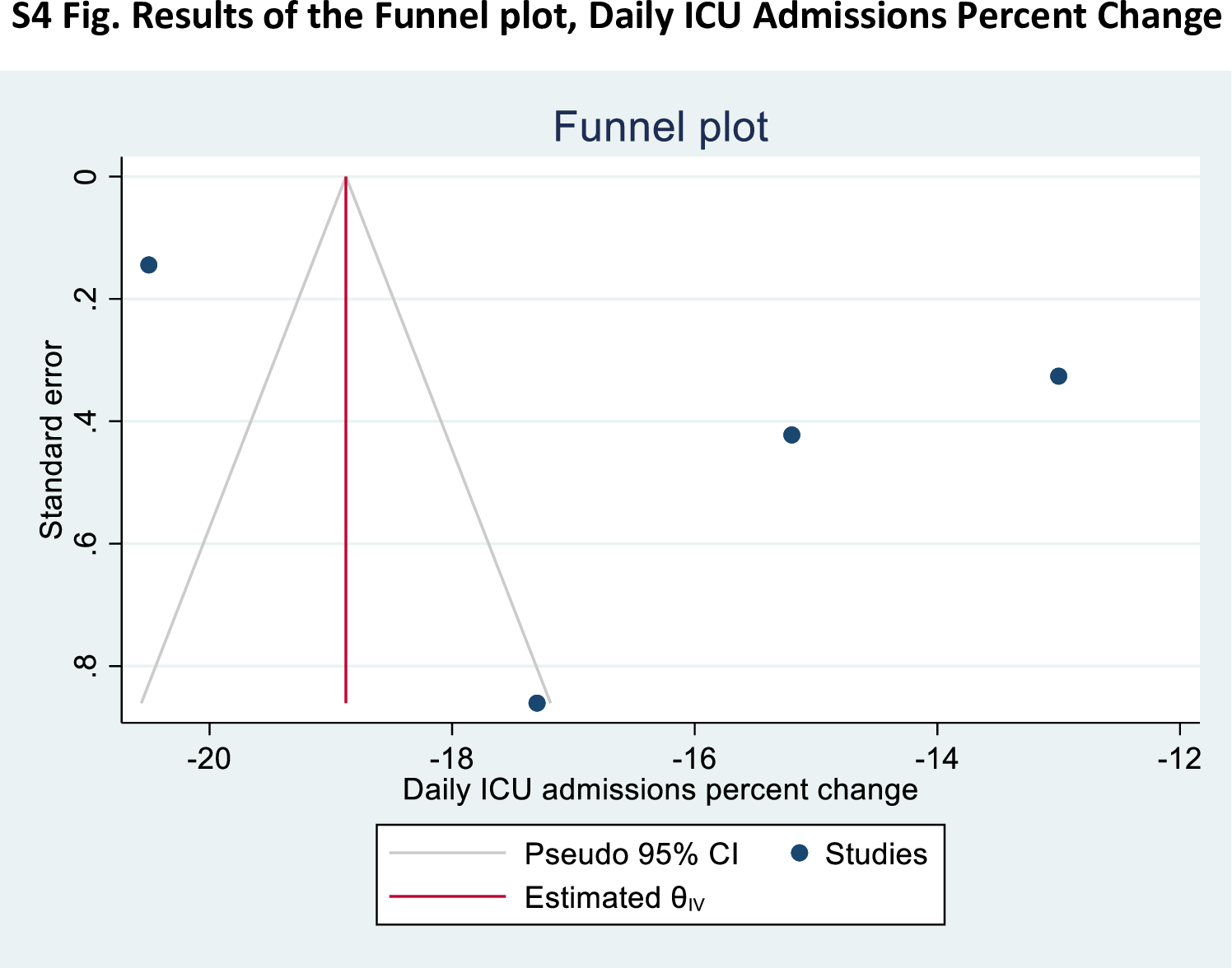

Supplement: S4 Fig — (TIF) [file pone.0260371.s004.tif]
