# Supplementary material for: Effectiveness of non-pharmaceutical public health interventions against COVID-19: A systematic review and meta-analysis
Source: PLoS One. 2021 Nov 23;16(11):e0260371. doi: 10.1371/journal.pone.0260371 (PMC8610259; doi:10.1371/journal.pone.0260371)
Supplement: S1 Table — (DOCX) [file pone.0260371.s005.docx]

**S1 Table: Description of interventions**

| NPHIs | Descriptions |
| --- | --- |
| Social distancing | Social distancing, also known as “physical distancing,” means keeping a safe space between ones another who are not from their household (["Guidance for Unvaccinated People; Social Distancing," 2020](#_ENREF_2)). although Literature have provided broad definition of social distancing measures ranging from closures of schools, closures of workplaces, cancellations of public events, restrictions on internal movement, and closures of state borders ([Siedner et al., 2020](#_ENREF_4)). Different authorities have defined different standards for social distancing. However, studies included in this systematic review did not reported the standards were used in them. Therefore in this review, we considered each intervention as social distancing if the study named that as social distancing. |
| Lockdown | Restrictions on internal movement often referred to colloquially as “lockdowns” are generally the most restrictive measures enforced by government to keep residents at home to minimize the contacts between people ([Siedner et al., 2020](#_ENREF_4)). That is, large scale physical distancing measures and movement restrictions are often referred to as ‘lockdowns’(["Coronavirus disease (COVID-19): Herd immunity, lockdowns and COVID-19," 2020](#_ENREF_1)). |
| Stay-at-home orders | Stay-at-home orders are a community mitigation strategy used to reduce the spread of COVID-19 in the United States. This rule restricts movements of a population as a mass quarantine strategy for suppressing or mitigating the pandemic by ordering residents to stay home except for essential tasks or for work in essential businesses ([Moreland et al., 2020](#_ENREF_3)). |
| other | We classified interventions such as shelter in place, mask-wearing, mass screening, and universal symptom survey that were had not similarity to any of other categories and for which there were not adequate studies to conduct meta-analysis separately, into a single category namely other. |
| References  Coronavirus disease (COVID-19): Herd immunity, lockdowns and COVID-19. (2020). 2021, from https://[www.who.int/news-room/q-a-detail/herd-immunity-lockdowns-and-covid-19](http://www.who.int/news-room/q-a-detail/herd-immunity-lockdowns-and-covid-19)  Guidance for Unvaccinated People; Social Distancing. (2020). 2021, from https://[www.cdc.gov/coronavirus/2019-ncov/prevent-getting-sick/social-distancing.html](http://www.cdc.gov/coronavirus/2019-ncov/prevent-getting-sick/social-distancing.html)  Moreland, A., et al. (2020). Timing of state and territorial COVID-19 stay-at-home orders and changes in population movement—United States, March 1–May 31, 2020. *Morbidity and Mortality Weekly Report, 69*(35), 1198.  Siedner, M. J., et al. (2020). Social distancing to slow the US COVID-19 epidemic: Longitudinal pretest-posttest comparison group study. *PLoS Medicine, 17*(8), 1-12. doi: 10.1371/journal.pmed.1003244 | |
