# Supplementary material for: Effectiveness of non-pharmaceutical public health interventions against COVID-19: A systematic review and meta-analysis
Source: PLoS One. 2021 Nov 23;16(11):e0260371. doi: 10.1371/journal.pone.0260371 (PMC8610259; doi:10.1371/journal.pone.0260371)
Supplement: S2 Table — (DOCX) [file pone.0260371.s006.docx]

**S2 Table: Search strategies (results of the primary search conducted on November 11, 2020)**

PubMed

| **R** | **Command** | **Strategies and keywords** | **Results** |
| --- | --- | --- | --- |
| #1 |  | **Search: (((((((((((((("2019 novel coronavirus"[Title/Abstract]) OR ("COVID19"[Title/Abstract])) OR ("COVID-19"[Title/Abstract])) OR ("COVID 2019"[Title/Abstract])) OR ("2019-novel CoV"[Title/Abstract])) OR ("SARS-cov-2"[Title/Abstract])) OR ("SARS-CoV2"[Title/Abstract])) OR ("SARSCoV2"[Title/Abstract])) OR ("SARSCoV-2"[Title/Abstract])) OR ("2019-ncov"[Title/Abstract])) OR ("coronavirus disease 2019"[Title/Abstract])) OR ("coronavirus disease-19"[Title/Abstract])) OR ("2019ncov"[Title/Abstract])) OR ("SARS coronavirus 2"[Title/Abstract])) OR ("severe acute respiratory syndrome coronavirus 2"[Title/Abstract])** | [72,103](https://pubmed.ncbi.nlm.nih.gov/?term=%28%28%28%28%28%28%28%28%28%28%28%28%28%28%222019+novel+coronavirus%22%5BTitle%2FAbstract%5D%29+OR+%28%22COVID19%22%5BTitle%2FAbstract%5D%29%29+OR+%28%22COVID-19%22%5BTitle%2FAbstract%5D%29%29+OR+%28%22COVID+2019%22%5BTitle%2FAbstract%5D%29%29+OR+%28%222019-novel+CoV%22%5BTitle%2FAbstract%5D%29%29+OR+%28%22SARS-cov-2%22%5BTitle%2FAbstract%5D%29%29+OR+%28%22SARS-CoV2%22%5BTitle%2FAbstract%5D%29%29+OR+%28%22SARSCoV2%22%5BTitle%2FAbstract%5D%29%29+OR+%28%22SARSCoV-2%22%5BTitle%2FAbstract%5D%29%29+OR+%28%222019-ncov%22%5BTitle%2FAbstract%5D%29%29+OR+%28%22coronavirus+disease+2019%22%5BTitle%2FAbstract%5D%29%29+OR+%28%22coronavirus+disease-19%22%5BTitle%2FAbstract%5D%29%29+OR+%28%222019ncov%22%5BTitle%2FAbstract%5D%29%29+OR+%28%22SARS+coronavirus+2%22%5BTitle%2FAbstract%5D%29%29+OR+%28%22severe+acute+respiratory+syndrome+coronavirus+2%22%5BTitle%2FAbstract%5D%29&sort=) |
| #2 |  | **Search: ("severe acute respiratory syndrome coronavirus 2"[Supplementary Concept]) OR ("COVID-19"[Supplementary Concept])** | [36,869](https://pubmed.ncbi.nlm.nih.gov/?term=%28%22severe+acute+respiratory+syndrome+coronavirus+2%22%5BSupplementary+Concept%5D%29+OR+%28%22COVID-19%22%5BSupplementary+Concept%5D%29&sort=) |
| #3 |  | **Search: (("severe acute respiratory syndrome coronavirus 2"[Supplementary Concept]) OR ("COVID-19"[Supplementary Concept])) OR ((((((((((((((("2019 novel coronavirus"[Title/Abstract]) OR ("COVID19"[Title/Abstract])) OR ("COVID-19"[Title/Abstract])) OR ("COVID 2019"[Title/Abstract])) OR ("2019-novel CoV"[Title/Abstract])) OR ("SARS-cov-2"[Title/Abstract])) OR ("SARS-CoV2"[Title/Abstract])) OR ("SARSCoV2"[Title/Abstract])) OR ("SARSCoV-2"[Title/Abstract])) OR ("2019-ncov"[Title/Abstract])) OR ("coronavirus disease 2019"[Title/Abstract])) OR ("coronavirus disease-19"[Title/Abstract])) OR ("2019ncov"[Title/Abstract])) OR ("SARS coronavirus 2"[Title/Abstract])) OR ("severe acute respiratory syndrome coronavirus 2"[Title/Abstract]))** | [73,845](https://pubmed.ncbi.nlm.nih.gov/?term=%28%28%22severe+acute+respiratory+syndrome+coronavirus+2%22%5BSupplementary+Concept%5D%29+OR+%28%22COVID-19%22%5BSupplementary+Concept%5D%29%29+OR+%28%28%28%28%28%28%28%28%28%28%28%28%28%28%28%222019+novel+coronavirus%22%5BTitle%2FAbstract%5D%29+OR+%28%22COVID19%22%5BTitle%2FAbstract%5D%29%29+OR+%28%22COVID-19%22%5BTitle%2FAbstract%5D%29%29+OR+%28%22COVID+2019%22%5BTitle%2FAbstract%5D%29%29+OR+%28%222019-novel+CoV%22%5BTitle%2FAbstract%5D%29%29+OR+%28%22SARS-cov-2%22%5BTitle%2FAbstract%5D%29%29+OR+%28%22SARS-CoV2%22%5BTitle%2FAbstract%5D%29%29+OR+%28%22SARSCoV2%22%5BTitle%2FAbstract%5D%29%29+OR+%28%22SARSCoV-2%22%5BTitle%2FAbstract%5D%29%29+OR+%28%222019-ncov%22%5BTitle%2FAbstract%5D%29%29+OR+%28%22coronavirus+disease+2019%22%5BTitle%2FAbstract%5D%29%29+OR+%28%22coronavirus+disease-19%22%5BTitle%2FAbstract%5D%29%29+OR+%28%222019ncov%22%5BTitle%2FAbstract%5D%29%29+OR+%28%22SARS+coronavirus+2%22%5BTitle%2FAbstract%5D%29%29+OR+%28%22severe+acute+respiratory+syndrome+coronavirus+2%22%5BTitle%2FAbstract%5D%29%29&sort=) |
| #4 |  | **Search: (((((((((("Non-pharmaceutical intervention*"[Title/Abstract]) OR ("Public heath intervention*"[Title/Abstract])) OR (Isolat*[Title/Abstract])) OR (quarantin*[Title/Abstract])) OR (Lockdown[Title/Abstract])) OR ("lock down"[Title/Abstract])) OR ("social distanc*"[Title/Abstract])) OR ("physical distanc*"[Title/Abstract])) OR ("Patient isolation"[MeSH Terms])) OR ("quarantine"[MeSH Terms])) OR ("social distance"[MeSH Terms])** | [1,375,128](https://pubmed.ncbi.nlm.nih.gov/?term=%28%28%28%28%28%28%28%28%28%28%22Non-pharmaceutical+intervention%2A%22%5BTitle%2FAbstract%5D%29+OR+%28%22Public+heath+intervention%2A%22%5BTitle%2FAbstract%5D%29%29+OR+%28Isolat%2A%5BTitle%2FAbstract%5D%29%29+OR+%28quarantin%2A%5BTitle%2FAbstract%5D%29%29+OR+%28Lockdown%5BTitle%2FAbstract%5D%29%29+OR+%28%22lock+down%22%5BTitle%2FAbstract%5D%29%29+OR+%28%22social+distanc%2A%22%5BTitle%2FAbstract%5D%29%29+OR+%28%22physical+distanc%2A%22%5BTitle%2FAbstract%5D%29%29+OR+%28%22Patient+isolation%22%5BMeSH+Terms%5D%29%29+OR+%28%22quarantine%22%5BMeSH+Terms%5D%29%29+OR+%28%22social+distance%22%5BMeSH+Terms%5D%29&sort=) |
| #5 |  | **Search: ((((((((((((("community containment"[Title/Abstract]) OR ("containment area"[Title/Abstract])) OR (Suppress*[Title/Abstract])) OR (Mitigate*[Title/Abstract])) OR ("contact trac*"[Title/Abstract])) OR ("partner notification*"[Title/Abstract])) OR ("Media* report*"[Title/Abstract])) OR ("contact tracing"[MeSH Terms])) OR (stay* home[Title/Abstract])) OR (stay* at home[Title/Abstract])) OR (travel* ban[Title/Abstract])) OR (avoid crowd* area*[Title/Abstract])) OR (protect* material*[Title/Abstract])) OR (Mask*[Title/Abstract])** | [924,760](https://pubmed.ncbi.nlm.nih.gov/?term=%28%28%28%28%28%28%28%28%28%28%28%28%28%22community+containment%22%5BTitle%2FAbstract%5D%29+OR+%28%22containment+area%22%5BTitle%2FAbstract%5D%29%29+OR+%28Suppress%2A%5BTitle%2FAbstract%5D%29%29+OR+%28Mitigate%2A%5BTitle%2FAbstract%5D%29%29+OR+%28%22contact+trac%2A%22%5BTitle%2FAbstract%5D%29%29+OR+%28%22partner+notification%2A%22%5BTitle%2FAbstract%5D%29%29+OR+%28%22Media%2A+report%2A%22%5BTitle%2FAbstract%5D%29%29+OR+%28%22contact+tracing%22%5BMeSH+Terms%5D%29%29+OR+%28stay%2A+home%5BTitle%2FAbstract%5D%29%29+OR+%28stay%2A+at+home%5BTitle%2FAbstract%5D%29%29+OR+%28travel%2A+ban%5BTitle%2FAbstract%5D%29%29+OR+%28avoid+crowd%2A+area%2A%5BTitle%2FAbstract%5D%29%29+OR+%28protect%2A+material%2A%5BTitle%2FAbstract%5D%29%29+OR+%28Mask%2A%5BTitle%2FAbstract%5D%29&sort=) |
| #6 | #5 OR #4 | **Search: (((((((((((((("community containment"[Title/Abstract]) OR ("containment area"[Title/Abstract])) OR (Suppress*[Title/Abstract])) OR (Mitigate*[Title/Abstract])) OR ("contact trac*"[Title/Abstract])) OR ("partner notification*"[Title/Abstract])) OR ("Media* report*"[Title/Abstract])) OR ("contact tracing"[MeSH Terms])) OR (stay* home[Title/Abstract])) OR (stay* at home[Title/Abstract])) OR (travel* ban[Title/Abstract])) OR (avoid crowd* area*[Title/Abstract])) OR (protect* material*[Title/Abstract])) OR (Mask*[Title/Abstract])) OR ((((((((((("Non-pharmaceutical intervention*"[Title/Abstract]) OR ("Public heath intervention*"[Title/Abstract])) OR (Isolat*[Title/Abstract])) OR (quarantin*[Title/Abstract])) OR (Lockdown[Title/Abstract])) OR ("lock down"[Title/Abstract])) OR ("social distanc*"[Title/Abstract])) OR ("physical distanc*"[Title/Abstract])) OR ("Patient isolation"[MeSH Terms])) OR ("quarantine"[MeSH Terms])) OR ("social distance"[MeSH Terms]))** | [2,242,985](https://pubmed.ncbi.nlm.nih.gov/?term=%28%28%28%28%28%28%28%28%28%28%28%28%28%28%22community+containment%22%5BTitle%2FAbstract%5D%29+OR+%28%22containment+area%22%5BTitle%2FAbstract%5D%29%29+OR+%28Suppress%2A%5BTitle%2FAbstract%5D%29%29+OR+%28Mitigate%2A%5BTitle%2FAbstract%5D%29%29+OR+%28%22contact+trac%2A%22%5BTitle%2FAbstract%5D%29%29+OR+%28%22partner+notification%2A%22%5BTitle%2FAbstract%5D%29%29+OR+%28%22Media%2A+report%2A%22%5BTitle%2FAbstract%5D%29%29+OR+%28%22contact+tracing%22%5BMeSH+Terms%5D%29%29+OR+%28stay%2A+home%5BTitle%2FAbstract%5D%29%29+OR+%28stay%2A+at+home%5BTitle%2FAbstract%5D%29%29+OR+%28travel%2A+ban%5BTitle%2FAbstract%5D%29%29+OR+%28avoid+crowd%2A+area%2A%5BTitle%2FAbstract%5D%29%29+OR+%28protect%2A+material%2A%5BTitle%2FAbstract%5D%29%29+OR+%28Mask%2A%5BTitle%2FAbstract%5D%29%29+OR+%28%28%28%28%28%28%28%28%28%28%28%22Non-pharmaceutical+intervention%2A%22%5BTitle%2FAbstract%5D%29+OR+%28%22Public+heath+intervention%2A%22%5BTitle%2FAbstract%5D%29%29+OR+%28Isolat%2A%5BTitle%2FAbstract%5D%29%29+OR+%28quarantin%2A%5BTitle%2FAbstract%5D%29%29+OR+%28Lockdown%5BTitle%2FAbstract%5D%29%29+OR+%28%22lock+down%22%5BTitle%2FAbstract%5D%29%29+OR+%28%22social+distanc%2A%22%5BTitle%2FAbstract%5D%29%29+OR+%28%22physical+distanc%2A%22%5BTitle%2FAbstract%5D%29%29+OR+%28%22Patient+isolation%22%5BMeSH+Terms%5D%29%29+OR+%28%22quarantine%22%5BMeSH+Terms%5D%29%29+OR+%28%22social+distance%22%5BMeSH+Terms%5D%29%29&sort=) |
| #7 |  | **Search: (((((((((((((((((Mortality[Title/Abstract]) OR (Morbidity[Title/Abstract])) OR (Incidence[Title/Abstract])) OR (Prevalence[Title/Abstract])) OR (Hospitaliz*[Title/Abstract])) OR ("intensive care"[Title/Abstract])) OR ("ICU hospitalization"[Title/Abstract])) OR ("intensive care hospitalization"[Title/Abstract])) OR (new case*[Title/Abstract])) OR ("new case*"[Title/Abstract])) OR (basic reproduction number[Title/Abstract])) OR ("basic reproduction number"[Title/Abstract])) OR ("incidence"[MeSH Terms])) OR ("prevalence"[MeSH Terms])) OR ("mortality"[MeSH Terms])) OR ("morbidity"[MeSH Terms])) OR ("hospitalization"[MeSH Terms])) OR ("basic reproduction number"[MeSH Terms])** | [2,836,399](https://pubmed.ncbi.nlm.nih.gov/?term=%28%28%28%28%28%28%28%28%28%28%28%28%28%28%28%28%28Mortality%5BTitle%2FAbstract%5D%29+OR+%28Morbidity%5BTitle%2FAbstract%5D%29%29+OR+%28Incidence%5BTitle%2FAbstract%5D%29%29+OR+%28Prevalence%5BTitle%2FAbstract%5D%29%29+OR+%28Hospitaliz%2A%5BTitle%2FAbstract%5D%29%29+OR+%28%22intensive+care%22%5BTitle%2FAbstract%5D%29%29+OR+%28%22ICU+hospitalization%22%5BTitle%2FAbstract%5D%29%29+OR+%28%22intensive+care+hospitalization%22%5BTitle%2FAbstract%5D%29%29+OR+%28new+case%2A%5BTitle%2FAbstract%5D%29%29+OR+%28%22new+case%2A%22%5BTitle%2FAbstract%5D%29%29+OR+%28basic+reproduction+number%5BTitle%2FAbstract%5D%29%29+OR+%28%22basic+reproduction+number%22%5BTitle%2FAbstract%5D%29%29+OR+%28%22incidence%22%5BMeSH+Terms%5D%29%29+OR+%28%22prevalence%22%5BMeSH+Terms%5D%29%29+OR+%28%22mortality%22%5BMeSH+Terms%5D%29%29+OR+%28%22morbidity%22%5BMeSH+Terms%5D%29%29+OR+%28%22hospitalization%22%5BMeSH+Terms%5D%29%29+OR+%28%22basic+reproduction+number%22%5BMeSH+Terms%5D%29&sort=) |
| #8 | #7 AND #6 AND #3 | **Search: (((((((((((((((((((Mortality[Title/Abstract]) OR (Morbidity[Title/Abstract])) OR (Incidence[Title/Abstract])) OR (Prevalence[Title/Abstract])) OR (Hospitaliz*[Title/Abstract])) OR ("intensive care"[Title/Abstract])) OR ("ICU hospitalization"[Title/Abstract])) OR ("intensive care hospitalization"[Title/Abstract])) OR (new case*[Title/Abstract])) OR ("new case*"[Title/Abstract])) OR (basic reproduction number[Title/Abstract])) OR ("basic reproduction number"[Title/Abstract])) OR ("incidence"[MeSH Terms])) OR ("prevalence"[MeSH Terms])) OR ("mortality"[MeSH Terms])) OR ("morbidity"[MeSH Terms])) OR ("hospitalization"[MeSH Terms])) OR ("basic reproduction number"[MeSH Terms])) AND ((((((((((((((("community containment"[Title/Abstract]) OR ("containment area"[Title/Abstract])) OR (Suppress*[Title/Abstract])) OR (Mitigate*[Title/Abstract])) OR ("contact trac*"[Title/Abstract])) OR ("partner notification*"[Title/Abstract])) OR ("Media* report*"[Title/Abstract])) OR ("contact tracing"[MeSH Terms])) OR (stay* home[Title/Abstract])) OR (stay* at home[Title/Abstract])) OR (travel* ban[Title/Abstract])) OR (avoid crowd* area*[Title/Abstract])) OR (protect* material*[Title/Abstract])) OR (Mask*[Title/Abstract])) OR ((((((((((("Non-pharmaceutical intervention*"[Title/Abstract]) OR ("Public heath intervention*"[Title/Abstract])) OR (Isolat*[Title/Abstract])) OR (quarantin*[Title/Abstract])) OR (Lockdown[Title/Abstract])) OR ("lock down"[Title/Abstract])) OR ("social distanc*"[Title/Abstract])) OR ("physical distanc*"[Title/Abstract])) OR ("Patient isolation"[MeSH Terms])) OR ("quarantine"[MeSH Terms])) OR ("social distance"[MeSH Terms])))) AND ((("severe acute respiratory syndrome coronavirus 2"[Supplementary Concept]) OR ("COVID-19"[Supplementary Concept])) OR ((((((((((((((("2019 novel coronavirus"[Title/Abstract]) OR ("COVID19"[Title/Abstract])) OR ("COVID-19"[Title/Abstract])) OR ("COVID 2019"[Title/Abstract])) OR ("2019-novel CoV"[Title/Abstract])) OR ("SARS-cov-2"[Title/Abstract])) OR ("SARS-CoV2"[Title/Abstract])) OR ("SARSCoV2"[Title/Abstract])) OR ("SARSCoV-2"[Title/Abstract])) OR ("2019-ncov"[Title/Abstract])) OR ("coronavirus disease 2019"[Title/Abstract])) OR ("coronavirus disease-19"[Title/Abstract])) OR ("2019ncov"[Title/Abstract])) OR ("SARS coronavirus 2"[Title/Abstract])) OR ("severe acute respiratory syndrome coronavirus 2"[Title/Abstract])))** | [3,061](https://pubmed.ncbi.nlm.nih.gov/?term=longquery2d6043d1f444c7fe0cf5&sort=) |
|  | Final | ENGLUSH | [**(2,970)**](https://pubmed.ncbi.nlm.nih.gov/?term=longquery73924350ebb3c32bbbff&filter=dates.2019%2F12%2F15-2020%2F11%2F15&ac=no&sort=date) |

CINAHL

| **R** | **Command** | **Strategies and keywords** | **Results** |
| --- | --- | --- | --- |
| S1 |  | TI "2019 novel coronavirus" OR TI "COVID19" OR TI "COVID-19" OR TI "COVID 2019" OR TI "2019-novel CoV" OR TI "SARS-cov-2" OR TI "SARS-CoV2" OR TI "SARSCoV2" OR TI "SARSCoV-2" | (18,084) |
| S2 |  | AB "2019 novel coronavirus" OR AB "COVID19" OR AB "COVID-19" OR AB "COVID 2019" OR AB "2019-novel CoV" OR AB "SARS-cov-2" OR AB "SARS-CoV2" OR AB "SARSCoV2" OR AB "SARSCoV-2" | (13,698) |
| S3 |  | TI "2019-ncov" OR TI "coronavirus disease 2019" OR TI "coronavirus disease-19" OR TI "2019ncov" OR TI "SARS coronavirus 2" OR TI "severe acute respiratory syndrome coronavirus 2" OR AB "2019-ncov" OR AB "coronavirus disease 2019" OR AB "coronavirus disease-19" OR AB "2019ncov" OR AB "SARS coronavirus 2" OR AB "severe acute respiratory syndrome coronavirus 2" | (4,505) |
| S4 |  | MW "COVID-19" | (14,500) |
| S5 | S1 OR S2 OR S3 OR S4 |  | (27,243) |
| S6 |  | TI "non-pharmaceutical intervention*" OR TI "public heath intervention*" OR TI isolat* OR TI quarantin* OR TI lockdown OR TI "lock down" OR AB "non-pharmaceutical intervention*" OR AB "public heath intervention*" OR AB isolat* OR AB quarantin* OR AB lockdown OR AB "lock down" | (98,747) |
| S7 |  | TI "social distanc*" OR TI "physical distanc*" OR TI "community containment" OR TI "containment area" OR TI suppress* OR TI mitigate* OR AB "social distanc*" OR AB "physical distanc*" OR AB "community containment" OR AB "containment area" OR AB suppress* OR AB mitigate* | (63,246) |
| S8 |  | TI "contact trac*" OR TI "partner notification*" OR TI "media* report*" OR TI stay* home OR TI stay* at home OR TI travel* ban OR AB "contact trac*" OR AB "partner notification*" OR AB "media* report*" OR AB stay* home OR AB stay* at home OR AB travel* ban | (3,033) |
| S9 |  | TI avoid crowd* area* OR TI protect* material* OR TI mask* OR AB avoid crowd* area* OR AB protect* material* OR AB mask* | (15,846) |
| S10 |  | MW "patient isolation" OR MW "quarantine" OR MW "social distance" OR MW "contact tracing" | (5,702) |
| S11 | S6 OR S7 OR S8 OR S9 OR S10 |  | (179,688) |
| S12 |  | TI mortality OR TI morbidity OR TI incidence OR TI prevalence OR TI hospitaliz* OR TI "intensive care" OR AB mortality OR AB morbidity OR AB incidence OR AB prevalence OR AB hospitaliz* OR AB "intensive care" | (621,668) |
| S13 |  | TI "ICU hospitalization" OR TI "intensive care hospitalization" OR TI new case* OR TI "new case*" OR TI basic reproduction number OR TI "basic reproduction number" OR AB "ICU hospitalization" OR AB "intensive care hospitalization" OR AB new case* OR AB "new case*" OR AB basic reproduction number OR AB "basic reproduction number" | (4,532) |
| S14 |  | MW "incidence" OR MW "prevalence" OR MW "mortality" OR MW "morbidity" OR MW "hospitalization" OR MW "basic reproduction number" | (366,918) |
| S15 |  | S12 OR S13 OR S14 | (785,702) |
| S16 |  | S5 AND S11 AND S15 | (855) |
|  | Final | Limiters - Published Date: 20191201-; Language: English  Expanders - Apply equivalent subjects ; Search modes - Boolean/Phrase | **831** |

Web of Science (ISI)

| **R** | **Command** | **Strategies and keywords** | **Results** |
| --- | --- | --- | --- |
| #1 |  | **TOPIC:** ("2019 novel coronavirus") *OR* **TOPIC:** ("COVID19") *OR* **TOPIC:** ("COVID-19") *OR* **TOPIC:** ("COVID 2019") *OR* **TOPIC:** ("2019-novel CoV") *OR* **TOPIC:** ("SARS-cov-2") *OR* **TOPIC:** ("SARS-CoV2") *OR* **TOPIC:** ("SARSCoV2") *OR* **TOPIC:** ("SARSCoV-2") *OR* **TOPIC:** ("severe acute respiratory syndrome coronavirus 2") *OR* **TOPIC:** ("2019-ncov") *OR* **TOPIC:** ("coronavirus disease 2019") *OR* **TOPIC:** ("2019ncov") | **48,302** |
| #2 |  | **TOPIC:** ("non-pharmaceutical intervention*") *OR* **TOPIC:** ("public heath intervention*") *OR* **TOPIC:** (isolat*) *OR* **TOPIC:** (quarantin*) *OR* **TOPIC:** (lockdown) *OR* **TOPIC:** ("lock down") *OR* **TOPIC:** ("social distanc*") *OR* **TOPIC:** ("physical distanc*") *OR* **TOPIC:** ("community containment") *OR* **TOPIC:** ("containment area") *OR* **TOPIC:** (suppress*) *OR* **TOPIC:** (mitigate*) *OR* **TOPIC:** ("contact trac*") *OR* **TOPIC:** ("partner notification*") *OR* **TOPIC:** ("media* report*") *OR* **TOPIC:** (stay* home) *OR* **TOPIC:** (stay* at home) *OR* **TOPIC:** (travel* ban) *OR* **TOPIC:** (avoid crowd* area*) *OR* **TOPIC:** (protect* material*) *OR* **TOPIC:** (mask*) | **2,793,212** |
| #3 |  | **TOPIC:** (mortality) *OR* **TOPIC:** (morbidity) *OR* **TOPIC:** (incidence) *OR* **TOPIC:** (prevalence) *OR* **TOPIC:** (hospitaliz*) *OR* **TOPIC:** ("intensive care") *OR* **TOPIC:** ("ICU hospitalization") *OR* **TOPIC:** ("intensive care hospitalization") *OR* **TOPIC:** (new case*) *OR* **TOPIC:** ("new case*") *OR* **TOPIC:** ("basic reproduction number") | **3,023,313** |
| #4 | #1 AND #2 AND #3 |  | **2,052** |
| #5 |  | #3  AND  #2  AND  #1  **Refined by:** **DOCUMENT TYPES:** ( ARTICLE OR EARLY ACCESS ) AND **LANGUAGES:** ( ENGLISH ) | **1,603** |

EMBASE

| **R** | **Command** | **Strategies and keywords** | **Results** |
| --- | --- | --- | --- |
| #1 |  | 'coronavirus disease 2019'/de OR '2019 novel coronavirus':ti,ab,kw OR 'covid19':ti,ab,kw OR 'covid-19':ti,ab,kw OR 'covid 2019':ti,ab,kw OR '2019-novel cov':ti,ab,kw OR 'sars-cov-2':ti,ab,kw OR 'sars-cov2':ti,ab,kw OR 'sarscov2':ti,ab,kw OR '2019-ncov':ti,ab,kw OR 'coronavirus disease 2019':ti,ab,kw OR '2019ncov':ti,ab,kw OR 'severe acute respiratory syndrome coronavirus 2':ti,ab,kw | **75,002** |
| #2 |  | 'non-pharmaceutical intervention*':ti,ab,kw OR 'public heath intervention*':ti,ab,kw OR isolat*:ti,ab,kw OR 'patient isolation':lnk OR quarantin*:ti,ab,kw OR quarantine:lnk OR lockdown:ti,ab,kw OR 'lock down':ti,ab,kw OR 'social distanc*':ti,ab,kw OR 'social distance':lnk OR 'physical distanc*':ti,ab,kw OR 'community containment':ti,ab,kw OR 'containment area':ti,ab,kw OR suppress*:ti,ab,kw OR mitigate*:ti,ab,kw OR 'contact trac*':ti,ab,kw OR 'partner notification':ti,ab,kw OR 'partner notification':lnk OR 'media* report*':ti,ab,kw OR 'stay* home':ti,ab,kw OR 'stay* at home':ti,ab,kw OR 'travel* ban':ti,ab,kw OR 'avoid crowd* area*':ti,ab,kw OR 'protect* material*':ti,ab,kw OR 'face mask':ti,ab,kw | **2,543,299** |
| #3 |  | mortality:ti,ab,kw OR mortality:lnk OR morbidity:ti,ab,kw OR morbidity:lnk OR incidence:ti,ab,kw OR incidence:lnk OR prevalence:ti,ab,kw OR prevalence:lnk OR hospitaliz*:ti,ab,kw OR hospitalization:lnk OR 'intensive care':ti,ab,kw OR 'icu hospitalization':ti,ab,kw OR 'intensive care hospitalization':ti,ab,kw OR 'new case*':ti,ab,kw OR 'basic reproduction number':ti,ab,kw OR 'basic reproduction number':lnk | **3,402,112** |
| #4 | #1 AND #2 AND #3 |  | **2,350** |
| #5 | #1 AND #2 AND #3 | #1 AND #2 AND #3 AND ([article]/lim OR [article in press]/lim OR [conference abstract]/lim OR [conference paper]/lim OR [conference review]/lim OR [data papers]/lim) AND [english]/lim AND [embase]/lim AND [2019-2020]/py | **1,356** |

SCOPUS

| **R** | **Command** | **Strategies and keywords** | **Results** |
| --- | --- | --- | --- |
| #1 |  | ( TITLE-ABS-KEY ( *"2019 novel coronavirus"* )  OR  TITLE-ABS-KEY ( *"COVID19"* )  OR  TITLE-ABS-KEY ( *"COVID-19"* )  OR  TITLE-ABS-KEY ( *"COVID 2019"* )  OR  TITLE-ABS-KEY ( *"2019-novel CoV"* )  OR  TITLE-ABS-KEY ( *"SARS-cov-2"* )  OR  TITLE-ABS-KEY ( *"SARSCoV2"* )  OR  TITLE-ABS-KEY ( *"SARS-CoV2"* )  OR  TITLE-ABS-KEY ( *"2019-ncov"* )  OR  TITLE-ABS-KEY ( *"coronavirus disease 2019"* )  OR  TITLE-ABS-KEY ( *"coronavirus disease-19"* )  OR  TITLE-ABS-KEY ( *"2019ncov"* )  OR  TITLE-ABS-KEY ( *"severe acute respiratory syndrome coronavirus 2"* ) ) | **78,275** |
| #2 |  | ( TITLE-ABS-KEY ( *"non-pharmaceutical intervention*"* )  OR  TITLE-ABS-KEY ( *"public heath intervention*"* )  OR  TITLE-ABS-KEY ( *isolat** )  OR  TITLE-ABS-KEY ( *quarantin** )  OR  TITLE-ABS-KEY ( *lockdown* )  OR  TITLE-ABS-KEY ( *"lock down"* )  OR  TITLE-ABS-KEY ( *"social distanc*"* )  OR  TITLE-ABS-KEY ( *"physical distanc*"* )  OR  TITLE-ABS-KEY ( *"community containment"* )  OR  TITLE-ABS-KEY ( *"containment area"* )  OR  TITLE-ABS-KEY ( *suppress** )  OR  TITLE-ABS-KEY ( *mitigate** )  OR  TITLE-ABS-KEY ( *"contact trac*"* )  OR  TITLE-ABS-KEY ( *"partner notification*"* )  OR  TITLE-ABS-KEY ( *media**  AND *report** )  OR  TITLE-ABS-KEY ( *stay**  AND *home* )  OR  TITLE-ABS-KEY ( *stay**  AND *at*  AND *home* )  OR  TITLE-ABS-KEY ( *travel**  AND *ban* )  OR  TITLE-ABS-KEY ( *avoid*  AND *crowd**  AND *area** )  OR  TITLE-ABS-KEY ( *protect**  AND *material** )  OR  TITLE-ABS-KEY ( *mask** ) ) | **5,149,627** |
| #3 |  | ( TITLE-ABS-KEY ( *mortality* )  OR  TITLE-ABS-KEY ( *morbidity* )  OR  TITLE-ABS-KEY ( *incidence* )  OR  TITLE-ABS-KEY ( *prevalence* )  OR  TITLE-ABS-KEY ( *hospitaliz** )  OR  TITLE-ABS-KEY ( *"intensive care"* )  OR  TITLE-ABS-KEY ( *"ICU hospitalization"* )  OR  TITLE-ABS-KEY ( *"intensive care hospitalization"* )  OR  TITLE-ABS-KEY ( *"new case*"* )  OR  TITLE-ABS-KEY ( *"basic reproduction number"* ) ) | **4,021,282** |
| #4 | #1 AND #2 AND #3 | ( ( TITLE-ABS-KEY ( *"2019 novel coronavirus"* )  OR  TITLE-ABS-KEY ( *"COVID19"* )  OR  TITLE-ABS-KEY ( *"COVID-19"* )  OR  TITLE-ABS-KEY ( *"COVID 2019"* )  OR  TITLE-ABS-KEY ( *"2019-novel CoV"* )  OR  TITLE-ABS-KEY ( *"SARS-cov-2"* )  OR  TITLE-ABS-KEY ( *"SARSCoV2"* )  OR  TITLE-ABS-KEY ( *"SARS-CoV2"* )  OR  TITLE-ABS-KEY ( *"2019-ncov"* )  OR  TITLE-ABS-KEY ( *"coronavirus disease 2019"* )  OR  TITLE-ABS-KEY ( *"coronavirus disease-19"* )  OR  TITLE-ABS-KEY ( *"2019ncov"* )  OR  TITLE-ABS-KEY ( *"severe acute respiratory syndrome coronavirus 2"* ) ) )  AND  ( ( TITLE-ABS-KEY ( *"non-pharmaceutical intervention*"* )  OR  TITLE-ABS-KEY ( *"public heath intervention*"* )  OR  TITLE-ABS-KEY ( *isolat** )  OR  TITLE-ABS-KEY ( *quarantin** )  OR  TITLE-ABS-KEY ( *lockdown* )  OR  TITLE-ABS-KEY ( *"lock down"* )  OR  TITLE-ABS-KEY ( *"social distanc*"* )  OR  TITLE-ABS-KEY ( *"physical distanc*"* )  OR  TITLE-ABS-KEY ( *"community containment"* )  OR  TITLE-ABS-KEY ( *"containment area"* )  OR  TITLE-ABS-KEY ( *suppress** )  OR  TITLE-ABS-KEY ( *mitigate** )  OR  TITLE-ABS-KEY ( *"contact trac*"* )  OR  TITLE-ABS-KEY ( *"partner notification*"* )  OR  TITLE-ABS-KEY ( *media**  AND *report** )  OR  TITLE-ABS-KEY ( *stay**  AND *home* )  OR  TITLE-ABS-KEY ( *stay**  AND *at*  AND *home* )  OR  TITLE-ABS-KEY ( *travel**  AND *ban* )  OR  TITLE-ABS-KEY ( *avoid*  AND *crowd**  AND *area** )  OR  TITLE-ABS-KEY ( *protect**  AND *material** )  OR  TITLE-ABS-KEY ( *mask** ) ) )  AND  ( ( TITLE-ABS-KEY ( *mortality* )  OR  TITLE-ABS-KEY ( *morbidity* )  OR  TITLE-ABS-KEY ( *incidence* )  OR  TITLE-ABS-KEY ( *prevalence* )  OR  TITLE-ABS-KEY ( *hospitaliz** )  OR  TITLE-ABS-KEY ( *"intensive care"* )  OR  TITLE-ABS-KEY ( *"ICU hospitalization"* )  OR  TITLE-ABS-KEY ( *"intensive care hospitalization"* )  OR  TITLE-ABS-KEY ( *"new case*"* )  OR  TITLE-ABS-KEY ( *"basic reproduction number"* ) ) ) | **6,771** |
| #5 |  | ( LIMIT-TO ( DOCTYPE ,  *"ar"* )  OR  LIMIT-TO ( DOCTYPE ,  *"sh"* )  OR  LIMIT-TO ( DOCTYPE ,  *"cp"* ) )  AND  ( LIMIT-TO ( LANGUAGE ,  *"English"* ) ) | **4,081** |

PROQUEST

| **R** | **Command** | **Strategies and keywords** | **Results** |
| --- | --- | --- | --- |
| S1 |  | ti("2019 novel coronavirus") OR ti("COVID19") OR ti("COVID-19") OR ti("COVID 2019") OR ti("2019-novel CoV") OR ti("SARS-cov-2") OR ti("SARS-CoV2") OR ti("SARSCoV2") OR ti("coronavirus disease 2019") OR ti("severe acute respiratory syndrome coronavirus 2") | [**25,234**](https://search.proquest.com/recentsearches.recentsearchtabview.recentsearchesgridview.scrolledrecentsearchlist.checkdbssearchlink_0:rerunsearch/165CA61F343948F4PQ/None?t:ac=RecentSearches) |
| S2 |  | ab("2019 novel coronavirus") OR ab("COVID19") OR ab("COVID-19") OR ab("COVID 2019") OR ab("2019-novel CoV") OR ab("SARS-cov-2") OR ab("SARS-CoV2") OR ab("SARSCoV2") OR ab("coronavirus disease 2019") OR ab("severe acute respiratory syndrome coronavirus 2") | **19,637** |
| S3 |  | su(COVID-19) | **18,065** |
| S4 | 1 OR 2 OR 3 | (ti("2019 novel coronavirus") OR ti("COVID19") OR ti("COVID-19") OR ti("COVID 2019") OR ti("2019-novel CoV") OR ti("SARS-cov-2") OR ti("SARS-CoV2") OR ti("SARSCoV2") OR ti("coronavirus disease 2019") OR ti("severe acute respiratory syndrome coronavirus 2")) OR (ab("2019 novel coronavirus") OR ab("COVID19") OR ab("COVID-19") OR ab("COVID 2019") OR ab("2019-novel CoV") OR ab("SARS-cov-2") OR ab("SARS-CoV2") OR ab("SARSCoV2") OR ab("coronavirus disease 2019") OR ab("severe acute respiratory syndrome coronavirus 2")) OR su(COVID-19) | **30,261** |
| S5 |  | ti("non-pharmaceutical intervention*") OR ab("non-pharmaceutical intervention*") OR ti(isolat*) OR ti(quarantin*) OR ti(lockdown ) OR ti("lock down") OR ab(isolat*) OR ab(quarantin*) OR ab(lockdown ) OR ab("lock down") | **129,771** |
| S6 |  | ti("public heath intervention*") OR ab("public heath intervention*") OR ti("social distanc*") OR ti("physical distanc*") OR ti("community containment") OR ti("containment area") OR ab("social distanc*") OR ab("physical distanc*") OR ab("community containment") OR ab("containment area") | **2,289** |
| S7 |  | ti(suppress*) OR ti(mitigate*) OR ti("contact trac*") OR ti("partner notification*") OR ti("media* report*") OR ab(suppress*) OR ab(mitigate*) OR ab("contact trac*") OR ab("partner notification*") OR ab("media* report*") | **128,691** |
| S8 |  | ti(stay* at home) OR ti(travel* ban) OR ti(avoid crowd* area*) OR ti(protect* material*) OR ti(mask*) OR ab(stay* at home) OR ab(travel* ban) OR ab(avoid crowd* area*) OR ab(protect* material*) OR ab(mask*) | **25,058** |
| S9 |  | mainsubject(patient isolation) OR mainsubject(quarantine) OR mainsubject(social distance) OR mainsubject(contact tracing) | **1,889** |
| S10 | 5 OR 6 OR 7 OR 8 OR 9 | (ti("non-pharmaceutical intervention*") OR ab("non-pharmaceutical intervention*") OR ti(isolat*) OR ti(quarantin*) OR ti(lockdown) OR ti("lock down") OR ab(isolat*) OR ab(quarantin*) OR ab(lockdown) OR ab("lock down")) OR (ti("public heath intervention*") OR ab("public heath intervention*") OR ti("social distanc*") OR ti("physical distanc*") OR ti("community containment") OR ti("containment area") OR ab("social distanc*") OR ab("physical distanc*") OR ab("community containment") OR ab("containment area")) OR (ti(suppress*) OR ti(mitigate*) OR ti("contact trac*") OR ti("partner notification*") OR ti("media* report*") OR ab(suppress*) OR ab(mitigate*) OR ab("contact trac*") OR ab("partner notification*") OR ab("media* report*")) OR (ti(stay* at home) OR ti(travel* ban) OR ti(avoid crowd* area*) OR ti(protect* material*) OR ti(mask*) OR ab(stay* at home) OR ab(travel* ban) OR ab(avoid crowd* area*) OR ab(protect* material*) OR ab(mask*)) OR (mainsubject(patient isolation) OR mainsubject(quarantine) OR mainsubject(social distance) OR mainsubject(contact tracing)) | **278,462** |
| S11 |  | ti(mortality) OR ti(morbidity) OR ti(incidence) OR ti(prevalence) OR ti(hospitaliz*) OR ab(mortality) OR ab(morbidity) OR ab(incidence) OR ab(prevalence) OR ab(hospitaliz*) | **224,104** |
| S12 |  | ti( "intensive care") OR ti( "ICU hospitalization") OR ti("intensive care hospitalization") OR ti("new case*") OR ti("basic reproduction number") OR ab( "intensive care") OR ab( "ICU hospitalization") OR ab("intensive care hospitalization") OR ab("new case*") OR ab("basic reproduction number") | **15,925** |
| S13 |  | mainsubject("incidence") OR mainsubject("incidence") OR mainsubject("prevalence") OR mainsubject("mortality") OR mainsubject("morbidity") OR mainsubject("hospitalization") OR mainsubject("basic reproduction number") | **102,693** |
| S14 | 13 OR 12 OR 11 | (mainsubject("incidence") OR mainsubject("incidence") OR mainsubject("prevalence") OR mainsubject("mortality") OR mainsubject("morbidity") OR mainsubject("hospitalization") OR mainsubject("basic reproduction number")) OR (ti("intensive care") OR ti("ICU hospitalization") OR ti("intensive care hospitalization") OR ti("new case*") OR ti("basic reproduction number") OR ab("intensive care") OR ab("ICU hospitalization") OR ab("intensive care hospitalization") OR ab("new case*") OR ab("basic reproduction number")) OR (ti(mortality) OR ti(morbidity) OR ti(incidence) OR ti(prevalence) OR ti(hospitaliz*) OR ab(mortality) OR ab(morbidity) OR ab(incidence) OR ab(prevalence) OR ab(hospitaliz*)) | **269,402** |
| S15 | 14 AND 10 AND 4 | ((mainsubject("incidence") OR mainsubject("incidence") OR mainsubject("prevalence") OR mainsubject("mortality") OR mainsubject("morbidity") OR mainsubject("hospitalization") OR mainsubject("basic reproduction number")) OR (ti("intensive care") OR ti("ICU hospitalization") OR ti("intensive care hospitalization") OR ti("new case*") OR ti("basic reproduction number") OR ab("intensive care") OR ab("ICU hospitalization") OR ab("intensive care hospitalization") OR ab("new case*") OR ab("basic reproduction number")) OR (ti(mortality) OR ti(morbidity) OR ti(incidence) OR ti(prevalence) OR ti(hospitaliz*) OR ab(mortality) OR ab(morbidity) OR ab(incidence) OR ab(prevalence) OR ab(hospitaliz*))) AND ((ti("non-pharmaceutical intervention*") OR ab("non-pharmaceutical intervention*") OR ti(isolat*) OR ti(quarantin*) OR ti(lockdown) OR ti("lock down") OR ab(isolat*) OR ab(quarantin*) OR ab(lockdown) OR ab("lock down")) OR (ti("public heath intervention*") OR ab("public heath intervention*") OR ti("social distanc*") OR ti("physical distanc*") OR ti("community containment") OR ti("containment area") OR ab("social distanc*") OR ab("physical distanc*") OR ab("community containment") OR ab("containment area")) OR (ti(suppress*) OR ti(mitigate*) OR ti("contact trac*") OR ti("partner notification*") OR ti("media* report*") OR ab(suppress*) OR ab(mitigate*) OR ab("contact trac*") OR ab("partner notification*") OR ab("media* report*")) OR (ti(stay* at home) OR ti(travel* ban) OR ti(avoid crowd* area*) OR ti(protect* material*) OR ti(mask*) OR ab(stay* at home) OR ab(travel* ban) OR ab(avoid crowd* area*) OR ab(protect* material*) OR ab(mask*)) OR (mainsubject(patient isolation) OR mainsubject(quarantine) OR mainsubject(social distance) OR mainsubject(contact tracing))) AND ((ti("2019 novel coronavirus") OR ti("COVID19") OR ti("COVID-19") OR ti("COVID 2019") OR ti("2019-novel CoV") OR ti("SARS-cov-2") OR ti("SARS-CoV2") OR ti("SARSCoV2") OR ti("coronavirus disease 2019") OR ti("severe acute respiratory syndrome coronavirus 2")) OR (ab("2019 novel coronavirus") OR ab("COVID19") OR ab("COVID-19") OR ab("COVID 2019") OR ab("2019-novel CoV") OR ab("SARS-cov-2") OR ab("SARS-CoV2") OR ab("SARSCoV2") OR ab("coronavirus disease 2019") OR ab("severe acute respiratory syndrome coronavirus 2")) OR su(COVID-19)) | **1,193** |
| S16 |  | Applied filters  Your results have been filtered. Skip to [first result](https://search.proquest.com/results/7946FA5EF1554FCDPQ/1?accountid=41307#item_1).  [Clear all filters](https://search.proquest.com/results.filteredby:unfilterall?t:ac=7946FA5EF1554FCDPQ/1)  • • 2019-12-15 - 2020  • • • Article  • • • • • English | **754 results** |
