# Supplementary material for: Effectiveness of non-pharmaceutical public health interventions against COVID-19: A systematic review and meta-analysis
Source: PLoS One. 2021 Nov 23;16(11):e0260371. doi: 10.1371/journal.pone.0260371 (PMC8610259; doi:10.1371/journal.pone.0260371)
Supplement: S3 Table — (DOCX) [file pone.0260371.s007.docx]

**S3 Table: Extraction Table**

1. **Correlation of COVID-19 Pandemic with Healthcare System Response and Prevention Measures in Saudi Arabia (Adly et al., 2020)**

| Data Collection Item | Item description / sub-groups | |
| --- | --- | --- |
| Bibiliographic Information | | |
| Author | Adly et al. | |
| Title | Correlation of COVID-19 Pandemic with Healthcare System Response and Prevention Measures in Saudi Arabia | |
| Year of publication | 2020 | |
| Country | Saudi Arabia | |
|  | | |
| Methods | | |
| Type of Study | Observational | |
| Time period and setting of the study | From 1February to 27 July 2020; Saudi Arabia community. | |
| Intervention | - To prevent the entrance of about 2.5 million international pilgrims seeking to perform hajj; - Offering free testing for residents whether Saudi and non-Saudi; - Travel ban; - Compulsory quarantine for travellers; - Avoid mass gathering. | |
| description of intervention | - The prevention platform for COVID-19 has been performed in the Saudi Arabia community since January 15, 2020. - In Saudi Arabia, several preventative measures starting on February 26. - Despite these limitations, on March 2, Saudi Arabia reported its first COVID-19 confirmed case for a traveller returning from Iran via Bahrain without declaring their travel history to Iran. - On March 4, Umrah was totally suspended and all pilgrimage travel plans were cancelled until further notice and the two holy mosques in Makkah and Madinah were set to completely close for cleaning and sterilization by March 5. - On March 8, the Saudi government transferred schools and universities to distance learning and simulated classrooms. This was complemented by a travel prohibition to all countries and setting a compulsory quarantine for travelers who arrived from affected countries. - By March 12, all social and governmental crowds and social events were banned or postponed including the Saudi African and Arab-African summits. | |
| setting of the intervention | Public places | |
| Aim/ Objectives | To evaluate the spread of COVID-19 associated with preventive measures taken in Saudi Arabia and to assess the COVID-19 mortality rate related to pandemic control actions taken by healthcare systems in Saudi Arabia and to develop a detailed COVID-19 prevention protocol as a framework to the Saudi Arabian community. | |
| Participant characteristics | **participants** | - Total population of 34,813,871 - Density of 16 people per km2 estimated on an overall land-living area of 2,149,690 km2 - Elderly (65+ years) represents 81.3%. (vary geographically and also by sex) - Cardiovascular death rate was 42% and diabetes prevalence was 17.7%. The percentage of male and female smokers was 25.4% and 1.8%, respectively. - In Saudi Arabia, the hospital bed number per thousand is 2.7 with a Saudi life expectancy of 75.13 years old [44]. |
|  | **Comparator details** |  |
| Outcome measures | Confirmed COVID-19 cases | Persons with SARS-CoV-2 infection confirmed with reverse transcriptase–polymerase chain reaction (RT-PCR) and received standard isolation treatment. All confirmed COVID-19 cases, contacts, and individuals in Saudi Arabia who were considered to be at high risk and were registered at hospitals from 1February to 27 July 2020. |
|  | Suspected individuals | Known as any person who had direct contact with a person infected with SARS-CoV-2, had to offer a throat swab sample to test for the presence of SARS-CoV-2. This test was performed when the person was detected by the Novel Saudi Arabia Ministry of Health software applications “Tabaoud” and “Tawakkalna”. In the case the RT-PCR results were positive for SARS-CoV-2, persons were obliged to stay in the hospital, if not, individuals were isolated at a hotel under the control and care of physicians and nurses. |
|  | Deaths |  |
|  | R0 | Defined as the mean figure of secondary cases created by primary cases as people are generally vulnerable to infection, defines the overall number of people expected to be infected, or more accurately, the area below the outbreak curve |
| Source of data | - Population size and age distributions among the country of Saudi Arabia were taken from the 2020 World Population Prospects, the 27th round of the official United Nations population estimations organized by the Population Division of the Department of Economic and Social Affairs of the United Nations Secretariat. - The patterns of contact among Saudi populations in deferent provinces were taken from several sources including the Colliers report and The Pulse: 8th edition, Kingdom of Saudi Arabia Healthcare overview 2018. - Additional data were obtained from a survey of socioeconomic levels of different countries included in the socialmixR packages. - Finally, contact patterns were measured using the Saudi Arabia Ministry of Health Statistical Annual Report. | |
| Data analyiss | - Group data analysis was compared by using one-way variance (ANOVA), and the differences between the means of two of the three studied groups were analysed using an independent-sample t-test. - The significance level was p < 0.05. SPSS software (SPSS Inc.; Chicago, IL, USA) was used for the statistical analyses. | |
| Results | | |
| Study parameters | Statistical analysis data are shown as mean standard deviation (SD) for the various studied parameters and their correlations. | |
| outcomes (Mean values for the main categories of estimated outcomes of interest, as well as mean differences between the comparator groups.) | - In Saudi Arabia, the current rate of transmission (R0) was estimated to be around 4 at the beginning of the pandemic in Saudi Arabia in March 2020. Then, the R0 decreased to be around 2–3 through March to June. At the beginning of July, the R0 was lowered to reach 1 around all Saudi cities. The decrease in R0 around Saudi cities is related to the country lockdown and government prevention measures. - Performing preventive measures in reducing the infection and death rates around Saudi Arabia by 27%. | |
| Characterising heterogeneity |  | |
|  | | |
| Discussion | | |
| Study findings, (Summarise key study findings and describe how they support the conclusions reached.) | - The current rate of transmission (R0) was estimated to be around 4 at the beginning of the pandemic in Saudi Arabia in March 2020. Then, the R0 decreased to be around 2–3 through March to June. At the beginning of July, the R0 was lowered to reach 1 around all Saudi cities. The global rate of transmission R0 ranged from 2.5–3.5. The decrease in R0 around Saudi cities is related to the country lockdown and government prevention measures. - It was noticeable that most preventive measures taken by the government were implemented by March 15, meaning that only 300 cases from a 35 million population of about 0.03% per million. - By 21 July, the confirmed new cases per million of Saudi population reached 7667,663 per million with the banning of international flights remaining in place and prohibitions of the Umrah and Hajj. | |
| limitations | All reported cases were analyzed in consideration of the announced numbers by the Ministry of Health ignoring lost and unknown cases as well as the comparatively short duration of follow-up, the outcomes related with the interference measures depicted in this study should be evaluated with concern. | |
| generalisability | Due to the above-mentioned limitation more forthcoming cohort studies about community control strategies should be performed in the future including more and larger cases. Thus, community control policies may need adjustments for effective application. | |
| current knowledge (how the findings fit with current knowledge.) | The certainty of the actual total number of infected people around the world is still unconfirmed. The only way to know a country’s total number is through the number of tests performed on suspected people around the country. This signifies that the number of confirmed cases vary in the country’s tests performed. Testing is the significant player in reducing the pandemic and its spread across the country. | |
| changes in diagnostic tests and access and any adjustment | On 27 July, 2020, the Saudi Arabia Ministry of Health declared that about three million COVID-19 tests had been conducted since the beginning of the pandemic, and the total number of coronavirus cases reached 262,772, of which 2378 were new cases. The rise in confirmed cases took place as the daily tests at all healthcare facilities in the kingdom increased from 1000 to 65,000 tests daily. | |

1. **Effect of Social Distancing on COVID-19 Incidence and Mortality in Iran Since February 20 to May 13, 2020: An Interrupted Time Series Analysis (Alimohamadi et al., 2020)**

| Data Collection Item | Item description / sub-groups | |
| --- | --- | --- |
| Bibiliographic Information | | |
| Author | Alimohamadi et al. | |
| Title | Effect of Social Distancing on COVID-19 Incidence and Mortality in Iran Since February 20 to May 13, 2020: An Interrupted Time Series Analysis | |
| Year of publication | 2020 | |
| Country | Iran | |
|  | | |
| Methods | | |
| Type of Study | Quasi-experimental study, Interrupted Time Series Analysis | |
| Time period and setting of the study | Iran; From Feb 20, 2020, to May 13, 2020.   - Finding on the incidence of COVID-19 in the Princess Diamond cruise ship shows that deaths began two weeks after the identification of the Index case. Thus, it is reasonable that one examines the effects of social distancing on mortality and morbidity rates after two-week of mentation. So, deaths and cases occurred before April 9, 2020, were considered as a baseline (control period), while death and cases occurred after April 9, 2020, as an intervention period. | |
| Intervention | social distancing | |
| description of intervention | - Close down shopping malls and big markets, sports places, and swimming pools, parks, and museums and even mosques, holy shrines, and Friday prayers. - April 9, 2020, is considered as the starting time of social distancing (distancing started formally in the fourth week of March 2020). | |
| setting of the intervention | Community | |
| Aim/ Objectives | To assess the effectiveness of social distancing measures on the COVID-19 incidence and mortality in Iran. | |
| Participant characteristics | Participants | A total population of more than 83 million |
|  | Comparator details |  |
| Outcome measures | Daily incidence cases | per 100,000 population |
|  | The number of deaths of COVID | per 100,000 population |
| Source of data | World in Data website that is available at https://ourworldindata.org/coronavirus. | |
| Data analyiss | - Segmented regression model and Interrupted time series (ITS) analysis using Newey ordinary least squares Newey ordinary least squares (OLS) regression-based methods were used to model the under-studied data. - Data included 84 observations of the deaths and COVID-19 confirmed cases that occurred in Iran during the mentioned period. - Data were analysed using StataCorp. 2017. Stata Statistical Software: Release 15. College Station, TX: StataCorp LLC (USA). | |
|  | | |
| Results | | |
| Study parameters | Daily incidence cases per 100,000 population;  The number of deaths of COVID per 100,000 population | |
| outcomes (Mean values for the main categories of estimated outcomes of interest, as well as mean differences between the comparator groups.) | - The starting point of the new cases of COVID-19 was estimated at −0.38 (Intercept), and the trend of new cases increase significantly every day until 9th April 2020 according to pre-intervention slope of (ß=0.08 (95% CI = [0.06–0.10]; P < 0.001)). - After the intervention (social distancing) implementation the occurrence of new cases was significantly decreasing (ß = −1.70 (95% CI = [−2.30 – −1.10; P < 0.001])). - The occurrence of new cases had a decreasing trend over the time after 9 April 2020 with a coefficient of −0.10 (95% CI = [−0.11– −0.07; P < 0.001]) - The trend of proportion of COVID-19 deaths was significantly increasing before the implementation of social distancing (9 April 2020) (ß = 0.005 (95% CI = [0.004–0.006]; P < 0.001)). - The trend of COVID-19 deaths was significantly decreasing after the implementation of social distancing (ß = −0.07 (95% CI = [−0.10 – −0.05; P < 0.001])). - The trend of new deaths of COVID 19 was increasing before the implementation of the intervention (9 April 2020), but it was decreasing after the implementation of the intervention. | |
| Characterising heterogeneity | - | |
|  | | |
| Discussion | | |
| Study findings, (Summarise key study findings and describe how they support the conclusions reached.) | - The results of the present study showed that social distancing significantly reduced the incidence and mortality of COVID-19 in Iran. - Despite the increase in the number of cases before and the early days of social distancing implementation, there was a significant reduction after the implementation. However, a reduction in the occurrence of new COVID-19 cases since April 9, 2020 (after the implementation of social distancing) was steady. This finding is consistent with the incubation period of the infection. - The present study suggests that social distancing could decrease the incidence and mortality related to COVID-19. Thus, it is necessary to continue these measures, because if the government plan to reduce the social distancing measures the occurrence of the next waves of the epidemic will be inevitable. | |
| limitations | - First, during the study period, some variables such as the definition of the case and accuracy of the diagnostic tests may be changed and this could affect the effectiveness of the intervention positively or negatively. - Second, some variables such as the level of the population knowledge regarding COVID-19, the extent to which people have access to the health-care system, and their compliance with health principles could affect the effectiveness of social distancing. However, since data on these variables were not available, controlling their role was not possible. - Third, unregistered infected individuals are likely to maintain the disease transmission cycle in families 21,20 which obscure the effectiveness of social distancing. | |
| generalisability |  | |
| current knowledge (how the findings fit with current knowledge.) | An average estimated incubation period of COVID-19 is 5.1 days, and in most cases, the disease detected around the second week of the incubation period. Thus, some cases of the disease might have been infected in the days before the implementation of social distancing, and diagnosed after the implementation of the measure. Therefore, a significant reduction in the incidence of the disease was observed in 1–2 weeks after the implementation of social distancing. | |
| changes in diagnostic tests and access and any adjustment | During the study period, some variables such as the definition of the case and accuracy of the diagnostic tests may be changed and this could affect the effectiveness of the intervention positively or negatively. | |

1. **Association Between Statewide School Closure and COVID-19 Incidence and Mortality in the US** **(Auger et al., 2020)**

| Data Collection Item | Item description / sub-groups | |
| --- | --- | --- |
| Bibiliographic Information | | |
| Author | Auger et al. | |
| Title | Association Between Statewide School Closure and COVID-19 Incidence and Mortality in the US | |
| Year of publication | 2020 | |
| Country | USA | |
|  | | |
| Methods | | |
| Type of Study | population–based observational study conducted using interrupted time series analyses | |
| Time period and setting of the study | - All 50 US states Between March 9, 2020, and May 7, 2020. This period allowed for at least 6 weeks of data collection after school closures in each state. - Acknowledging that school closure and other non-pharmaceutical interventions would not have immediate effects on COVID-19 incidence and mortality, estimates were used to determine when school-based exposure could be expected to lead to changes in COVID-19 incidence and associated mortality. A time from exposure to symptom onset of 5 days was assumed per Lauer et al. Given the early emphasis (and some state restrictions) on limiting testing to hospitalized patients, time to diagnosis was defined as time between symptom onset and hospitalization (7 days). For school closure, given the low documented prevalence of COVID-19 in children, an additional period was included for a child to infect an adult, assuming a child exposed at school could expose an adult prior to symptom onset and within 4 days. The analyses for the mortality outcome assumed 17 days from symptom onset to death. | |
| Intervention | Closure of primary and secondary schools (kindergarten-grade 12). | |
| description of intervention | All 50 states closed schools in March 2020 | |
| setting of the intervention | Schools in the USA | |
| Aim/ Objectives | To determine if school closure and its timing were associated with decreased COVID-19 incidence and mortality. | |
| Participant characteristics | Participants | All 50 states of USA with 322.2 million residents |
|  | Comparator details |  |
| Outcome measures | Daily COVID-19 incidence | Per 100000 residents in each state. In accordance with CDC guidelines, confirmed COVID-19 cases include presumptive positive cases and probable cases, and death totals include confirmed and probable cases. |
|  | Daily COVID-19 mortality | per 100,000 residents in each state |
|  | Cumulative incidence of COVID-19 | Defined as total number of cases per 100000 residents |
| Source of data | Outcome measures derived from publicly available data from the Johns Hopkins University School of Public Health, which aggregates data from the US Centers for Disease Control and Prevention (CDC) as well as from state and local public health departments. | |
| Data analyiss | - Interrupted time series analyses were used to compare the daily change in outcomes (daily COVID-19 incidence and mortality) before and after school closure. - Analyses were performed using SAS version 9.4 (SAS Institute Inc) and 2-sided P values of <.05 were considered statistically significant. | |
|  | | |
| Results | | |
| Study parameters | Daily COVID-19 mortality per 100,000 residents in each state;  Cumulative incidence of COVID-19 Defined as total number of cases per 100000 residents;  The denominator for the outcome measures was the state population from the 2018 American Community Survey. | |
| outcomes (Mean values for the main categories of estimated outcomes of interest, as well as mean differences between the comparator groups.) | - School closure was associated with a significant decline in the incidence of COVID-19 (adjusted relative change per week, −62% [95%CI,−71% to −49%]) and mortality (adjusted relative change per week, −58%[95%CI, −68%to−46%]). - Both of these associations were largest in states with low cumulative incidence of COVID-19 at the time of school closure. For example, states with the lowest incidence of COVID-19 had a −72% (95% CI, −79% to −62%) relative change in incidence compared with −49% (95% CI, −62%to −33%) for those states with the highest cumulative incidence. - In a model derived from this analysis, it was estimated that closing schools when the cumulative incidence of COVID-19 was in the lowest quartile compared with the highest quartile was associated with 128.7 fewer cases per 100 000 population over 26 days and with 1.5 fewer deaths per 100 000 population over 16 days. | |
| Characterising heterogeneity | - States were examined in quartiles based on state-level COVID-19 cumulative incidence per 100 000 residents at the time of school closure. - For each state, the following non–school-related non-pharmaceutical intervention covariates were considered: stay-at-home or shelter-in-place order, nonessential business closure, restaurant and bar closure, and prohibition of gatherings with more than 10 people. These non-pharmaceutical interventions were included based on the policy effective date plus a lag period to allow for any potential policy-related effects on daily COVID-19 incidence and mortality. | |
|  | | |
| Discussion | | |
| Study findings, (Summarise key study findings and describe how they support the conclusions reached.) | - Between March 9, 2020, and May 7, 2020, school closure in the US was temporally associated with decreased COVID-19 incidence and mortality; states that closed schools earlier, when cumulative incidence of COVID-19 was low, had the largest relative reduction in incidence and mortality. However, it remains possible that some of the reduction may have been related to other concurrent non-pharmaceutical interventions. - In this study, changes in COVID-19 incidence and mortality associated with school closure were isolated to the extent possible by adjusting for other state-enacted policies and testing rates. In adjusted models, school closure was associated with decreased COVID-19 incidence and deaths. These analyses do not incorporate the risks of school closure on child education and development or from a societal perspective. However, the analyses suggest that school closure may be effective in curbing SARS-CoV-2 spread and preventing deaths during future outbreaks. - School closure in this study was associated with a −62% relative change in COVID-19 incidence per week. A decline of 62% was equivalent to 39% of the projected value with schools open. | |
| limitations | - First, many states enacted additional non-pharmaceutical interventions concurrently with or shortly after school closure, making it impossible to fully isolate potential effects of school closure. - Second, analyses were conducted at the state level. The analyses did not account for resident travel leading to viral spread between states. Even though the study modeled state level policies, some states had more restrictive policies locally (ie, by county). - Third, inadequate testing has impeded COVID-19 diagnosis. Testing variability was accounted for with the use of state level testing rates as a model covariate; however, testing rates do not fully capture a state’s testing capability, infrastructure, and strictness of testing guidelines. - Fourth, the completeness and accuracy of the Johns Hopkins University database with respect to COVID-19 incidence and mortality has not been established. | |
| generalisability | This study has included all 50 states of USA in the analysis. However, due to the context-based feature of the pandemic it is not easy to judge about the generalizability of the study to other countries. | |
| current knowledge (how the findings fit with current knowledge.) | - As hypothesized, school closure in states that enacted this intervention early (when the cumulative incidence ofCOVID-19waslow) had greater associated relative decreases in incidence and mortality. Although these relative differences translate into smaller absolute differences associated with school closure, states that closed schools later (in the highest quartile ofCOVID-19 cumulative incidence) had more new cases and deaths from COVID-19 during the period after school closure. Thus, this study can inform future decisions about optimal timing for state and local officials to consider school closure to curb SARS-CoV-2 spread in the high likelihood that the pandemic continues. - The mechanism by which school closure could affect COVID-19 spread is not only through disrupting spread by or among children. School closure affects family routines, necessitating alternative childcare and modified work schedules. - It is unclear how COVID-19 spread would be affected if schools remained open while states enacted other policies to restrict movement. | |
| changes in diagnostic tests and access and any adjustment | - Measured COVID-19 incidence was affected by testing availability, which was limited early in the pandemic and varied nationally. - SARS-CoV-2 testing rates varied by state and throughout the study period. To account for this variation, state-level COVID-19 testing (calculated daily as the cumulative number of tests per 1000 residents) was modelled as a categorical variable. | |

1. **The effect of lockdown on the outcomes of COVID-19 in Spain: An ecological study (Camila Alves dos Santos et al., 2020)**

| Data Collection Item | Item description / sub-groups | |
| --- | --- | --- |
| Bibiliographic Information | | |
| Author | Camila Alves dos Santos Siqueira et al. | |
| Title | The effect of lockdown on the outcomes of COVID-19 in Spain: An ecological study | |
| Year of publication | 2020 | |
| Country | Spain | |
|  | | |
| Methods | | |
| Type of Study | Ecological study | |
| Time period and setting of the study | Spain states; March 14 - April 25, 2020.   - These dates corresponded to when the State of Alarm was declared by the government (which instituted the lockdown on March 15), and the day before the lockdown was eased (i.e., children allowed out if accompanied by a single adult), respectively. | |
| Intervention | Lockdown | |
| description of intervention | Physical distancing measures enforced by the autonomous communities of Spain. Lockdown was declared on March 14, 2020. | |
| setting of the intervention | Spanish autonomous community | |
| Aim/ Objectives | To investigate the impact of physical distancing measures enforced by the autonomous communities of Spain, regarding incident cases, hospitalizations (general hospital ward and Intensive Care Unit ward), and mortality trends related to COVID-19. | |
| Participant characteristics | Intervention group | A total population of 47,026,208;  During the period encompassed by this study (March 14 –April 25, 2020), 223,791 new cases of COVID-19 were registered in Spain, along with 23,135 deaths. |
|  | Comparator details |  |
| Outcome measures | Hospital admissions | individuals admitted to general hospital wards |
|  | ICU admissions | patients that received critical care in ICUs |
|  | Incidence | Incidence rates were calculated for the entire period for new confirmed cases, hospital admissions, ICU admissions, deaths, and recoveries. |
|  | Deaths |  |
|  | Recoveries |  |
| Source of data | - Daily data was published by the Health Ministry of Spain, per autonomous community, on the number of new cases, hospital admissions, Intensive Care Unit (ICU) admissions, deaths, and recoveries. - Autonomous community population data were obtained from Health Ministry of Spain and Spanish National Statistics Institute for 2019. (publicly available) | |
| Data analyiss | - Information on new cases, hospital admissions, ICU admissions, deaths, and recoveries were analyzed by the Joinpoint Regression Program, version 4.8.0.1. - Stratified data analysis was carried out for each autonomous community. However, it was detected that some data did not follow the same pattern of accumulated data of the other communities. This was the case of Madrid and Castile-La Mancha, which precluded trend analysis for hospital and ICU admissions, and of Castile and Leo´n and Galicia, regarding ICU admissions. - For each segment, the Daily Percentage Change (DPC) was calculated to identify the statistical significance (p-value<0.05), with a 95% confidence level. | |
|  | | |
| Results | | |
| Study parameters | Percentage of change per time interval;  The analysis considered 43 days of monitoring;  The temporal unit employed herein was a day. | |
| outcomes (Mean values for the main categories of estimated outcomes of interest, as well as mean differences between the comparator groups.) | - The day when the trend changed varied across autonomous communities and evaluated indicators. - Regarding the mean number of days elapsed for a change to occur in the evolution pattern of the disease and the beginning of the inflection of the curve, the mortality rate presented the latest joinpoint (mean = 18.33 days, standard deviation—SD = 5.37, median = 18.50 and interquartile range–IQR = 6). The following parameters were general hospital ward admissions (mean = 14.27 days, SD = 4.68, median = 14.00 and IQR = 2.5), ICU admissions (mean = 13.44 days, SD = 4.42, median = 13.00 and IQR = 5), and finally, incidences (mean = 12.18 days, SD = 2.92, median = 12.00 and IQR = 1.5). - Change in trends, with a consequent reduction in the number of deaths, took longer than the national average for Navarre (34 days), Basque Country (25 days), Extremadura (24 days), Murcia (22 days), La Rioja (24 days), Ceuta (23 days), Andalusia (21 days), and Valencia and Balearic Islands (19 days each). - Regarding hospital and ICU admissions, the highest rates also occurred in the regions with a higher number of cases: La Rioja is highlighted as the community with the highest hospital admission rates, while Catalonia presented the highest ICU admission rates. | |
| Characterising heterogeneity | Not mentioned. Only data were analysed based on per Spanish autonomous community. | |
|  | | |
| Discussion | | |
| Study findings, (Summarise key study findings and describe how they support the conclusions reached.) | - Analysis of mortality trends, confirmed cases, hospital admissions, and ICU admissions revealed an increasing pattern, followed by a reduction, for all regions with registries for these data. Ceuta was an exception, where stability was observed for the rates associated with hospital and ICU admissions. In Melilla, stable rates were identified for hospital admissions, and decreasing rates were obtained for ICU admissions. - The results of the COVID-19 data analysis in Spain demonstrate the positive impact of the lockdown in containing the disease. It was possible to identify a similar pattern in the majority of autonomous communities in Spain, characterized by a pronounced decline in incidence, hospital admissions, ICU admissions, and mortality rates. - The best indicator for the evaluation of the consequences of the pandemic was the mortality rate, which presented the highest uniformity across registries, besides representing the worst outcome of the disease. The assessment of these trends is a vital instrument to substantiate decision-making. | |
| limitations | - The constant review of data must be mentioned in the pandemic context, with the possibilities of delayed notifications according to the information made available by each autonomous community. An eventual accumulation of data for subsequent publication would hinder the evaluation of daily trends, due to the probabilities of undernotification. - Also, it is essential to remark that the majority of diagnoses in Spain followed PCR tests. Therefore, despite its limitations, the epidemiological importance of this study is undeniable. | |
| generalisability | One of the strengths of this study is the comparison of populations in different moments of the pandemic, with varying rates of incidence, but submitted to the same public health measures during the same length of time. Therefore, the findings herein presented can subsidize the decision-making process in different international contexts. | |
| current knowledge (how the findings fit with current knowledge.) | - There was a high number of deaths in nursing homes in Spain. The institutionalized population must be a priority for preventive actions due to their vulnerability to respiratory diseases and the coexistence of several people in small, common spaces. - Data published by the Spanish Ministry of Health does not specify how the recovered cases are accounted for. This indicator is reported in almost all COVID-19 statistics but must be standardized to enable comparison across different territories. - Besides the time required for a change in trends, is the speed of increase of all aforementioned indicators, even after the lockdown was declared throughout Spain. Two communities that experienced early lockdowns, enforced at the early stages of the pandemic, visibly mitigated consequences. In the cases of these two regions, it would probably not be necessary to institute such a long lockdown time. - The highest accumulated incidence trends were detected in the autonomous communities that concentrate the most important urban centers and present the highest GDP per inhabitant in Spain, with more developed transportation systems. These characteristics probably have helped spread the SARS-CoV-2. - Even with the enforcement of the lockdown, some communities experienced increases in daily mortality rates over 20%. The higher rates in these communities are possibly associated with the higher demographic density of the most affected cities and higher mobility before the lockdown. The communities with the lowest quantity of cases, in contrast, practically did not suffer increases in mortality, emphasizing the benefits of early lockdowns. | |
| changes in diagnostic tests and access and any adjustment | - | |

1. **Examining the effect of social distancing on the compound growth rate of COVID-19 at the county level (United States) using statistical analyses and a random forest machine learning model(Cobb & Seale, 2020)**

| Data Collection Item | Item description / sub-groups | |
| --- | --- | --- |
| Bibiliographic Information | | |
| Author | Cobb et al. | |
| Title | Examining the effect of social distancing on the compound growth rate of COVID-19 at the county level (United States) using statistical analyses and a random forest machine learning model | |
| Year of publication | 2020 | |
| Country | USA | |
|  | | |
| Methods | | |
| Type of Study | Prospective cohort study | |
| Time period and setting of the study | From January 21, 2020, to March 31, 2020, in all 3139 US counties | |
| Intervention | Social distancing; county-level shelter-in-place (SIP) | |
| description of intervention | - Instituting a form of ‘social distancing’ by limiting gatherings to 10 or fewer people. - Issued on March 16, 2020 | |
| setting of the intervention | Community of United States | |
| Aim/ Objectives | To investigate trends among US counties and coronavirus disease 2019 (COVID-19) growth rates in relation to the existence of shelter-in-place (SIP) orders in that county. | |
| Participant characteristics | Participants | Counties that had confirmed cases of COVID-19 before issue of guidelines on March 16 and experienced a SIP order on or after March 19 (186 counties, referred to as wSIP); |
|  | Comparator details | Counties that had confirmed cases before March 16 and experienced no SIP order (60 counties, referred to as noSIP). |
| Outcome measures | Compound growth | - Compound growth rates were calculated using cumulative confirmed COVID-19 cases. - Compound growth was calculated using the following equation: (final confirmed cases/first confirmed cases)^(1/number of days). - Compound growth was chosen as it gives a single number that can be used in machine learning to represent the speed of virus spread during defined time intervals. |
| Source of data | - County metrics were obtained from the US Census Bureau, USA Counties (2011) data sets from the 2010 census. - The number of confirmed COVID-19 cases in each county was collected from local health department data and county/state press releases from January 21 to March 25. - Confirmed cases from March 26 to 31 were obtained from The New York Times coronavirus data repository. The two data sets were compared to ensure consistency between the collected values. Data collection stopped on March 31 because the mean number of days with confirmed cases was approximately the same as that before issue of guidelines on March 16, after March 16 but before the institution of a SIP order, and after March 16 with a SIP order. This allowed for comparison of these time intervals with an equal number of days (7.62 ± 0.35). | |
| Data analyiss | - A Student t-test was used to compare two groups for significance. - Analysis of variance with the Tukey post hoc test was used to compare multiple groups. - Significance was defined as P <0.05. All data are reported as mean ± 95% confidence interval. | |
|  | | |
| Results | | |
| Study parameters | Mean compound growth rate of counties; | |
| outcomes (Mean values for the main categories of estimated outcomes of interest, as well as mean differences between the comparator groups.) | - The compound growth rate for the wSIP (1.39 ± 0.044) and noSIP (1.30 ± 0.059) groups before the issue of presidential guidelines on March 16, the compound growth rate after March 16 for the wSIP (1.30 ± 0.023) and noSIP (1.21 ± 0.016) groups, and the compound growth rate for the wSIP group (1.19 ± 0.011) after the SIP orders went into effect. - The lower compound growth rates seen in noSIP data are due to the difference in latitude between the two data sets and suggest that southern states experienced a slower spread of the virus at the onset. This makes sense, given that before March 16, the hot spots for COVID-19 were northern states such as Washington, New York, and Illinois. The noSIP compound growth rates were normalized to the compound growth rate of wSIP data before March 16 to account for geographical differences. The normalized compound growth rates after March 16 were shown to be statistically similar between the wSIP and noSIP groups (P > 0.05). This indicates that the presidential guidelines had the same magnitude of effect on reducing the compound growth rate by 6.6 ± 1.4% between the wSIP and noSIP groups before the wSIP group instituted a SIP. - After instituting a SIP order, compound growth rate of the wSIP group decreased an additional 7.8%, for a total decrease of 14.4 ± 1.6% from the compound growth rates before March 16. This indicates that the effects from the presidential guidelines and SIP orders were additive in the US. This is reasonable, considering the virus is thought to spread by virus containing airborne droplets and orders for social distancing limit the interaction of people who could potentially be infected. | |
| Characterising heterogeneity | - There were no statistically significant differences in the US census data between the wSIP and noSIP groups apart from latitude (P < 0.0001) and the number of physicians (P = 0.04). - The wSIP group had a latitude of 39.47 ± 0.75, which places it in the northern US, compared with the noSIP group with a latitude of 34.6 ± 1.16°, placing it further south. The difference in number of physicians is a function of latitude with a lower mean number of physicians in the south (1697 ± 500) compared with the north (2677 ± 538). | |
|  | | |
| Discussion | | |
| Study findings, (Summarise key study findings and describe how they support the conclusions reached.) | - The data suggest that at a county level, in the US, the SIP order is effective at decreasing the compound growth rate of COVID-19. - The counties that have the largest impact from a SIP order are ones with a large population or a high population density, as indicated by the random forest feature importance. | |
| limitations | Not explicitly mentioned | |
| generalisability |  | |
| current knowledge (how the findings fit with current knowledge.) | SIP orders were found to be effective at reducing the growth rate of COVID-19 cases in the US. Counties with a large population or a high population density were found to benefit the most from a SIP order. | |
| changes in diagnostic tests and access and any adjustment | - | |

1. **Strong Social Distancing Measures In The United States Reduced The COVID-19 Growth Rate (Courtemanche et al., 2020)**

| Data Collection Item | Item description / sub-groups | |
| --- | --- | --- |
| Bibiliographic Information | | |
| Author | Courtemanche et al. | |
| Title | Strong Social Distancing Measures In The United States Reduced The COVID-19 Growth Rate | |
| Year of publication | 2020 | |
| Country | United States (USA) | |
|  | | |
| Methods | | |
| Type of Study | Event study design | |
| Time period and setting of the study | US counties; between March 1, 2020, and April 27, 2020.   - March 1 was chosen as the start date because no new cases were reported in the entire US on most days in January and February. - The April 27 was chosen as end date to coincide with the first removal of one of the four types of restrictions analyzed (the reopening of restaurants and other entertainment facilities in Georgia). - Each county observation was weighted by population, using 2018 estimates from the Department of Agriculture’s Economic Research Service. | |
| Intervention | Government-imposed social distancing measures | |
| description of intervention | - Bans on large social gatherings; school closures; closures of entertainment venues, gyms, bars, and restaurant dining areas; and shelter-in-place orders. - Shelter-in-place orders were generally the last policy to be implemented, and adoption of them was uniformly lower than for the other policies. - On March 1 no jurisdiction had implemented all four measures. - By March 22 nearly 25 percent of the US population was covered by all of the measures. This rose to approximately 65 percent by March 29 and 95 percent by April 7, when the last shelter-in-place order took effect. | |
| setting of the intervention | Community; school; public places at USA | |
| Aim/ Objectives | To evaluate the impact of these measures on the growth rate of confirmed COVID-19 cases across US counties | |
| Participant characteristics | 3,142 US counties (official COVID-19 records report New York City as a whole instead of dividing it into five counties, reducing this number to 3,138), leading to a sample size of 182,004 observations. | |
|  |  | |
| Outcome measures | Growth rate | The daily exponential growth rate was calculated as the natural log of cumulative daily COVID-19 cases minus the log of cumulative daily COVID-19 cases on the prior day. |
|  |  |  |
| Source of data | COVID-19 Dashboard provided by the Johns Hopkins Center for Systems Science and Engineering. This repository contains data on COVID-19 cases worldwide, collected from a range of sources including government and independent health institutions. | |
| Data analyiss | - The relationship between social distancing policies and the exponential growth rate of confirmed COVID-19 cases was estimated using an event study regression with multiple policies. - Statistical analysis was conducted using Stata MP, version 15. This approach is akin to difference-in-differences but is more flexible, as it interacts the policy variables with multiple indicators of time since implementation, thereby tracing out the evolution of the policy effects over time. | |
|  | | |
| Results | | |
| Study parameters | The unit of observation was daily US counties or county equivalents;  Daily growth rate in confirmed COVID-19 cases at the county level. | |
| outcomes (Mean values for the main categories of estimated outcomes of interest, as well as mean differences between the comparator groups.) | - Relative to the reference category of zero to four days before implementation, shelter-in-place orders led to statistically significant (p < 0:01) reductions in the COVID- 19 case growth rate of 3.0 percentage points after six to ten days, 4.5 percentage points after eleven to fifteen days, 5.9 percentage points after sixteen to twenty days, and 8.6 percentage points from twenty-one days onward. Because the model held constant the other types of policies, these estimates should be interpreted as the additional effect of shelter-in-place orders beyond the effects of shutting down schools, large social gatherings, and entertainment-related businesses. This additional effect may come either from the requirement or strong advisement to shelter in place aside from essential activities or from the accompanying closure of any nonessential businesses that remained open. We did not observe any statistically significant placebo effects of shelter-in-place orders in the periods before implementation, which gives credence to a causal interpretation of our main results. - Investigators found no evidence that bans on large social gatherings influenced the growth rate of confirmed COVID-19 cases. The point estimates for banning gatherings were statistically insignificant (p > 0:56 in all cases). However, the 95% confidence intervals included reductions of up to 3–6 percentage points, so the lack of evidence of an effect should not be misinterpreted as clear evidence of no effect. - Closing restaurant dining rooms and bars or entertainment centers and gyms led to significant reductions in the growth rate of COVID-19 cases in all periods after implementation (p < 0:05). The estimated effect was 4.4 percentage points after one to five days, 4.7 percentage points after six to ten days, 6.1 percentage points after eleven to fifteen days, 5.6 percentage points after sixteen to twenty days, and 5.2 percentage points after twenty-one days or longer. Before implementation, policies related to businesses showed no effect on the growth rate, again passing the placebo test. - Investigators found no evidence that school closures influenced the growth rate in confirmed COVID-19 cases. The point estimates were never close to statistically significant (p > 0:37 in all cases), but the 95% confidence intervals meant that we could not rule out reductions of up to 4–5 percentage points. - Adding the coefficient estimates for each policy gives the combined effect of implementing all four social distancing policies. In the first one to five days after implementation, the bundle of restrictions reduced the growth rate of COVID-19 cases by 5.4 percentage points. This reduction grew to 6.8 percentage points after six to ten days, 8.2 percentage points after eleven to fifteen days, 9.1 percentage points after sixteen to twenty days, and 12.0 percentage points after twenty one days or more. | |
| Characterising heterogeneity | - | |
|  | | |
| Discussion | | |
| Study findings, (Summarise key study findings and describe how they support the conclusions reached.) | - Adoption of government-imposed social distancing measures reduced the daily growth rate of confirmed COVID-19 cases by 5.4 percentage points after one to five days, 6.8 percentage points after six to ten days, 8.2 percentage points after eleven to fifteen days, and 9.1 percentage points after sixteen to twenty days. - Holding the amount of voluntary social distancing constant, these results imply that there would have been ten times greater spread of COVID-19 by April 27 without shelter-in-place orders (ten million cases) and more than thirty five times greater spread without any of the four measures (thirty-five million cases). | |
| limitations | - Official COVID-19 case counts are known to understate the true prevalence of the disease, as they do not include asymptomatic carriers. Nonetheless, confirmed case counts are crucial to the Trump administration’s “Opening Up America Again” plan, which proposes either a “downward trajectory of documented cases within a 14-day period” or a “downward trajectory of positive tests as a percent of total tests within a 14-day period (flat or increasing volume of tests)” as criteria for loosening social distancing measures. - Moreover, to the extent that testing shortages led to only the sickest individuals receiving tests, official case counts can loosely be interpreted as the prevalence of moderate to- severe illness, a relevant metric for policy purposes. Available data allowed the control for the number of tests performed at only the state, rather than county, level. However, most of the policy variation is at the state level, so controlling for state-level testing should go a long way toward alleviating bias. | |
| generalisability |  | |
| current knowledge (how the findings fit with current knowledge.) | Although the results suggest that both shelter in- place orders and other measures can be effective at averting COVID-19 cases, the lack of evidence of effects of school closures or bans on large social gatherings is noteworthy. We cannot rule out the possibility that these null results are a result of statistical imprecision, but it is also possible that both policies may displace social interaction instead of reducing it. | |
| changes in diagnostic tests and access and any adjustment | Control variables related to the availability of COVID-19 tests were included. | |

1. **Lockdown Contained the Spread of 2019 Novel Coronavirus Disease in Huangshi City, China: Early Epidemiological Findings (Ji et al., 2020)**

| Data Collection Item | Item description / sub-groups | |
| --- | --- | --- |
| Bibiliographic Information | | |
| Author | Ji et al. | |
| Title | Lockdown Contained the Spread of 2019 Novel Coronavirus Disease in Huangshi City, China: Early Epidemiological Findings | |
| Year of publication | 2020 | |
| Country | China | |
|  | | |
| Methods | | |
| Type of Study | Observational | |
| Time period and setting of the study | Huangshi City. From 10 January to 27 March 2020 | |
| Intervention | Sealed Wuhan (quarantine) | |
| description of intervention | For two months (between 24 January and 23 March 2020)   - As of 10:00 AM Beijing time, 23 January 2020, Wuhan shut down all public transportation, including the airport, railway station, and bus and subway services. - Closure of travel to and from the Huangshi was implemented starting 10:00 AM, 24 January 2020. In addition, Huangshi on 25 January 2020 addressed a wide range of nonpharmaceutical interventions (NPI) regarding surveillance, detection, and containment measures. - First, real-time syndromic surveillance was used for individuals seeking care at clinics or hospitals to detect the onset of the illness. Second, health screening and quarantine were applied for incoming travelers. Third, voluntary personal social isolation (such as staying at home) and compulsory facial masks outdoors were indicated for all residents, followed by monitoring and reporting. Finally, factories, businesses, schools, and other institutions were closed during the outbreak. The community workers, together with volunteers, dedicated to ensure | |
| setting of the intervention | The whole community of Wuhan and 13 cities in Huangshi | |
| Aim/ Objectives | To describe the epidemiological characteristics in one of the cities and highlight the effect of current implemented lockdown and non-pharmaceutical interventions. | |
| Participant characteristics | Participants | - 1015 confirmed COVID-19 cases, 93.62% (997) of whom were SARS-CoV-2 RNA positive. |
|  | Comparator details |  |
| Outcome measures | Confirmed COVID-19 Cases | - Defined as both (1) fulfilled 2 clinical criteria plus 1 epidemiological clue, or all 3 clinical criteria, and (2) had the throat-swab specimens tested positive for SARS-CoV-2 using real-time reverse transcription polymerase chain reaction (RT-PCR) assay. - Clinical criteria of manifestations were: (1) fever and/or acute respiratory illness, (2) radiographic evidence of pneumonia, and (3) low or normal white-cell count or low lymphocyte count. - Epidemiological clues were: (1) history of travel to or residence in Wuhan city within 14 days prior to symptom onset; (2) close contact with a confirmed or probable case of COVID- 19 within 14 days prior to symptom onset; (3) close contact with persons who had fever or acute respiratory illness and were from Wuhan city or local community with case-reported, within 14 days prior to symptom onset; (4) a cluster of persons with similar symptoms was identified. The laboratory protocol for SARS-CoV-2 real time RT-PCR assay was described previously. The tests were screened by local CDC and confirmed by Hubei provincial CDC. |
| Source of data | - Data of reported cases in Huangshi and Wuhan were retrieved from publicly available disease databases. - Local epidemiological data on suspected or confirmed cases in Huangshi were collected through field investigation. | |
| Data analyiss | - All continuous variables were analyzed with student t tests and categorical variables with χ 2 or Fisher exact tests. - A P-value of <.05 was considered as the level of significance. - Stata software (version 14.0, StataCorp, USA) was used for statistical analysis. - The maps were performed using ArcGIS software (version 10.6, Esri, USA). | |
|  | | |
| Results | | |
| Study parameters | Accumulated number of confirmed and fatal cases with COVID-19;  New confirmed and fatal cases. | |
| outcomes (Mean values for the main categories of estimated outcomes of interest, as well as mean differences between the comparator groups.) | - The percentages of imported cases in patients with symptom onset before 24 January 2020 and patients onset afterward were 63.07% and 31.94%, respectively (P < .001). - The percentage of uncomplicated illness in cases with symptoms onset on/after 24 January 2020 were significantly higher than that in cases with earlier onset (46.48% vs 23.30%, P < .001). - There was no significant difference between cases with onset before 24 January 2020 and those afterward in terms of age, sex, and fatality rate. | |
| Characterising heterogeneity | - | |
|  | | |
| Discussion | | |
| Study findings, (Summarise key study findings and describe how they support the conclusions reached.) | The relationship detected in the analysis strongly suggests that the aggressive public health intervention resulted in flattened epidemic curves and a trend toward better overall outcomes. | |
| limitations | - Only one city with COVID-19 was included. It would be better to include as many cities as possible in Hubei, and in other provinces in China, to get a more comprehensive insight of COVID-19 epidemic. - Second, more detailed information, such as genomic sequencing outcomes and the overall people’s response to NPI measures, was unavailable at the time of analysis. However, the data in this study permit an early assessment of the epidemiological characteristics of COVID-19 in Huangshi, China. - Finally, underreporting bias is also a natural limitation of this study, as some asymptomatic or mild cases might be missed. | |
| generalisability | Patients in Huangshi showed slightly lower case fatality of 3.74% (38/1015), compared with 5.08% (2538/50006) in Wuhan (P = .055). | |
| current knowledge (how the findings fit with current knowledge.) | - The first case of COVID-19 in Huangshi was observed on 2 January. The onset day of epidemic was 10 January, defined as the first day of ascending phase leading to the peak. Right after 24 January (day 1 of lockdown), the epidemic curve became flattened, suggesting that the lockdown intervention slowed the progression of the epidemic. The first of February 2020 (day 9 of lockdown) was identified as the “turning point” as the epidemic in Huangshi faded soon afterward. There have been no new cases observed since 26 February 2020. - Asymptomatic infections have been identified not only in 41 adults but also in 9 children. They had epidemiological clues for COVID-19 contact, and the throat-swab tested positive for SARS-CoV-2 RNA. After quarantined for at least 14 days, all the persons had no signs of illness. | |
| changes in diagnostic tests and access and any adjustment | - The abrupt increase on 12 February was attributed to the diagnosis criteria modification. - From 12 February 2020, the clinically diagnosed patients in Hubei province (not applicable for other provinces in China) who fulfilled 1 epidemiological clue and had radiographic evidence of pneumonia were classified as confirmed cases, regardless of the PCR results. | |

1. **The effect of human mobility and control measures on the COVID-19 epidemic in China(Kraemer et al., 2020)**

| Data Collection Item | Item description / sub-groups | |
| --- | --- | --- |
| Bibiliographic Information | | |
| Author | Kraemer et al. | |
| Title | The effect of human mobility and control measures on the COVID-19 epidemic in China | |
| Year of publication | 2020 | |
| Country | China | |
|  | | |
| Methods | | |
| Type of Study | Observational | |
| Time period and setting of the study | 1st, 2019 - February 10th, 2020 | |
| Intervention | Travel restrictions | |
| description of intervention | Travel restrictions from Wuhan and large-scale control measures started on 23 January 2020 | |
| setting of the intervention | Community of China | |
| Aim/ Objectives | To understand whether the volume of travel within China could predict the epidemic outside of Wuhan | |
| Participant characteristics | Participants | 31 provinces in China |
|  | Comparator details |  |
| Outcome measures | COVID-19 confirmed case | **From January 18-22:**  Need to satisfy criteria for probable case and have a real-time quantitative polymerase chain reaction (RT-qPCR) positive result from sputum, nasopharyngeal swabs, lower respiratory tract secretions or other sample tissue, or genome sequencing highly similar with known SARS-CoV-2.  **From January 22-23:**  Need to satisfy criteria for probable case and have a RT-qPCR positive result from respiratory or blood samples, or genome sequencing highly similar with known SARS-CoV-2.  **From January 23-27:**  Need to satisfy criteria for probable case and have a RT-qPCR positive result from sputum, nasopharyngeal swabs, lower respiratory tract secretions, or other samples, or genome sequencing highly similar with known SARS-CoV-2.  **From January 27-February 5:**  Need to satisfy criteria for probable case and have a RT-qPCR positive result from respiratory or blood samples, or genome sequencing highly similar with known SARS-CoV-2. Available strains from lab test of respiratory or blood samples. |
| Source of data | - Epidemiological data sources were mainly official reports from provincial, municipal or national health governments. - Real-time human mobility data from Baidu Inc., together with epidemiological data from each province. Two pieces of information are collected. First, we extract a series of migration scale indices for traveling out of Wuhan, from January 1st to February 10, both in 2019 and 2020. Second, we obtain the proportion of human movement from Wuhan were bound for each of 31 provinces in China. | |
| Data analyiss | Mixed effects Poisson GLM to estimate the epidemic doubling time across each province. Poisson and negative binomial models, log-linear regression | |
|  | | |
| Results | | |
| Study parameters | Daily counts of cases;  epidemic doubling time (days (range));  the time from symptom onset to confirmation (days (SD));  the mean incubation period (days (SD));  Age and sex distributions. | |
| outcomes (Mean values for the main categories of estimated outcomes of interest, as well as mean differences between the comparator groups.) | - Among cases reported outside of Hubei province in our dataset, 515 cases were observed with known travel history to Wuhan and a symptom onset date before 31 January 2020, compared with only 39 cases after 31 January 2020, illustrating the effect of travel restrictions. - The magnitude of the early epidemic (total number of cases until 10 February 2020) outside of Wuhan was very well predicted by the volume of human movement out of Wuhan alone (R2 = 0.89 from a log-linear regression using cumulative cases). Therefore, cases exported from Wuhan before the cordon sanitaire appear to have contributed to initiating local chains of transmission, both in neighboring provinces (e.g., Henan) and in more distant provinces (e.g., Guangdong and Zhejiang). Further, the frequency of introductions from Wuhan were also predictive of the size of the early epidemic in other provinces (controlling for population size) and thus the probability of large outbreaks. - After 1 February 2020 (corresponding to one mean + one SD incubation period after the cordon sanitaire and other interventions were implemented), the correlation of daily case counts and human mobility from Wuhan decreased, indicating that variability among locations in daily case counts was better explained by factors unrelated to human mobility, such as local public health response. - All provinces outside of Hubei experienced faster growth rates between 9 January and 22 January 2020, which was the time before travel restrictions and substantial control measures were implemented; this was also apparent from the case counts by province. In the same period, variation in the growth rates is almost entirely explained by human movements from Wuhan, consistent with the theory of infectious disease spread in highly coupled meta populations. After the implementation of drastic control measures across the country, growth rates became negative, indicating that transmission was successfully mitigated. | |
| Characterising heterogeneity | - To investigate meaningful shifts in the epidemiology of the COVID-19 outbreak through time, we examined age and sex data for cases from different periods of the outbreak and from individuals with and without travel from Wuhan. - Using crowdsourced case data, we found that cases with travel history (categories I and III) had similar median ages and sex ratios in both the early and later phases of the outbreak (age 41 versus 42 years; 50% interquartile interval: 32.75 versus 30.75 and 54.25 versus 53.5 years, respectively; P value > 0.1, 1.47 versus 1.45males per female, respectively). - Early cases with no information on travel history (category II) had a median age and sex ratio similar to those with known travel history (age 42 years; 50% interquartile interval: 30.5 to 49.5, P value > 0.1; 1.80males per female). However, the sex ratio of later cases without reported travel history (category IV) shifted to ~1:1 (57 male versus 62 female, Χ2 test, P value < 0.01), as expected under a null hypothesis of equal transmission risk, and the median age in this group increased to 46 (50% interquartile interval: 34.25 to 58, t test: P value < 0.01). We hypothesize that many of the cases with no known travel history in the early phase were indeed travellers who contributed to disseminating SARSCoV-2 outside of Wuhan. - The shift toward more equal sex ratios and older ages in non-travelers after 31 January 2020 confirms the finding that epidemics outside of Wuhan were then driven by local transmission dynamics. The case definition changed to include cases without travel history to Wuhan after 23 January 2020. | |
|  | | |
| Discussion | | |
| Study findings, (Summarise key study findings and describe how they support the conclusions reached.) | Early on, the spatial distribution of COVID-19 cases in China was explained well by human mobility data. After the implementation of control measures, this correlation dropped and growth rates became negative in most locations, although shifts in the demographics of reported cases were still indicative of local chains of transmission outside of Wuhan. This study shows that the drastic control measures implemented in China substantially mitigated the spread of COVID-19. | |
| limitations | The majority of early cases (before 23 January 2020) reported outside of Wuhan had known travel history to Wuhan (57%) and were distributed across China, highlighting the importance of Wuhan as a major source of early cases. However, initial testing was focused mainly on travelers from Wuhan, potentially biasing estimates of travel related infections upward (see the materials and methods). | |
| generalisability | To test the contribution of the epidemic in Wuhan to seeding epidemics elsewhere in China, the authors built a naïve COVID-19 “generalized” linear model | |
| current knowledge (how the findings fit with current knowledge.) | - That travel restrictions are particularly useful in the early stage of an outbreak when it is confined to a certain area that acts as a major source. However, travel restrictions may be less effective once the outbreak is more widespread. - The combination of interventions implemented in China was clearly successful in mitigating spread and reducing local transmission of COVID-19, although in this work it was not possible to definitively determine the impact of each intervention. - Much further work is required to determine how to balance optimally the expected positive effect on public health with the negative impact on freedom of movement, the economy, and society at large. | |
| changes in diagnostic tests and access and any adjustment | The travel ban coincided with increased testing capacity across provinces in China. Therefore, an alternative hypothesis was that the observed epidemiological patterns outside of Wuhan were the result of increased testing capacity. The investigators tested this hypothesis by including differences in testing capacity before and after the rollout of large-scale testing in China on 20 January 2020. | |

1. **Nationwide Results of COVID-19 Contact Tracing in South Korea: Individual Participant Data From an Epidemiological Survey (Lee et al., 2020)**

| Data Collection Item | Item description / sub-groups | |
| --- | --- | --- |
| Bibiliographic Information | | |
| Author | Lee et al. | |
| Title | Nationwide Results of COVID-19 Contact Tracing in South Korea: Individual Participant Data From an Epidemiological Survey | |
| Year of publication | 2020 | |
| Country | South Korea | |
|  | | |
| Methods | | |
| Type of Study | Interrupted Time Series Analysis | |
| Time period and setting of the study | - Data were collected from individuals with laboratory-confirmed SARS-CoV-2 infection who subsequently completed the preliminary epidemiological surveillance conducted by each local government of South Korea (Seoul, Incheon, Sejong, Daegu, Gwangju, Ulsan, Busan, Gyeonggi-do, Gangwon-do, Chungcheongbuk-do [Chungbuk], Chungcheongnam-do [Chungnam], Gyeongsangbuk-do [Gyeongbuk], Gyeongsangnam-do [Gyeongnam], Jeollabuk-do [Jeonbuk], Jeollanam-do [Jeonnam], and Jeju) and the Korea Centers for Disease Control and Prevention (KCDC); Between January 19, 2020, and April 7, 2020. - The investigators set January 19, 2020, as the index date (epidemiologic day 1) and April 7, 2020, as epidemiologic day 80. - The authors divided the population into two distinct periods: before the social distancing campaign (January 19, 2020, to March 22, 2020) and after the social distancing campaign (March 23, 2020, to April 7, 2020). | |
| Intervention | Nationwide social distancing campaign | |
| description of intervention | March 22, 2020, indicates the launch of the social distancing campaign | |
| setting of the intervention | South Korea community | |
| Aim/ Objectives | To investigate the epidemiological characteristics of SARS-CoV-2 transmission in South Korea and evaluate whether a social distancing campaign is effective in mitigating the spread of COVID-19. | |
| Participant characteristics | Participants | - Individuals with laboratory-confirmed SARS-CoV-2 infection who subsequently completed the preliminary epidemiological surveillance conducted by each local government of South Korea. The final sample size was 2537 (1160 men and 1377 women) - A cluster infection was defined as a group of similar COVID-19 cases that occurred in the same area during a short time interval. (n= 1305) - Non-clustered cases were patients with COVID-19 unrelated to any other patients with COVID-19 in time or place (1232) |
|  | Comparator |  |
| Outcome measures | new confirmed COVID-19 | Laboratory confirmation of SARS-CoV-2 infection was defined as a positive result of real-time reverse transcriptase polymerase chain reaction assay of nasal or pharyngeal swabs, in agreement with the World Health Organization (WHO) guideline |
|  | mortality rate for COVID-19 |  |
| Source of data | - Epidemiological surveillance data were collected by epidemic intelligence service officers of each local government and the KCDC using the novel monitoring system that uses GPS (cell phone location), card transaction logs, CCTV, and a history medical facilities use. | |
| Data analyiss | - Interrupted time series analysis and the multivariable regression analysis were conducted to detect a change of slope after the launch of the nationwide social distancing campaign. - Network visualization was performed using Gephi version 0.9.2 - Statistical analyses were performed using SPSS version 25.0 (IBM Corp), and R software version 3.6.2 (R Foundation for Statistical Computing). A two-sided P value<.05 was considered statistically significant. | |
|  | | |
| Results | | |
| Study parameters | Percentage change of daily new confirmed COVID-19;  Percentage change of daily mortality rate for COVID-19. | |
| outcomes (Mean values for the main categories of estimated outcomes of interest, as well as mean differences between the comparator groups.) | - The mortality rate was significantly higher in cluster cases linked to medical facilities (11/143, 7.70% vs 5/1232, 0.41%; adjusted percentage difference 7.99%; 95% CI 5.83 to 10.14) and long-term care facilities (19/221, 8.60% vs 5/1232, 0.41%; adjusted percentage difference 7.56%; 95% CI 5.66 to 9.47) than in noncluster cases. - The change in trends of newly confirmed COVID-19 cases before and after the social distancing campaign was significantly negative in the entire cohort (adjusted trend difference –2.28; 95% CI –3.88 to –0.68) and the cluster infection group (adjusted trend difference –0.96; 95% CI –1.83 to –0.09). | |
| Characterising heterogeneity | Time to symptom onset or socioeconomic status. | |
|  | | |
| Discussion | | |
| Study findings, (Summarise key study findings and describe how they support the conclusions reached.) | - In a nationwide contact tracing study in South Korea, COVID-19 linked to medical and long-term care facilities significantly increased the risk of mortality compared to non-cluster COVID-19. - A social distancing campaign decreased the spread of COVID-19 in South Korea and differentially affected cluster infections of SARS-CoV-2. | |
| limitations | - First, our data did not contain clinical information because we could not link hospital data to the epidemiological survey expeditiously. - Second, we are still developing epidemiological surveys that include information on socioeconomic status (personal occupation and income) and time to development of COVID-19–related symptoms; hence, we were unable to analyse the time to symptom onset or socioeconomic status. - Third, although the WHO stated that contact tracing includes the process of identifying, assessing, and managing people who have been exposed to a disease to prevent onward transmission, we only had tracing from confirmed cases; tracing for exposure remains for future study. - Finally, epidemiological surveillance was not possible in some regions due to community-level outbreaks (Daegu and two cities in Gyeongbuk [Cheongdo and Gyeongsan]). Therefore, data from those regions were excluded. | |
| generalisability | Epidemiological surveillance was not possible in some regions due to community-level outbreaks (Daegu and two cities in Gyeongbuk [Cheongdo and Gyeongsan]). Therefore, data from those regions were excluded. This might influence its generalizibility. Moreover, our study shows that the social distancing campaign decreased the spread of COVID-19 in South Korea and differentially affected cluster infections of SARS-CoV-2. Therefore, our data may support driving public health policies in other countries and help normalize and restore social activities while minimizing the risk of transmission. | |
| current knowledge (how the findings fit with current knowledge.) | - COVID-19 linked to medical and long-term care facilities significantly increased the risk of mortality compared to non-cluster COVID-19. This study also showed that the social distancing campaign differentially affected cluster infections of SARS-CoV-2. Therefore, strategies for the prevention of cluster infection of SARS-CoV-2 should be personalized and comprehensive, and multidisciplinary strategies to prevent COVID-19 should be developed. In particular, special attention Need to be paid to prevent cluster infections of SARS-CoV-2, especially in medical and long-term care facilities. - An in-depth analysis of clustered cases revealed that a higher proportion of confirmed COVID-19 cases were related to religious, long-term care, and medical facilities. Cases from medical and long-term care facilities had a high mortality rate (11/143, 7.70% and 19/221, 8.60%, respectively) due to a higher proportion of vulnerable people including older adults and patients who are chronically ill present among these cases. These facilities are typically crowded with people in enclosed rooms, which create favorable conditions for transmission of respiratory diseases. | |
| changes in diagnostic tests and access and any adjustment | - | |

1. **Effects of policies and containment measures on control of COVID-19 epidemic in Chongqing (Liang et al., 2020)**

| Data Collection Item | Item description / sub-groups | |
| --- | --- | --- |
| Bibiliographic Information | | |
| Author | Liang et al. | |
| Title | Effects of policies and containment measures on control of COVID-19 epidemic in Chongqing | |
| Year of publication | 2020 | |
| Country | China | |
|  | | |
| Methods | | |
| Type of Study | Observational Study | |
| Time period and setting of the study | Epidemiological data on COVID-19 in Chongqing were prospectively collected from January 21 to March 15, 2020 | |
| Intervention | Different policies and containment measures | |
| description of intervention | - Quarantine focused on four types of high-risk populations: Confirmed cases, suspected cases, fever patients, and migrants. - The government organized the CDC, hospitals, and community clinics to enforce comprehensive and in-depth quarantine measures at transport entry points (such as traffic toll stations, airports, train stations, bus stations for long distance travel, and ports) by guarding the entrances and taking the temperature of all individuals entering and leaving, one by one. - Special inspections were carried out in public places, hospitals, shopping malls, and other important hubs. - Designated medical institutions and organized teams of medical experts were appointed, and a “one team of medical experts for each patient” treatment plan for critically ill cases was implemented to improve the treatment effect. - Locking down Wuhan on January 23 (14 days) - As of January 23 screening travellers with a fever at important transportation hubs, testing high-risk groups, and tracking close contacts in a timely manner. - At the end of January, the Chinese government announced an extension of the Spring Festival holiday and postponed the start of the new semester nationwide. - January 24, 2020: Initiated the first-level response to major public health emergencies. - The closed management of residential communities and travel restrictions for community residents were implemented beginning in early February (February 7). (14 days) | |
| setting of the intervention | - strict lockdown of Wuhan and the quarantine measures adopted by Chongqing | |
| Aim/ Objectives | To explore the effects of different policies and containment measures on the control of the COVID-19 epidemic in Chongqing. | |
| Participant characteristics | Participants | Chongqing is located in southwest China, bordering Hubei Province. It has 26 districts, 8 counties and 4 autonomous counties, with a total area of 82400 square kilometers. In 2019, the permanent population was 31243200, the urban population was 20869900 and the migrant population was 10280000. |
|  | Comparator details |  |
| Outcome measures | Incidence rate |  |
|  | Infection fatality rate |  |
|  | Suspected cases |  |
|  | Close contacts |  |
|  | People under medical observation |  |
|  | Inpatient cases |  |
|  | Discharged cases |  |
| Source of data | - Epidemiological data were collected from the official website of the Chongqing Municipal Health Commission from January 21, 2020 (the first date that a COVID-19 case was reported in Chongqing) to March 15, 2020 (when the epidemic was controlled). - Some of the management policy materials and their implementation dates for COVID-19 were collected from the official websites of the government and the Municipal Health Commission of Chongqing. Other policy materials on implementation and containment measures were obtained by querying the leaders and frontline workers at the CDC and local medical institutions. | |
| Data analyiss | - The descriptive analysis for this study was performed by Excel 2016 and included (1) The epidemic trend of the daily number of new confirmed cases, total confirmed cases, new discharged cases, total discharged cases, new close contacts, total close contacts, and cases under medical obeservation of COVID-19 in Chongqing; and (2) The trend (after logarithmic transformation) of the incidence rate, cumulative incidence rate, and composition ratio of severe cases, deaths, and discharged cases after diagnosis of COVID-19 in Chongqing. - The χ2 test was performed to test the differences in the incidence rate and infection fatality rate. - The data was analyzed using SAS 9.4 software (Copyright© 2020 SAS Institute Inc. Cary, NC, United States). - A significant difference was defined at an α level of 0.05. | |
|  | | |
| Results | | |
| Study parameters | The daily number of new confirmed cases, total confirmed cases, new discharged cases, total discharged cases, new close contacts, total close contacts, and cases under medical observation of COVID-19;  The trend (after logarithmic transformation) of the incidence rate, cumulative incidence rate, and composition ratio of severe cases, deaths, and discharged cases after diagnosis of COVID-19 in Chongqing. | |
| outcomes (Mean values for the main categories of estimated outcomes of interest, as well as mean differences between the comparator groups.) | - The lockdown of Wuhan significantly affected the COVID-19 epidemic trend in Chongqing. - From January 21 (5 new confirmed cases) to February 3 (37 new confirmed cases), the number of new confirmed cases of COVID-19 in Chongqing showed a daily rising trend. However, the number of new confirmed cases began to decline after February 5 (23 new confirmed cases), the 14th day after the lockdown of Wuhan and the end of the incubation period for cases imported from Hubei Province to Chongqing. - Second, the implementation of the closed management of residential communities in Chongqing further effectively controlled the occurrence of new confirmed cases. After February 5 (23 new confirmed cases), new confirmed cases were mainly second generation local cases. On February 7 (15 new confirmed cases), the Chongqing municipal government announced that it would impose closed management of residential communities throughout the city. After an average incubation period (7 d), the number of new confirmed cases dropped to 8 on February 14, and on February 18, the number dropped to 2. - From January 29 (the first recovered case) to February 23 (335 discharged cases), the discharge rate of COVID-19 patients in Chongqing increased significantly to 58.26% (from 0.61%, P < 0.001). - From January 31 to February 22, there were 6 deaths (infection fatality rate: 0.42% to 1.05%, P = 0.380), and the number of deaths has not continued to increase. The infection fatality rate of COVID-19 in Chongqing was lower than that in Wuhan and several other provinces and cities, an outcome significantly related to the prevention and control measures implemented by designated medical institutions and the integration and distribution of medical resources by the government. - From January 21 (72 new close contacts, 0 new suspected cases) to February 1 (1459 new close contacts, 175 new suspected cases), with the confirmation of the cases imported from Hubei Province and the development of epidemiological investigations, the number of new close contacts and suspected cases increased gradually and showed a certain downward trend by February 5 (924 new close contacts, 171 new suspected cases). Eight days (February 15) after the implementation of the closed management of residential communities (starting on February 7), the number of new close contacts (431) decreased significantly and has remained at a low level since then [1743 (5.58/100000) vs 431 (1.38/100000), P < 0.001]. - From the beginning of the lockdown of Wuhan to February 5, the number of people under medical observation increased significantly each day, and the number was 7239 on February 5. Seven days after the implementation of the closed management of residential communities [starting on February 7 (7474 people under medical observation)], the number of people under medical observation began to decline and reached 1120 on February 23 [7474 (23.92/100000) vs 1120 (3.59/100000), P < 0.001]. - There were 2 critically ill patients for every 6 confirmed cases on January 22 in Chongqing, revealing a high ratio of critically ill cases. After the adoption of the “one team of medical experts for each patient” treatment plan for critically ill cases and the optimization of the allocation of medical resources and the diagnosis and treatment plan, the ratio of critically ill patients was well controlled and slowly decreased, the discharge rate increased rapidly each day, and the death rate was well controlled. - As of February 23, the cumulative incidence of COVID-19 in Chongqing was 1.84/100000 (575 cases), and the infection fatality rate was 1.04% (6/575). The incidence rate continued to increase from January 21 to January 31 (5 (0.02/100000) vs 32 (0.10/100000), P < 0.001) and gradually declined after February 7 (32 (0.10/100000) vs 15 (0.07/100000), P = 0.013). The upward trend of cumulative incidence flattened out. After the closed management of residential communities was implemented (starting on February 15 (0.054/100000)), the incidence rate showed a more significant downward trend and fell to 0.006/100000 (P = 0.002) by February 18. | |
| Characterising heterogeneity | - | |
|  | | |
| Discussion | | |
| Study findings, (Summarise key study findings and describe how they support the conclusions reached.) | - As of March 15, the cumulative incidence of COVID-19 in Chongqing was 1.84/100000 (576 cases), and the infection fatality rate was 1.04% (6/576). - The spread of COVID-19 was controlled by effective policies that involved establishing a group for directing the COVID-19 epidemic control effort; strengthening guidance and supervision; ensuring the supply of daily necessities and medical supplies and equipment to residents; setting up designated hospitals; implementing legal measures; and enhancing health education. - The prevention policies and containment measures implemented by the government and medical institutions are highly effective in controlling the spread of the epidemic and increasing the recovery rate of COVID-19 patients. | |
| limitations | - First, this study did not explore emergency networks combined with different institutes to respond to the control of emergent infectious diseases. - Second, it did not examine medical insurance in the context of emergent infectious diseases. | |
| generalisability |  | |
| current knowledge (how the findings fit with current knowledge.) | - Similar to the prevention and treatment strategies used for SARS, the measures implemented in Chongqing (such as naming designated medical institutions and organizing medical expert teams, implementing a “one team of experts for each patient” treatment plan for critically ill cases, receiving and treating confirmed patients quickly, and optimizing the diagnosis and treatment plan continuously) significantly improved the cure rate and reduced the infection fatality rate of COVID-19. - Health resources have inevitable impacts on the control and treatment of COVID-19. The Chinese government provided adequate health resources for fighting the COVID-19 pandemic and covered all medical costs associated with NAT, centralized isolation, and the care of suspected and confirmed cases. It also provided additional salary subsidies for medical staff participating in the prevention, control, and treatment of COVID-19, which ensured that every high-risk person and confirmed case could be effectively managed and treated and reduced the spread of infection. - In addition, the measures taken by the Chongqing government not only included the correct guidance of media publicity to instill a common understanding in the populace but also involved strengthening awareness of the law regarding the prevention and transmission containment to curb malicious manmade disease spread. | |
| changes in diagnostic tests and access and any adjustment | - Optimizing the diagnosis process, shortening the diagnosis time, and constructing teams of clinical experts facilitated the provision of “one team of medical experts for each patient” treatment for severe cases, which significantly improved the recovery rate and reduced the infection fatality rate. - The CDC improved the test plan, optimized the test process, and achieved the goal of “completing testing of accumulated samples and immediately testing the new samples”, shortening the diagnosis time of suspected cases. To expand NAT capacity, the CDC trained staff from hospitals and community health centers on how to collect pharyngeal samples, nasopharyngeal samples, and anal samples. | |

1. **Enacting national social distancing policies corresponds with dramatic reduction in COVID19 infection rates (McGrail et al., 2020)**

| Data Collection Item | Item description / sub-groups | |
| --- | --- | --- |
| Bibiliographic Information | | |
| Author | McGrail et al. | |
| Title | Enacting national social distancing policies corresponds with dramatic reduction in COVID19 infection rates | |
| Year of publication | 2020 | |
| Country | USA | |
|  | | |
| Methods | | |
| Type of Study | Observational | |
| Time period and setting of the study | US; June 5th 2020   - The initial time point was considered as when countries exceeded 1 case per million inhabitants. - Post social distancing was considered 11 days after social distancing policy was enacted based on disease latency days | |
| Intervention | social distancing | |
| description of intervention | Idaho enacted social distancing policies on March 25th 2020, whereas no such policies were enacted in Nebraska. | |
| setting of the intervention | United States' Community; Idaho | |
| Aim/ Objectives | To demonstrate that implementation of social distancing policies in US states corresponded with a reduction in COVID19 spread rates, and that the reduction in spread rate is proportional to the average change in mobility. | |
| Participant characteristics | Intervention group | Idaho with 1.787 million population |
|  | Comparator details | Nebraska with 1.934 million population |
| Outcome measures | COVID19 spread rate | COVID19 spread rate defined by an exponential growth function |
| Source of data | - Daily case numbers for COVID19 and population numbers were acquired from the COVID-19 Data Repository by the Center for Systems Science and Engineering (CSSE) at Johns Hopkins University (https://github.com/CSSEGISandData/COVID-19). - Social distancing policies were acquired from <https://auravision.ai/covid19-lockdowntracker/>. - Mobility data were acquired from Google mobility reports (https://www.google.com/covid19/mobility/). Average mobility was taken as the average reduction in mobility across the 5 Google mobility metrics (retail and recreation, grocery and pharmacy, parks, transit stations, and workplace). - For regions with multiple sub-region values, all values were averaged. Testing data was acquired from the Our World In Data source data repository (https://github.com/owid/covid-19-data/tree/master/public/data/). | |
| Data analyiss | - Correlations were assessed with Spearman correlation coefficient. Paired analysis was performed using a signed-rank test. - All data analysis was performed in MATLAB R2019a. US heat map of COVID19 spread rate was generated in R v3.6 using the packages “maps” and “ggplot2”. | |
|  | | |
| Results | | |
| Study parameters | exponential growth function, average mobility, Change in average mobility, COVID19 spread rate (Median with interquartile range) | |
| outcomes (Mean values for the main categories of estimated outcomes of interest, as well as mean differences between the comparator groups.) | - In Idaho, pre-social distancing COVID19 spread rate was 0.29, dropping to 0.03 following social distancing for a net change of -0.26. When analyzing spread rate in Nebraska over equivalent time periods we found that Nebraska only dropped from 0.17 to 0.10, for a net change of -0.07, 3-fold lower than observed in Idaho. - Notably, the two smallest reductions in COVID19 spread rates were observed in states without social distancing policies (South Dakota and Nebraska), with Wyoming having the 15th smallest reduction. - In the 47 states with social distancing policies we observed a strong reduction in average mobility following implementation of social distancing policies (P = 2.4x10-9). - While states without social distancing policies also showed decreased mobility over the same time frame, the observed decrease was significantly less, suggesting social distancing policies effectively reduce community mobility. Critically, changes in average mobility were significantly correlated with decreases in COVID19 spread rate. | |
| Characterising heterogeneity | Population demographics and densities, travel rates, variations in testing accessibility, weather patterns, and …. | |
|  | | |
| Discussion | | |
| Study findings, (Summarise key study findings and describe how they support the conclusions reached.) | Although social distancing policies may have negative economic impacts, this analysis suggests that this containment approach has yielded significant positive health outcomes. | |
| limitations | A critical limitation of this study is reliance on direct COVID19 testing, which likely underestimate prevalence when compared to antibody-based serology testing approaches. Numerous additional factors such as population density, healthcare infrastructure, testing rates, climate, population characteristics, and more, likely contribute to rate of COVID19 spread, this study focused on the change in COVID19 spread rate following implementation of social distancing policies as an internal control for these variables. | |
| generalisability |  | |
| current knowledge (how the findings fit with current knowledge.) | Analysis of COVID19 spread rates may be complicated by numerous features, including population demographics and densities, travel rates, variations in testing accessibility, weather patterns, and likely many more parameters yet to be identified. To account for these factors, we determined the spread of COVID19 both before and after implementation of social distancing policies. | |
| changes in diagnostic tests and access and any adjustment | - | |

1. **Impact of COVID-19 epidemic curtailment strategies in selected Indian states: An analysis by reproduction number and doubling time with incidence modelling (Mitra et al., 2020)**

| Data Collection Item | Item description / sub-groups | |
| --- | --- | --- |
| Bibiliographic Information | | |
| Author | Mitra et al. | |
| Title | Impact of COVID-19 epidemic curtailment strategies in selected Indian states: An analysis by reproduction number and doubling time with incidence modelling | |
| Year of publication | 2020 | |
| Country | India | |
|  | | |
| Methods | | |
| Type of Study | Observational | |
| Time period and setting of the study | - Ten states of India; 1rd March as of 23rd April 2020. - We divided the timeline of the epidemic into three phases. The first phase was before lockdown i.e. 25th March 2020, the second phase was the early epidemic phase (15 days into the lockdown), and the third phase was till day-30 of the lockdown. - The last access to the database was made on 1st May 2020. The data were truncated up to 23rd April 2020 for this study. This buffer period of 7 days offered some immunity against the possible delay to add the cases and the limitation to access the data in real-time. | |
| Intervention | Lockdown | |
| description of intervention | The nationwide lockdown was enforced in India on 24th March 2020 resulting in restrictions on unnecessary travel, closure of schools, colleges, and the prohibition of mass gatherings. | |
| setting of the intervention |  | |
| Aim/ Objectives | To estimate the time-varying reproduction number (R(t)) and doubling time before the commencement of lockdown, 15 days into the lockdown (early epidemic) and at day-30 of the lockdown to see the cumulative effect of curtailment strategies (inclusive of lockdown) in selected states. | |
| Participant characteristics | Participants | - Ten states population size 49.39 million (Andhra Pradesh), 18.98 million (Delhi), 62.7 million (Gujarat), 73.34 million (Madhya Pradesh), 114.2 million (Maharashtra), 68.89 million (Rajasthan), 67.86 million (Tamil Nadu), 35.19 million (Telangana), 204.2 million (Uttar Pradesh), 90.32 million (West Bengal). - 20,590 cases (89.4%) were seen in the selected 10 states. The proportion of imported cases was less than 2% in all the 10 states |
|  | Comparator details |  |
| Outcome measures | Time-varying  reproduction numbers (R(t) | The eligibility for testing includes patients presenting with suspected symptoms  in hospitals, exposed healthcare workers as well as contacts identified through contact  tracing. |
|  | Growth Rate |  |
|  | Doubling Time |  |
| Source of data | The data source used for this study is compiled from these state bulletins, official handles of state governments, and health ministries and maintained at www.covid19india.org | |
| Data analyiss | - After preparing the data for analysis, R0 was estimated using maximum likelihood (ML) method which is based on the expectation minimum algorithm where the distribution probability of secondary cases is maximized using the serial interval discretization. - The doubling time and growth rate were estimated by the natural log transformation of the exponential growth equation. | |
|  | | |
| Results | | |
| Study parameters | The daily new cases (daily incidence);  Effective reproduction number (*R(t)*) at 15 days and 30 days into lockdown. The respective doubling time is also shown at these time points. | |
| outcomes (Mean values for the main categories of estimated outcomes of interest, as well as mean differences between the comparator groups.) | - Five states reported an increase in doubling time, and four states reported negligible change in doubling time. The state of Gujarat reported a decrease in doubling time which could mean that there is no slowdown of the outbreak. - Seven of the ten selected states saw a reduction in reproduction number (R(t)) between the early epidemic phase and the current timeframe. - The highest decrease in R(t) was seen in Andhra Pradesh (73%) followed by Delhi (43%) and Rajasthan (30%). Telangana and Tamil Nadu saw stable R(t) during this time period while Gujarat, on the other hand, saw an increase. - The growth rates of 8 of 10 states showed a decline between the two time intervals. Uttar Pradesh did not show a decline in growth rate, whereas Gujarat showed an increase. | |
| Characterising heterogeneity | - | |
|  | | |
| Discussion | | |
| Study findings, (Summarise key study findings and describe how they support the conclusions reached.) | - The time-varying reproduction number (R(t)) in several states has come down by the adopted curtailment strategies, including lockdown compared to what was estimated at the beginning of the epidemic. As the final epidemic size’s relation with R(t) is exponential and not linear, this reduction if sustained, may considerably decrease the total number of affected persons compared to initial estimates. - Two factors should be considered at this moment. Firstly, the R(t) needs to be further reduced in-order to flatten or change the trajectory of the epidemic curve, and the one may perceive the state-wise variations in its magnitude. Secondly, although the doubling time has increased in relative terms, the epidemic still follows an exponential trajectory, and the current daily incidence is much more as compared to the beginning of the epidemic. | |
| limitations | - The credibility of a crowd-sourced dataset may be viewed from the following perspectives: under-reporting, duplicated / redundant information, incomplete information, differential lag in reporting the cases, missing initial cases, the inclusion of imported cases as native cases, and partisan information. These may lead to overestimation or underestimation of reproduction numbers. - The estimates might be influenced by certain effect modifiers and confounders like population density, climatic variations and violation of the assumption of random mixing. | |
| generalisability |  | |
| current knowledge (how the findings fit with current knowledge.) | - The time varying reproduction number (R(t)) estimations are dynamic and may change over age structure, time and nature of the intervention. R(t) is a measure of transmissibility or contagiousness at a given period, and its reduction should be interpreted with caution. This is indicative of the relative force of infection at a given time while the ‘absolute’ burden of infections also depend on the duration of infectiousness and progression of time from the first reported case by influencing - The mixing probability of infected-infectee pair is further influenced by population density, mobility patterns and the general population’s compliance with the non-pharmaceutical interventions (NPIs). - When non-pharmaceutical interventions (NPIs) are enforced, there is a reduction in the number of potential contacts and thereby reducing the R(t). However, in a scenario where R(t) > 1, and the number of actively infected persons is high, cases will still rise as one person transmits the infection to one more person. Therefore, in the post-lockdown era, it might be a challenge to maintain this path, and this may be the period where the absolute burden of the infected persons will be high. - Considering the undesired collateral effects of stringent restrictions on the economy and livelihoods of the general population; a nationwide lockdown may not be a feasible solution for a longer duration. - The decision on which NPI measure should be enforced should vary with the burden of active infections, emerging patterns of severity /mortality, and health system endurance and capacity to deal with such cases embedded in socio-economic and socio-cultural vulnerability. | |
| changes in diagnostic tests and access and any adjustment | - | |

1. **Association of State Stay-at-Home Orders and State-Level African American Population With COVID-19 Case Rates(Padalabalanarayanan et al., 2020)**

| Data Collection Item | Item description / sub-groups | |
| --- | --- | --- |
| Bibiliographic Information | | |
| Author | Padalabalanarayanan et al. | |
| Title | Association of State Stay-at-Home Orders and State-Level African American Population With COVID-19 Case Rates | |
| Year of publication | 2020 | |
| Country | USA | |
|  | | |
| Methods | | |
| Type of Study | Cross-sectional study | |
| Time period and setting of the study | Data from March 1 to May 4, 2020, for all states (except Washington state) as well as the District of Columbia were used. | |
| Intervention | Stay-at-home order (SAHO) | |
| description of intervention | During March and April, most states in the US imposed shutdowns and enacted stay-at-home orders (SAHOs) in an effort to control the disease. However, mixed messages from political authorities on the policy usefulness, popular pressure, as well as concerns about the economic fallout2 led some states to lift SAHOs before public health experts considered it advisable. The subsequent increase in infections and fatalities in several states has led some experts to speculate that another round of shutdowns and SAHOs may be necessary to control the disease spread. | |
| setting of the intervention |  | |
| Aim/ Objectives | To investigate whether state-imposed stay-at-home orders (SAHOs) and the proportion of African American population in a state were associated with the state-level COVID-19 cases. | |
| Participant characteristics | Participants | The final sample included 3023 pooled state- and day-level observations. The mean (SD) cumulative positive case rate was 103.186 (200.067) cases per 100 000 state population, the mean (SD) cumulative test rate was 744.23 (894.944) tests per 100 000 state population, and the mean (SD) subsequent cumulative fatality rate was 12.923 (21.737) deaths per 100 000 state population. |
|  | Comparator details |  |
| Outcome measures | daily cumulative COVID-19 case  rates | Cumulative case rate per 100 000 state population, measured daily |
|  | COVID-19 fatality rates | Derived using mean cumulative fatality rates 21 to 28 days after each date |
| Source of data | - Data on cumulative COVID-19 positive cases (hereafter, cases), cumulative COVID-19 tests, and cumulative fatalities for each day were obtained from the COVID Tracking Project. - Initiated by The Atlantic in partnership with Related Sciences, the COVID Tracking Project collates data from state health agencies and makes it publicly available. Cumulative indicates the sum of all daily cases and tests reported for that state up to that day. | |
| Data analyiss | - Multivariate regression models were estimated with random intercepts to account for repeated daily observations from each state. - Two-sided P = .05 was set as the threshold for statistical significance for hypothesis testing. All models were estimated using Stata statistical software version 16 (StataCorp). | |
|  | | |
| Results | | |
| Study parameters | Cumulative case rate mean (SD) per 100 000 population;  Cumulative test rate mean (SD) per 100 000 population;  Cumulative fatality rate mean (SD) per 100 000 population;  The proportion of African American populations in state populations mean (SD). | |
| outcomes (Mean values for the main categories of estimated outcomes of interest, as well as mean differences between the comparator groups.) | - -There was a negative association of SAHOs with cumulative case rates (β = −1.166; 95%CI, −1.484 to −0.847; P < .001) and subsequent fatality rates (β = −0.204; 95%CI, −0.294 to −0.113; P < .001). - Estimation analyses indicated that expected cumulative case rates would have been more than 200% higher and fatality rates approximately 22%higher if there were no SAHOs, as compared with SAHOs fully in place. - A higher proportion of African American population was associated with higher case rates (β = 0.045; 95%CI, 0.014 to 0.077; P = .001) and fatality rates (β = 0.068; 95%CI, 0.044 to 0.091; P < .001). | |
| Characterising heterogeneity | States with higher proportions of African American populations were associated with higher case rates (model 1: β = 0.045; 95%CI, 0.014 to 0.077; P = .005; model 2: β = 0.048; 95%CI, 0.019 to 0.077; P = .001) and fatality rates (model 3: β = 0.068; 95%CI, 0.044 to 0.091; P < .001; model 4: β = 0.064; 95%CI, 0.041 to 0.088; P < .001). Converted to percentage changes, this implied that a 1–percentage point increase in a state’s African American population was associated with a mean of 4.6%(95%CI, 1.4%-8.0%) higher cumulative cases and 7.0% (95%CI, 4.5%-9.5%) higher fatalities. | |
|  | | |
| Discussion | | |
| Study findings, (Summarise key study findings and describe how they support the conclusions reached.) | Results from multivariate regression models indicated that stay-at-home orders were associated with reductions in cumulative COVID-19 case rates.  States with larger African American populations had higher COVID-19 case rates. | |
| limitations | - First, how strongly SAHOs were enforced and adhered to and what types of businesses were deemed essential and allowed to stay open may have varied across states. - Second, we could not control for local stay-at-home ordinances at the city or county level, owing to a lack of reliable information on such ordinances or what proportion of the state’s population was impacted by them. - Third, testing capacity, protocols, and the speed and accuracy of reporting tests, positive cases, and fatalities may not have been uniform across states or over time. - Fourth, we operationalize subsequent fatality rates as those occurring 21 to 28 days after cases, and results might be sensitive to alternate specifications. - Fifth, our supplemental findings using inferred infection rates should be treated with caution, as the science on how to estimate true underlying infections from existing information is still evolving; additionally, infection-fatality ratios can vary by age and race, but breakdowns by age and race for cases, tests, or fatalities were not available during this study period. - Sixth, since our focus was on SAHOs, we did not consider patterns in COVID-19 cases and fatalities from more recent months. | |
| generalisability |  | |
| current knowledge (how the findings fit with current knowledge.) | - While the high economic cost makes SAHOs unsustainable as long-term policy, these findings could help inform federal, state, and local policy makers in weighing the costs and benefits of different short-term options to combat the pandemic. These findings also emphasize the importance of understanding and addressing the drivers of racial disparities in COVID-19 outcomes as part of the overarching goal of improving health equity in the US. - Some of the control variables yielded unintuitive results. For example, state poverty rates had a counterintuitive negative association with case rates in one model, although it was statistically insignificant in the others; and diabetes prevalence had no statistical significance. Some studies reported similar counterintuitive results | |
| changes in diagnostic tests and access and any adjustment | - All regression models included cumulative test rates per 100 000 population. Given the variations in testing capacity and protocols for whom to test, both across states and within states over the study period,20 changes in detected cases following SAHOs may be an artifact of changes in testing; therefore, controlling for level of testing is critical. - Testing capacity, protocols, and the speed and accuracy of reporting tests, positive cases, and fatalities may not have been uniform across states or over time. | |

1. **Association of Public Health Interventions with the Epidemiology of the COVID-19 Outbreak in Wuhan, China (Pan et al., 2020)**

| Data Collection Item | Item description / sub-groups | |
| --- | --- | --- |
| Bibiliographic Information | | |
| Author | Pan et al. | |
| Title | Association of Public Health Interventions With the Epidemiology of the COVID-19 Outbreak in Wuhan, China | |
| Year of publication | 2020 | |
| Country | China | |
|  | | |
| Methods | | |
| Type of Study | Cohort study | |
| Time period and setting of the study | between December 8, 2019, and March 8, 2020, Wuhan, China | |
| Intervention | Non-pharmaceutical public health interventions | |
| description of intervention | - Cordons sanitaire, traffic restriction, social distancing, home confinement, centralized quarantine, and universal symptom survey. - January 10 to 22 (massive human movement due to the Chinese New Year holiday) - January 23 to February 1 (cordons sanitaire, traffic restriction and home quarantine) - February 2 to 16 (centralized quarantine and treatment) | |
| setting of the intervention | Community of Wuhan | |
| Aim/ Objectives | To evaluate the association of public health interventions with the epidemiological features of the COVID-19 outbreak in Wuhan by 5 periods according to key events and interventions. | |
| Participant characteristics | Intervention group | 32 583 patients with laboratory-confirmed COVID-19 in Wuhan from December 8, 2019, through March 8, 2020, (258 cases had missing values and were not included in the clinical severity analyses). |
|  | Comparator details |  |
| Outcome measures | Rates of laboratory-confirmed COVID-19 infections | - Defined as the number of cases per day per million people, across age, sex, and geographic locations. - A confirmed case was defined based on epidemiological history (including cluster transmission), clinical manifestations (fever and respiratory symptoms; laboratory evidence of normal or decreased number of leukocytes and/or lymphopenia), lung imaging, and results of SARS-CoV-2 nucleic acid detection. In the 7th edition, detection of serum-specific antibodies was further added |
|  | Effective reproduction number | The effective reproduction number Rt was defined as the mean number of secondary cases generated by a typical primary case at time t in a population. |
| Source of data | Laboratory-confirmed COVID-19 cases were extracted from the municipal Notifiable Disease Report System, including patients’ age, sex, residential location, occupation, and severity classification. | |
| Data analyiss | - The Rt was calculated using the method developed by Cori et al17 in R version 3.6.2 (R Foundation for Statistical Computing). - The daily number of reported COVID-19 cases and the serial interval (mean, 7.5days [SD, 3.4days]; constant across periods), derived from a previous epidemiological survey of the first 425 cases in Wuhan, were used to estimate Rt and its 95% credible interval on each day via a 5-day moving average. - The Rt was calculated for the whole period, but results were Shown beginning with January 1,2020, given the limited number of diagnosed cases and limited diagnosis capacity in December 2019. | |
|  | | |
| Results | | |
| Study parameters | number of cases per day per million people; | |
| outcomes (Mean values for the main categories of estimated outcomes of interest, as well as mean differences between the comparator groups.) | - The daily confirmed case rate over the whole period in local health care workers (130.5 per million people [95% CI, 123.9-137.2]) was higher than that in the general population (41.5 per million people [95% CI, 41.0-41.9]). - The proportion of severe and critical cases decreased from 53.1% to 10.3% over the 5 periods. - The severity risk increased with age: compared with those aged 20 to 39 years (proportion of severe and critical cases, 12.1%), elderly people (≥80 years) had a higher risk of having severe or critical disease (proportion, 41.3%; risk ratio, 3.61 [95% CI, 3.31-3.95]) while younger people (<20 years) had a lower risk (proportion, 4.1%; risk ratio, 0.47 [95% CI, 0.31-0.70]). - The effective reproduction number fluctuated above 3.0 before January 26, decreased to below 1.0 after February 6, and decreased further to less than 0.3 after March 1. | |
| Characterising heterogeneity | - There were strong geographic differences in rates of confirmed cases, with the highest rates in the urban districts. - The daily rate of cases in local health care workers (130.5 per million people [95%CI, 123.9-137.2]) was higher than that in the general population (41.5 per million people [95% CI, 41.0-41.9]) over the whole period. - Rates of confirmed cases and trends also differed by age. - Similar patterns were observed for men and women, with a slightly higher rate in women (43.7 confirmed case rate per million [95% CI, 43.0-44.4]) compared with men (39.4 confirmed case rate per million [95% CI, 38.8-40.0]) over the whole period. | |
|  | | |
| Discussion | | |
| Study findings, (Summarise key study findings and describe how they support the conclusions reached.) | A series of multifaceted public health interventions was temporally associated with improved control of the COVID-19 outbreak in Wuhan and may inform public health policy in other countries and regions. | |
| limitations | - First, the Chinese government implemented multiple interventions at the same time or in a short timeframe to control the outbreak, and thus individual strategies could not be evaluated. - In addition, the observational study design precludes causal inference. However, clinical trials were not feasible or ethical under such public health emergencies, and there are not yet data available to compare the experience in Wuhan with other outbreak areas pursuing different policies. - Second, data were extracted from the infectious disease reporting system, and no information was available for other epidemiological variables and clinical characteristics, such as incubation period, time to hospitalization, time to discharge, medical treatment strategies, and vital status. Cases occurring in the early days of a new period included infections that were acquired during the previous period, and thus there were lags for the interventions to take effect. - Third, there were unexplained peaks in the epidemic curve, particularly a surge on February 1. - Fourth, no data were available on the diagnostic testing pattern, ascertainment rate, and proportion of asymptomatic cases. | |
| generalisability |  | |
| current knowledge (how the findings fit with current knowledge.) | - The rate of cases in health care workers was substantially higher than in the general population between January 11 and February 1, indicating a high risk of nosocomial infection. The rate of confirmed cases among local health care workers quickly decreased in the later periods, after increasing awareness of and wider use of personal protective equipment, proper training, adequate hospital-level prevention and management, and support from more than 30000 health care workers from other provinces of China. Furthermore, none of the health care workers from other provinces were infected, supporting the importance of carefully protecting health care workers in the outbreak of a high transmissible infectious disease. - Despite these interventions, the confirmed case rate continued to increase in the third period, perhaps in part due to shortages of pharmaceuticals and medical equipment and delayed diagnosis and access to medical treatment. Without rapid diagnosis, the risk of cross-infection in hospitals was high and patients likely continued to infect family members and close contacts. - Centralized quarantine of patients and close contacts appears to have been associated with a reduction in in-hospital, household, and community transmission. A door-to-door and individual-to-individual universal symptom survey to single out presumptive cases in the community, between February 16 and 18, was associated with further reductions in the spread of COVID-19 in Wuhan. | |
| changes in diagnostic tests and access and any adjustment | The shortage of testing in the early periods suggests that ascertainment bias may in part explain the initially high proportion of severe and critical cases; testing was widely available in the fourth and fifth periods and these estimates may reflect a more accurate measure of disease severity. | |

1. **Impact of nonpharmacological interventions on COVID-19 transmission dynamics in India (Patel et al., 2020)**

| Data Collection Item | Item description / sub-groups | |
| --- | --- | --- |
| Bibiliographic Information | | |
| Author | Patel et al. | |
| Title | Impact of nonpharmacological interventions on COVID-19 transmission dynamics in India | |
| Year of publication | 2020 | |
| Country | India | |
|  | | |
| Methods | | |
| Type of Study | Observational | |
| Time period and setting of the study | India- January 30 to May 4, 2020 (day 96 since epidemic start) | |
| Intervention | Preventive public health measures | |
| description of intervention | Various public health measures were classified into surveillance strengthening, testing strategy, containment activities, travel and mobility restrictions, social distancing, and health promotion.   - India’s COVID‑19 response began with preventive public health measures as early as January 17, 2020. - Major surveillance strengthening measures were– thermal screening and distribution of symptom self‑declaration forms for passengers coming from China at selected airports (January 17) which expanded to other airports and travellers from other countries as the epidemic grew internationally. - In addition, mandatory quarantine of passengers for 14 days (March 13), and rolling out of digital contact tracing application (April 2) was initiated. - Guidelines and trainings on COVID‑19 surveillance and treatment were provided throughout. The initial testing strategy included all symptomatic patients with travel history and their contacts (March 9) which were expanded to symptomatic health‑care workers caring for COVID‑19 cases (March 17) and hospitalized severe acute respiratory infection (SARI) cases and asymptomatic high‑risk contacts of cases (March 21). Pooled sample testing (April 13) in containment zones was also added. A cluster‑containment strategy was rolled out to contain local transmission (March 02). - Travel restrictions began with an advisory to avoid nonessential travel to China (February 5), expanded to other affected countries, and culminated in avoidance of all international travels (March 10). These followed by international land border check-post closure (March 15) and complete stoppage of incoming international flights (March 22). - Social distancing measures began with advisory against mass gathering (March 5) followed by school, sports, and entertainment centers closure and social distancing advisory at work place and commercial establishments (March 16). Complete internal mobility restriction for 21 days (lockdown) began on March 25 (Phase 1) and extended for 19 days from April 15, 2020 (Phase 2). From May 4, it was further extended (Phase 3) till May 17, 2020. Unlike Phase 1, conditional relaxation in mobility within areas not designated as COVID‑19 hotspots by the respective state or district administrations was allowed during Phase 2, from April 20, 2020. In Phase 3, based on number of cases reported during the past 21 days, the country was divided into three color‑coded zones and restriction was lifted accordingly. - Implementation of all these measures was further enforced by the invocation of Epidemic Diseases Act 1897 and Disaster Management Act, 2005 (March 11). Intense health promotion efforts were done using print and digital mass media. | |
| setting of the intervention | Community of India | |
| Aim/ Objectives | To study the impact of NPI on transmission dynamics of COVID‑19 epidemic in India and estimated the minimum level of herd immunity required to halt it | |
| Participant characteristics | Participants | Recorded number of daily confirmed cases till May 4, 2020 (day 96 since epidemic start) and prepared an epidemic curve. |
|  | Comparator details |  |
| Outcome measures | Growth rate | Defined as laboratory confirmation of COVID‑19 infection in a person with reverse transcription‑polymerase chain reaction test. |
|  | time‑dependent effective reproduction number (Rt) | Rt takes into account both susceptible and nonsusceptible populations. |
|  | Doubling time |  |
| Source of data | Nation‑wide data were being collected by special active surveillance setup by the Integrated Disease Surveillance Programme | |
| Data analyiss | - An inbuilt library (R0) in statistical software R (version Rx 64.3.6.3) was used to provide a standardized approach. We used maximum likelihood (ML) estimation to calculate the R0 value. - An inbuilt function was used given in software R (version Rx 64.3.6.3), “est.R0.TD” to generate Rt. | |
|  | | |
| Results | | |
| Study parameters |  | |
| outcomes (Mean values for the main categories of estimated outcomes of interest, as well as mean differences between the comparator groups.) | - The growth rate was 21% on March 24, 12% on April 14, and steadily reduced to 6% as of May 4, 2020. - The R0 was estimated to be 2.38 (95% CI = 1.79–3.07) based on the early transmission dynamics of the first 100 cases. - The estimated Rt was 1.53 (95% CI = 1–2) at the beginning of the outbreak. A single, sudden peak was noted at Rt = 10.36 (95% CI = 8–12.5) on March 3 (day 34) which reduced to 1.67 (95% CI = 2.06–1.27) in the next 2 days. - India reported the first 100 cases on March 15, corresponding with the estimated Rt = 2.51 (95% CI = 2.06–3.14). On - April 2, 8 days after Phase 1 lockdown, the estimated Rt decreased to 1.91 (95% CI = 1.80–2.02). At the end of Phase 1 (April 14) and Phase 2 (May 3) of lockdown, Rt was 1.28 (95% CI = 1.22–1.32) and 1.83 (95% CI = 1.71–1.93). - As of May 4, 2020, latest Rt = 2.04 (95% CI = 1.83–2.21) was estimated. - Doubling time varied (2–9.5 days) in the initial days due to imported cases. Average doubling time increased from 4.3 days (SD = 1.86) before lockdown to 5.4 days (SD = 1.03) and 10.9 days (SD = 2.19) during Phase 1 and Phase 2 of lockdown, respectively. It was 13.6 on May 4, 2020. Based on the Rt value at the end of Phase 2, we estimated Pcrit = 0.45. - It amounts to about 621 million COVID‑19 recoveries in the Indian population to achieve herd immunity. | |
| Characterising heterogeneity | - | |
|  | | |
| Discussion | | |
| Study findings, (Summarise key study findings and describe how they support the conclusions reached.) | - India’s health system responded to COVID‑19 pandemic with swiftness. Thus far, the fallouts of the pandemic have been less severe than countries with more robust health systems. - The nationwide lockdown has shown the desired effect of slowing the epidemic. As we transit through phase‑wise exit strategy, we should continue to consolidate on our gains by way of increased testing, persistent contact tracing and isolating, modulating movement restrictions while protecting the vulnerable population, and continuous monitoring of transmission dynamics. | |
| limitations | - We report limitations in Rt estimates due to the unavailability of data on symptom onset for all the cases to achieve a serial interval distribution overtime. Its interpretation should be considered against the evolving testing strategy. - We have also assumed that measures and advisories issued by the government were executed timely, uniformly, and successfully throughout the country, which could not be verified independently. | |
| generalisability |  | |
| current knowledge (how the findings fit with current knowledge.) | - An observed decline in Rt following NPI implementation, confirming subexponential growth akin to a mitigated epidemic; however, Rt >1 indicated ongoing disease transmission. - During Phase 1, a continuous decline in Rt was seen till the end (April 14). Rt in Phase 2 varied (1.27–1.83) but remained below 2. Minor fluctuations during these phases may be attributed to super‑spreader events or expanding testing strategy. However, a rise in Rt since April 25 may be explained by partial relaxation in mobility restrictions. - In the absence of a vaccine in the near future, a gradual buildup of herd immunity in the community due to natural infection may also aid in impeding the spread of COVID‑19. - A gradual phase‑wise lockdown exits with initiation of economic activities in nonhotspot districts may facilitate buildup of herd immunity without overwhelming health‑care facilities. However, stringent measures will have to be reintroduced when case counts start to rebound. Our estimate for herd immunity at 45% is dependent on maintaining Rt of 1.83 which was achieved at the end of extended lockdown. Rt may keep increasing when the lockdown is lifted, translating into a higher proportion requirement to be recovered from COVID‑19 to achieve herd immunity. It should be deliberated extremely cautiously considering critical care demand and fatalities, nature and length of immunity developed after infections, and the possibility of viral mutations. | |
| changes in diagnostic tests and access and any adjustment | As of April 30, 2020, India conducted 602 laboratory tests per million people and marked 586 dedicated COVID‑19 hospitals with 0.01 million isolation beds and 11,500 critical care beds. | |
| Realist | - R0, the average number of secondary infections directly generated by an infected case in a completely susceptible population, is a static yet context‑dependent indicator of transmission during an outbreak and a determinant for herd immunity threshold. - India’s health system responded to COVID‑19 pandemic with swiftness. Thus far, the fallouts of the pandemic have been less severe than countries with more robust health systems. The nationwide lockdown has shown the desired effect of slowing the epidemic. | |

1. **Association of Social Distancing, Population Density, and Temperature With the Instantaneous Reproduction Number of SARS-CoV-2 in Counties Across the United States (Rubin et al., 2020)**

| Data Collection Item | Item description / sub-groups | |
| --- | --- | --- |
| Bibiliographic Information | | |
| Author | Rubin et al. | |
| Title | Association of Social Distancing, Population Density, and Temperature With the Instantaneous Reproduction Number of SARS-CoV-2 in Counties Across the United States | |
| Year of publication | 2020 | |
| Country | USA | |
|  | | |
| Methods | | |
| Type of Study | Cohort study | |
| Time period and setting of the study | - 211 counties, representing state capitals and cities with at least 100 000 residents and including 178 892 208 US residents, in 46 states and the District of Columbia; Between February 25, 2020, and April 23, 2020. - The authors used a rolling average of the percentage of visits 3 to 14 days before time-0, based on the lag observed between changes in social distancing and mean Rt estimates across the counties and on an incubation period of at least 3 days. They considered time-0 for each county to be the date on which they achieved the minimum threshold of disease activity. | |
| Intervention | Social distancing, measured by percentage change in visits to nonessential businesses | |
| description of intervention | 10 March-15April | |
| setting of the intervention | Community of the US | |
| Aim/ Objectives | To examine the association of county-level factors with variation in the SARS-CoV-2 reproduction number over time. | |
| Participant characteristics | Participants | The 211 counties contained 178 892 208 of 326 289 971 US residents (54.8%). |
|  | Comparator details |  |
| Outcome measures | Instantaneous reproduction number (Rt) | Instantaneous reproduction number (Rt), or cases generated by each incident case at a given time, estimated from daily case incidence data. |
|  | Daily incident case | Daily incident case counts of COVID-19 aggregated at the county level |
| Source of data | - Social distancing was measured using a data set of daily cellular telephone movement, provided by Unacast, that allows comparison of the association of social distancing policies with individuals’ movement within a county. - Daily incident case counts of COVID-19 aggregated at the county level were obtained from the New York Times. - Demographic and health characteristics were abstracted from the US Census, American Community Survey, Behavioral Risk Factor Surveillance System, Esri Business Analyst, and Multi-Resolution Land Characteristics Consortium. | |
| Data analyiss | - Hierarchical linear mixed-effects model was fet with random intercepts for each county and metropolitan area to evaluate the association between exposures and Rt after a log transformation, adjusting for covariates. - The authors considered a lag period of 4 days to 14 days before case identification to reflect the incubation period of SARS-CoV-2 and to reduce bias introduced by daily weather affecting an individual’s decision to seek a test. | |
|  | | |
| Results | | |
| Study parameters | percentage change in visits to nonessential businesses (eg, restaurants, hair salons) within each county compared with visits in a 4-week baseline period between February 10 and March 8, 2020;  County median (interquartile range [IQR]) population density was 1022.7 (471.2-1846.0) people per square mile;  The mean (SD) reduction in visits by people to nonessential business by mid-April was 68.7% (7.9%);  Median (IQR) daily wet-bulb temperatures were 7.5 (3.8-12.8) °C. | |
| outcomes (Mean values for the main categories of estimated outcomes of interest, as well as mean differences between the comparator groups.) | - The mean (SD) reduction in visits by people to nonessential business by mid-April was 68.7% (7.9%). - Median (IQR) daily wet-bulb temperatures were 7.5 (3.8-12.8) °C. - The 21 counties in the top decile for population density had the highest median (IQR) incident case and fatality rate per 100 000 people (1185.2 [313.2-1891.2] cases; 43.7 [10.4-106.7] deaths), nearly 10 times the estimates in the lowest quartile (121.4 [87.8-175.4] cases; 4.2 (1.9-8.0) deaths). - Mean (SD) R in the first 2 weeks was 5.7 (2.5) in the top decile compared with 3.1 (1.2) in the lowest quartile. - The mean (SD) change in visits to nonessential businesses from April 6 to April 19 was higher among counties in the top decile (−77.9% [7.9%]) compared with those in the lowest quartile (−66.3% [7.8%]). - The top decile of counties also experienced colder median (IQR) temperatures during the analysis period compared with the counties in the lowest quartile (5.9 [3.4-8.4] °C vs 7.9 [3.1-12.7] °C). - Adjusting for county level covariates, social distancing, population density, and temperature were associated with Rt. The estimated Rt in the context of a 50% decrease in visits to nonessential businesses was 54%(95%CI, 51%-57%; P < .001) of the Rt in the setting of normal visit intensity, corresponding to a 46%decrease in the overall Rt. Compared with counties in the bottom quartile of population density, the 21 counties in the top decile of density had a 15%increase (95%CI, 9%-22%; P < .001) in relative Rt. | |
| Characterising heterogeneity | Percentage change in visits to nonessential businesses; population density; and daily wet-bulb temperatures. Change in social distancing, population density, and wet-bulb daily temperature were associated with the rate of SARS-CoV-2 transmission within a county, as measured by estimated Rt. Our analysis indicates that of these 3 factors, implementation of social distancing has been the most significant in reducing transmission. In addition, the mitigating association of increased social distancing and moderate increases in wet-bulb daily temperature were most dramatic in counties with higher population density, which had high Rt values. | |
|  | | |
| Discussion | | |
| Study findings, (Summarise key study findings and describe how they support the conclusions reached.) | The results of this study suggest that social distancing, population density, and daily weather may account for variation in the Rt for SARS-COv-2 across the United States. These results may guide policy decisions for managing this pandemic more selectively at the local level throughout the country. | |
| limitations | - First, the 45% of US residents not captured in the analysis were residing in smaller, rural counties, and as such, this study's models are not applicable to these areas. - Second, temperature associations observed might have been confounded by time period in the analysis, given that outbreaks occurred during spring in parallel with changing weather. - Third, increases in testing capacity might have biased the models by inflating the total cases reported within each county. - Fourth, as the random county and metropolitan area intercepts explained additional variation, there are likely other unmeasured county factors that the authors did not capture. | |
| generalisability | Generalizability remains a concern, particularly given the focus of this study on larger counties. The 45% of US residents not captured in the analysis were residing in smaller, rural counties, and as such, our models are not applicable to these areas. | |
| current knowledge (how the findings fit with current knowledge.) | County-level analysis has allowed the authors to better examine relevant contributions of social distancing, population density, and seasonal weather changes on a given county’s Rt. This approach gives valuable information on risk of transmission to inform area-specific public policy decisions. It will be important to examine whether the introduction of these associations to models can accurately estimate the likelihood of viral transmission in the future, given that these factors will continue to change. | |
| changes in diagnostic tests and access and any adjustment | It is possible that differences in diagnostic test availability could contribute to the variation detected by the random effects across counties. | |

1. **Effectiveness of the measures to flatten the epidemic curve of COVID-19. The case of Spain (Saez et al., 2020)**

| Data Collection Item | Item description / sub-groups | |
| --- | --- | --- |
| Bibiliographic Information | | |
| Author | Saez, et al. | |
| Title | Effectiveness of the measures to flatten the epidemic curve of COVID-19. The case of Spain | |
| Year of publication | 2020 | |
| Country | Spain | |
|  | | |
| Methods | | |
| Type of Study | Time series design, | |
| Time period and setting of the study | Time series design, using information from January 17 to April 5, 2020. | |
| Intervention | Physical distancing measures | |
| description of intervention | Physical distancing measures on were started on March 14 (two weeks), 13 days after the epidemic outbreak started its exponential growth. | |
| setting of the intervention | Spain community | |
| Aim/ Objectives | To evaluate ex-ante (before the flattening of the curve) the effectiveness of the measures adopted by the Spanish Government to mitigate the COVID-19 epidemic. | |
| Participant characteristics | Participants | The whole Spain country |
|  | Comparator details |  |
| Outcome measures | Accumulated cases |  |
| Source of data | The data source was the European Center for Disease Prevention and Control (ECDC, 2020c). | |
| Data analyiss | - Two generalized linear mixed models (GLMM) with variable response from the Gaussian family (i.e. linear mixed models) were used. - All analyses were made with the free statistical environment R (version 3.6.3) (R Core Team, 2020). | |
|  | | |
| Results | | |
| Study parameters | Percentage change of daily variation rate of accumulated cases; | |
| outcomes (Mean values for the main categories of estimated outcomes of interest, as well as mean differences between the comparator groups.) | - As of March 15, the variation rate of accumulated cases decreased daily, on average, by 3.059 percentage points, (95% credibility interval: −5.371, −0.879). The reduction, when comparing before and after the intervention (calculated as reduction in delay 1 minus the counterfactual), was, on average, 5.186 percentage points each day. - The reduction in the rate of variation of the accumulated cases increased from the day after the intervention (March 15), specifically from −0.32 percentage points to −5.11 percentage points. | |
| Characterising heterogeneity | - | |
| - | | |
| Discussion | | |
| Study findings, (Summarise key study findings and describe how they support the conclusions reached.) | - Just one day after implementing the measures, the variation rate of accumulated cases decreased daily, on average, by 3.059 percentage points. This reduction will be greater as time passes. - The reduction in the variation rate of the accumulated cases, on the last day for which data were available, has reached 5.11 percentage points. The measures taken by the Spanish Government on March 14, 2020 to mitigate the epidemic curve of COVID-19 managed to flatten the curve and although they have not (yet) managed to enter the decrease phase, they are on the way to do so. | |
| limitations | - First, under-registration occurs in Spain as a consequence of a lack of tests. It is very possible that when mass screening is carried out, the number of confirmed cases will increase, each time approaching the real incidence. - Second, the model performs well in the short term (a week at most), limiting it in making predictions about when the epidemic will peak. | |
| generalisability |  | |
| current knowledge (how the findings fit with current knowledge.) | - The epidemic curve of new cases is not comparable between countries, since there is great variability in the diagnosis of the disease. Nevertheless, the epidemic curve of the accumulated cases shows striking similarity between countries, especially once they are put on the same scale, defining day 0 as the outbreak of the epidemic. | |
| changes in diagnostic tests and access and any adjustment | Under-registration occurs in Spain as a consequence of a lack of tests. It is very possible that when mass screening is carried out, the number of confirmed cases will increase. However, the authors believe that the results on the variation of accumulated cases are much more reliable, since they are not based on the number of cases but on the shape of the epidemic curve, which is very similar in all the countries. | |

1. **Social distancing to slow the US COVID-19 epidemic: Longitudinal pretest–posttest comparison group study (Siedner et al., 2020)**

| Data Collection Item | Item description / sub-groups | |
| --- | --- | --- |
| Bibiliographic Information | | |
| Author | Siedner et al. | |
| Title | Social distancing to slow the US COVID-19 epidemic: Longitudinal pretest–posttest comparison group study | |
| Year of publication | 2020 | |
| Country | USA | |
|  | | |
| Methods | | |
| Type of Study | Ecological analysis, longitudinal pretest–posttest comparison group study | |
| Time period and setting of the study | All 50 states and the District of Columbia; Between January 21 and May 1, 2020   - The primary exposure was time before (14 days prior to, and through 3 days after) versus after (beginning 4 days after, to up to 21 days after) implementation of the first statewide social distancing measures. - The authors selected 4 days after implementation as the transition point based on previously published estimates of the lower end of the 95% confidence interval (CI) of the COVID-19 incubation period, which is when cases at a population level should be expected to decline in the setting of a structural intervention. - The authors limited the analysis to 21 days after implementation to prevent any potential diluting effects resulting from relaxation of some social distancing measures, which in some states began less than 4 weeks after implementation (e.g., Alabama, Alaska, Mississippi, and South Carolina). | |
| Intervention | Social distancing | |
| description of intervention | - Closures of schools, closures of workplaces, cancellations of public events, restrictions on internal movement, and closures of state borders. Restrictions on internal movement, i.e., shelterinplace orders (often referred to colloquially as “lockdowns”), are generally the most restrictive of these in terms of their impacts on daily movement. - All states initiated social distancing measures between March 10 and March 25, 2020. - State-wide social distancing measures implemented between January 21 and March 30, 2020 | |
| setting of the intervention | All states initiated social distancing measures between March 10 and March 25, 2020. | |
| Aim/ Objectives | To estimate the change in COVID-19 case growth before versus after implementation of statewide social distancing measures in the US. | |
| Participant characteristics | Participants | Population of all 50 states and the District of Columbia |
|  | Comparator details |  |
| Outcome measures | COVID-19 case growth rate | Rate of change in daily COVID-19 cases in each state, calculated as the natural log of cases on each date minus the natural log of cases on the prior date. |
|  | COVID-19-attributed mortality  growth rate | Change in daily COVID-19-attributed deaths in each state, calculated as the natural log of deaths on each date minus the natural log of deaths on the prior date. |
| Source of data | - The New York Times COVID-19 database was used to obtain daily state-specific reported COVID-19 cases and deaths, (https://github.com/nytimes/covid-19-data; last accessed May 26, 2020). - Reporting of cases and deaths in the New York Times database varies by state, but typically includes both laboratory-confirmed and suspected cases, as recommended by the Council of State and Territorial Epidemiologists. | |
| Data analyiss | - Analysis was restricted to days on which a state had at least 30 cumulative cases reported, to minimize any effects of volatile rate changes early in the epidemic. - Mixed effects linear regression models, specifying the log difference in daily cases as the outcome of interest and including a random effect for state, was fitted to allow for within-state correlation of cases over time. | |
|  | | |
| Results | | |
| Study parameters | Median statewide epidemic size (interquartile range [IQR]);  Mean daily case growth rate (95% CI);  Percentage of changes in daily COVID-19-attributed deaths (95% CI). | |
| outcomes (Mean values for the main categories of estimated outcomes of interest, as well as mean differences between the comparator groups.) | - At the date of implementation of the first social distancing measure, states had a mean daily case growth rate of 30.8% (95% CI 29.1–32.6), corresponding to a doubling of total cases every 3.3 days. - From 14 days prior to, and through 3 days after, implementation of the first social distancing measure, the mean daily case growth rate did not change (−0.2% per day; 95% CI −0.6% to 0.3%; P = 0.51). - Beginning 4 days after implementation of the first statewide social distancing measure, the mean daily case growth rate decreased by an additional 0.9% per day (95% CI −0.4% to −1.4%; P < 0.001). This estimate corresponds to a mean daily case growth rate that had declined to 26.5% (doubling of total cases every 3.8 days) by day 7 after enactment of the first statewide social distancing measures, to 19.6% (doubling time of 5.1 days) by day 14, and to 12.7% (doubling time of 7.9 days) by day 21. - The mean daily case growth rate was already declining, at a mean rate of −0.8% per day, during the 14 days prior to implementation of statewide restrictions on internal movement (95% CI −0.9% to −0.7%; P < 0.001). There was a drop detected 3 days after statewide restrictions on internal movement were implemented (−3.1%; 95% CI −4.7% to −1.5%; P <0.001). - By 7 days after implementation of the first statewide social distancing measure, the mean daily growth rate in COVID-19-attributed deaths decreased by 2.0% per day (95% CI −3.0% to −0.9%; *P <* 0.001). By 14 days, the estimated association was no longer statistically significant (−1.0% per day; 95% CI −0.2% to 0.1%; *P =* 0.09). | |
| Characterising heterogeneity |  | |
|  | | |
| Discussion | | |
| Study findings, (Summarise key study findings and describe how they support the conclusions reached.) | - The case growth rate declined by approximately 1% per day beginning 4 days (approximately 1 incubation period) after statewide social distancing measures were implemented. - The model implies that social distancing reduced the total number of COVID-19 cases by approximately 1,600 reported cases at 7 days after implementation, by approximately 55,000 reported cases at 14 days after implementation, and by approximately 600,000 reported cases at 21 days after implementation. | |
| limitations | - The estimates would be biased toward the null if (1) state and local governments intensified social distancing measures in response to a worsening epidemic, (2) there were substantial violations of the stable unit treatment value assumption (e.g., workplace closures of large employers that had spillover effects across state lines), or (3) surveillance and testing intensified during the study period (thereby resulting in increased case reporting). - Statewide restrictions on internal movement were often implemented after other social distancing measures had already been applied, further biasing the estimate toward the null. - Estimates of cases and deaths in the model include both those that are laboratory-confirmed and those that are suspected by health departments, but both are likely to be underestimates due to limitations in testing, the presence of asymptomatic cases, and the occurrence of COVID-19-related deaths that are not attributed to COVID-19. - Finally, at the time of this analysis, states had begun to relax social distancing measures, which necessarily prevents drawing conclusions about the long-term associational effects of these interventions in isolation. | |
| generalisability |  | |
| current knowledge (how the findings fit with current knowledge.) | - The analysis cannot answer questions about the appropriate time for rescinding social distancing measures. While the findings demonstrate that early mitigation efforts have yielded a substantial population health benefit, such benefits should be weighed against their costs. The costs of social distancing are likely to exacerbate the confluence of longstanding economic, social, and health decline that is already occurring in the US, brought into even sharper relief given emerging data about racial, ethnic, and socioeconomic disparities in the incidence of COVID-19-related burden. - The finding that implementation of statewide social distancing was associated with a reduction in the mean daily growth rate of COVID-19-attributed deaths should be interpreted with more caution, given the uncertainty in published estimates of the median time from symptom onset to death. The strongest association the authors estimated, in terms of both statistical significance and magnitude, occurred at 7 days after implementation. While this association could be reflective of what is known about time to death for the median hospitalized patient, other plausible lag times were not associated with a statistically significant reduction in the mean daily growth rate of COVID-19-attributed deaths. | |
| changes in diagnostic tests and access and any adjustment | Surveillance and testing intensified during the study period (thereby resulting in increased case reporting. Estimates of cases and deaths in the model include both those that are laboratory-confirmed and those that are suspected by health departments, but both were likely to be underestimates due to limitations in testing, the presence of asymptomatic cases, and the occurrence of COVID-19-related deaths that are not attributed to COVID-19. Nonetheless, the analyses focused on day-to-day changes in the growth rate of cases and deaths, so underreporting would only have biased the results if reported versus true outcomes systematically differed prior to versus after the implementation of social distancing measures. In contrast, the projected estimates of cases prevented were likely to be highly conservative, because they were modeled based on reported cases. | |

1. **The effect of lockdown on the COVID-19 epidemic in Brazil: evidence from an interrupted time series design(Silva et al., 2020)**

| Data Collection Item | Item description / sub-groups | |
| --- | --- | --- |
| Bibiliographic Information | | |
| Author | Silva, L. et al. | |
| Title | The effect of lockdown on the COVID-19 epidemic in Brazil: evidence from an interrupted time series design | |
| Year of publication | 2020 | |
| Country | Brazil | |
|  | | |
| Methods | | |
| Type of Study | Interrupted Time Series Analysis | |
| Time period and setting of the study | - All the Brazilian state capitals that officially adopted more restrictive policies; June 25th - Following previous literature on the effects of lockdown measures on the COVID 19 pandemic, the authors adopted a two-week observation window after the end lockdown policies to test the series interruption 4,5,6,7. | |
| Intervention | Social distancing | |
| description of intervention | - São Luís (Maranhão State): 1,101,884 population, from May 5th to May 17th, 13 days, Post-intervention window: Until May 31 - Recife (Pernambuco State): 1,645,727 population, from May 16th to May 31st, 16 days, Post-intervention window: Until June 14. - Belém (Pará State): 1,492,745 population, from May 7th to May 24th, 18 days, Post-intervention window: Until June 7. - Fortaleza (Ceará State): 2,669,342 population, from May 8th to May 30th, 23 days, Post-intervention window: Until June 13 | |
| setting of the intervention | Maranhão State, Pernambuco State, Pará State, Ceará State at Brazil. | |
| Aim/ Objectives | To estimate the impact of social distance measures on reducing both the number of cases and deaths due to COVID-19 in Brazil. | |
| Participant characteristics | Participants | all the Brazilian state capitals that officially adopted more restrictive policies (i.e. “lockdown”) in response to the SARS-CoV-2 epidemic: Belém (Pará State) (1,492,745 population), Fortaleza (Ceará State) (2,669,342 population), Recife (Pernambuco State) (1,645,727 population), and São Luís (Maranhão State) (1,101,884 population). |
|  | Comparator details |  |
| Outcome measures | Daily new cases |  |
|  | Deaths |  |
| Source of data | - On June 25th, data were collected from the Brazilian Ministry of Health website, which provides daily disaggregated information on COVID-19 (<https://covid.saude.gov.br/>). | |
| Data analyiss | - Official daily data was collected for four city capitals before and after their respective policies interventions based on a 14 days observation window. The authors estimated a segmented linear regression to evaluate the effectiveness of lockdown measures on COVID-19 incidence and mortality. - The data was analyzed using R Statistical 3.6.3 (http://www.r-project.org), and all significance tests were two-sided. Replication materials, including raw data and computational scripts, are available at: https://osf.io/ns3k6/. | |
|  | | |
| Results | | |
| Study parameters | Daily new case trend;  Mortality trend. | |
| outcomes (Mean values for the main categories of estimated outcomes of interest, as well as mean differences between the comparator groups.) | - Before the policy change, the trend (new daily case) was positive and statistically significant for all units. Controlling for prior trend, we observed statistically significant decreases in São Luís (β3 = -0.09; p-value < 0.001), Recife (β3 = -0.12; p-value < 0.001), Belém (β3 = -0.13; p-value < 0.001), and Fortaleza (β3 = -0.07; p-value < 0.001). - The mortality trend before the intervention was also positive for all cities, meaning an increasing number of deaths over time, showing a statistically significant slope change after lockdown. All coefficients for the post-intervention period were negative, as hypothesized: São Luís (β3 = -0.13; p-value < 0.001), Recife (β3 = -0.06; p-value < 0.001), Belém (β3 = -0.10; p-value < 0.001) and Fortaleza (β3 = -0.09; p-value < 0.001). | |
| Characterising heterogeneity | - | |
|  | | |
| Discussion | | |
| Study findings, (Summarise key study findings and describe how they support the conclusions reached.) | - The initial number of new cases and new deaths had a positive trend prior to policy change. After lockdown, a statistically significant decrease in new confirmed cases was found in all state capitals. - The authors also found evidence that lockdown measures were likely to reverse the trend of new daily deaths due to COVID-19. - In São Luís, the authors observed a reduction of 37.85% while in Fortaleza the decrease was 33.4% on the average difference in daily deaths if the lockdown had not been implemented. Similarly, the intervention diminished mortality in Recife by 21.76% and Belém by 16.77%. Social distancing policies can be useful tools in flattening the epidemic curve. | |
| limitations | - First, Brazilian official data shows some inconsistencies that may affect estimates efficiency; thus, studies working with small samples could fail to reject the null hypothesis due to micronumerosity. - Second, testing intensified during the post-intervention period, resulting in a higher number of diagnosed cases that can lead to underestimating policy change impact. - Finally, there is significant delay between testing cases and actual report them in official datasets, which are likely to introduce both random and systematic measurement errors in statistical estimates. | |
| generalisability | - | |
| current knowledge (how the findings fit with current knowledge.) | Studies using ITS design consistently reports significant effects of social distancing policies on the SARS-CoV-2 epidemic. | |
| changes in diagnostic tests and access and any adjustment | Testing intensified during the post-intervention period, resulting in a higher number of diagnosed cases that can lead to underestimating policy change impact. | |

1. **Evaluation of the lockdowns for the SARS-CoV-2 epidemic in Italy and Spain after one month follow up (Tobias, 2020)**

| Data Collection Item | Item description / sub-groups | |
| --- | --- | --- |
| Bibiliographic Information | | |
| Author | Tobías et al. | |
| Title | Evaluation of the lockdowns for the SARS-CoV-2 epidemic in Italy and Spain after one month follow up | |
| Year of publication | 2020 | |
| Country | Italy and Spain | |
|  | | |
| Methods | | |
| Type of Study | Interrupted Time-Series Design | |
| Time period and setting of the study | Data were collected between February 24th and April 5th in Italy and Spain before and during their respective national lockdowns | |
| Intervention | National lockdowns social distancing measures. | |
| description of intervention | - In Italy, restricting social contact, closure of businesses, with the exception of those essential to the country's supply chains. - In Spain, reducing the mobility and non-essential industrial activity countrywide. - Since March 8th, widespread lockdown measures have been in place in Italy. Specific measures restricting social contact were first introduced in the northern regions, where most cases had occurred, then extended to the whole country on March 10th. Italy tightened these measures extending the lockdown on March 21st: all businesses were closed, with the exception of those essential to the country's supply chains (first lockdown March 8th and second lockdown March 10th). - Similarly, Spain imposed a lockdown on March 16th, with social distancing measures similar to those established in Italy. Two weeks later, on March 30th, Spain also implemented a more restrictive lockdown, aimed at reducing the mobility and non-essential industrial activity countrywide (first lockdown March 16th and second lockdown March 30th) | |
| setting of the intervention | Community of Italy and Spain | |
| Aim/ Objectives | To quantify, and compare the lockdown effects within and between countries from an epidemiological point of view using incident data. | |
| Participant characteristics | Intervention group | Italy with 60,461,826 people and Spain with 46,754,778 people |
|  | Comparator details |  |
| Outcome measures | Incidence | Daily diagnosed cases |
|  | Daily deaths | Number |
|  | Daily ICU admissions | Number |
|  |  |  |
| Source of data | Websites of the Italian and Spanish Ministries of Health (Dipartamento della Protezione Civille, 2020; Instituto de Salud Carlos III, 2020). | |
| Data analyiss | - Data was analyzed using quasi-Poisson regression using an interaction model to estimate the change in trends. - The data was analyzed using Stata, release 16 (StataCorp, College Station, TX, 2019). | |
|  | | |
| Results | | |
| Study parameters | Daily percent increase (%IR) of diagnosed cases, deaths and ICU admission | |
| outcomes (Mean values for the main categories of estimated outcomes of interest, as well as mean differences between the comparator groups.) | - Before the lockdown, the daily percent increase of all the incidence outcomes was higher in Spain (38.5% for diagnosed cases, 59.3% for deaths, and 26.5% for ICU admissions) than in Italy (21.6%, 32.8%, and 16.7%, respectively) - During the first lockdown period, both countries show similar daily trends (12.5%, 13.7%, and 3.7% in Italy; and 11.9%, 17.6%, and 9.6% in Spain). Thus, during the first lockdown the daily increase in incident data was considerably reduced. In Italy, the diagnosed cases decreased by 42.1%, deaths by 58.2%, and ICU admissions by 77.8%. This reduction was even higher in Spain, where the diagnosed cases decreased by 69.1%, deaths by 77.8%, and ICU admissions by 66.8%. However, although the slopes have been flattened for all outcomes, the trends kept rising. - During the second and more restrictive lockdown, in Italy all outcomes start declining; diagnosed cases if −2.0%, daily deaths of −0.2%, and ICU admissions of −16.8%. Similarly, Spain also shows decline trends in daily diagnosed cases of −2.7%, deaths of −1.8%, and ICU of −5.6%. | |
| Characterising heterogeneity | - | |
|  | | |
| Discussion | | |
| Study findings, (Summarise key study findings and describe how they support the conclusions reached.) | - Although lockdown, including restricted social contact and keeping open only those businesses essential to the country's supply chains, had considerably reduced the trend slopes for all the outcomes in both countries, this was not enough to change the rising trend of the epidemic. Therefore, more restrictive actions were suggested. - The second lockdown, shows how the trends have changed, with a reduction of daily incident cases, deaths, and more significantly in ICUs. These are of similar magnitude in both countries, although Italy carries a week ahead of Spain. However, mortality still shows a small increase, probably because it follows incidence trends with a delay of 1–2 weeks. | |
| limitations | The changes in the definition of diagnosed cases have not been taken into account, nor has the reduction in the susceptible population because of the lockdown. | |
| generalisability |  | |
| current knowledge (how the findings fit with current knowledge.) | - Timely indications for public health authorities and governments are essential to slow down the SARS-CoV-2 epidemic and relieve the pressure on overburdened health services. - The positive signs already shown by the decreasing trend slopes after a more restrictive lockdown in Italy and Spain could indicate an optimistic and encouraging forecast for those countries that in late March also announced restrictive lockdown measures for flattening the SARSCoV-2 epidemic curve (e.g., the United Kingdom on March 23th or Ireland on March 27th). These results show that the sacrifices that our society is making are gaining us valuable time, which is essential to get ready to face the future pressures that this epidemic will bring forth. | |
| changes in diagnostic tests and access and any adjustment | The changes in the definition of diagnosed cases have not been taken into account, nor has the reduction in the susceptible population because of the lockdown. Therefore, the incident cases were modeled directly instead of the incidence rate, assuming that the entire population was at risk. | |

1. **Associations of Stay-at-Home Order and Face-Masking Recommendation with Trends in Daily New Cases and Deaths of Laboratory-Confirmed COVID-19 in the United States(Xu et al., 2020)**

| Data Collection Item | Item description / sub-groups | |
| --- | --- | --- |
| Bibiliographic Information | | |
| Author | Xu et al. | |
| Title | Associations of Stay-at-Home Order and Face-Masking Recommendation with Trends in Daily New Cases and Deaths of Laboratory-Confirmed COVID-19 in the United States | |
| Year of publication | 2020 | |
| Country | USA | |
|  | | |
| Methods | | |
| Type of Study | Quasi-experimental interrupted time-series study | |
| Time period and setting of the study | Between March 1 and April 20, 2020, in the 50 states and DC. | |
| Intervention | Stay-at-home order (SAHO) and face-masking recommendation | |
| description of intervention | - On March 19, 2020, the state of California started a SAHO which affected 39,512,223 residents (12.0% of the U.S. population). The number and proportion of residents under the SAHO subsequently implemented by other 39 states and DC continued to increase until April 7, and plateaued at 290,829,980 and 88.6%, respectively. - Approximately 85.2% of the U.S. population were under SAHO on April 3, 2020, while only 3.4% of the U.S. population were added from April 3 to April 25. The number of the states that implemented a state-wide SAHO peaked at 41 on April 25, 2020, and some states started to lift the state-wide SAHO on April 26, 2020 - The Centers for Disease Control and Prevention (CDC) also recommended face-masking on April 3, 2020.7 | |
| setting of the intervention | Community | |
| Aim/ Objectives | To compare the changes in COVID-19 epidemics before and after SAHO and face-masking recommendation. | |
| Participant characteristics | Participants | The population under SAHO in the USA grew from March 19, 2020 to April 6, 2020, and reached 290,829,980 (88.6%) by April 7. |
|  | Comparator details |  |
| Outcome measures | Daily new  cases | Defined as laboratory-confirmed positive cases |
|  | Deaths of laboratory-confirmed COVID-19 cases | Deaths which were reported by a state’s public health authority for better data consistency and quality |
|  | COVID-19 time-varying reproduction numbers (RT) | The mean number of secondary cases generated by a typical primary case at the time (t) in a population. |
| Source of data | - To adjust for confounders, the proportions of daily positive results in all daily tests, and state-level daily new cases and deaths were obtained from the COVID-19 Tracking Project. - The state populations were extracted from the USA Census (up to July 2019). | |
| Data analyiss | - Multivariable piecewise log-linear regression was used to examine the associations. - Several population-based factors were included in the multivariable piecewise log-linear regression analyses. - All statistical analyses were performed using Stata (version 15) and the Joinpoint program (NCI, version 4.7.0.0) with the Poisson variance option. All p-values were two-sided, with a cut-off of 0.05 for significance. - COVID-19 time-varying reproduction numbers were estimated using previously-reported mean serial-intervals and the R package (version 3.6.3). | |
|  | | |
| Results | | |
| Study parameters | COVID-19 daily cases trend;  Daily new deaths trend. | |
| outcomes (Mean values for the main categories of estimated outcomes of interest, as well as mean differences between the comparator groups.) | - The number of states under SAHO [coefficient = 0.03, 95% confidence interval (CI) of −0.01 to 0.07, p = 0.15], the population under SAHO (coefficient of log10(population under SAHO) = −0.13, 95% CI of −0.88 to 0.63, p = 0.74; coefficient of population under SAHO = 0.00, 95% CI of 0.00 to 0.00, p = 0.78), population-proportion under SAHO (coefficient = −0.16, 95% CI of −1.37 to 1.05, p = 0.79) and the proportion of positive tests in the USA (coefficient = −0.24, 95% CI od −0.73 to 0.24, p = 0.32) were not linked to the daily new cases or deaths. - The trend in COVID-19 daily cases reduced after March 23 (slope changes = −0.18, 95% CI of −0.22 to −0.14, p< 0.001) and further reduced on April 3 (slope change = −0.10, 95% CI of −0.18 to −0.08, p < 0.001), which appeared associated with the implementation of SAHO by 10 states (34.7% of the U.S. population) on March 23, and the CDC’s recommendation of face masking on April 3, respectively. - The overall slope change of daily new deaths attributable to SAHO and face-masking were −0.17 (95% CI of −0.21 to −0.14) and −0.13 (95% CI of −0.25 to −0.07), respectively. - New York state and its neighbor states had higher daily new cases and deaths than other states. The multivariable piecewise log-linear regression also revealed the state’s neighboring relationship with New York was linked to COVID-19 daily new cases (coefficient = −1.43, 95% CI of −1.57 to −1.28) and new deaths (coefficient = −1.42, 95% CI of −1.57 to −1.26), whereas the two turning points were March 28 (slope changes = −0.09, 95% CI of −0.17 to −0.01) and April 3 (slope changes = −0.09, 95% CI of −0.17 to −0.01) for daily new cases, and April 9 (slope changes = −0.06, 95% CI of −0.10 to −0.02) and April 19 (slope changes = −0.90, 95% CI of −0.94 to −0.86) for daily new deaths, respectively. Correspondingly, there were 159 million USA residents (48.5% of the U.S. population, 23 states) under SAHO on March 28, 279.5 million U.S. residents (85.2% of the U.S. population, 38 states) under SAHO on April 3, and 290.8 million U.S. residents (88.6% of the U.S. population, 41 states) under SAHO since April 7. - The estimates of Rt based on the three reported mean serial-intervals of COVID-19 all started to decline on March 19, when SAHO was first implemented in the USA, and declined faster after March 23 . After a short plateau, Rt continued to decline after April 3 and fell below/around 1.0 on April 13. | |
| Characterising heterogeneity | Considering the recently reported state heterogeneity of COVID- 19 epidemics in the European Union, the authors examined the association between the states’ neighboring relationship with New York (epicenter at the time) and COVID-19 epidemics (daily COVID-19 new cases or new deaths). The relationship was coded as 0 for New York, 1 for the six neighbor states (Connecticut, Massachusetts, New Jersey, Pennsylvania, Rhode Island, and Vermont), and 2 for the other states and DC. The authors compared these epidemics using Wilcoxon rank sum test and Student’s t-test. The authors then conducted sensitivity studies using multivariable piecewise log-linear regression with the states’ relationship with New York as a covariate. The COVID-19 trend lines were smoothed in the figure using a locally weighted scatter smoothing (known as LOWESS) algorithm. | |
|  | | |
| Discussion | | |
| Study findings, (Summarise key study findings and describe how they support the conclusions reached.) | - The multivariable piecewise log-linear regression models identified two turning points of COVID-19 daily new cases and the time-varying reproduction number, Rt, in the USA, as well as the link between states’ relationship with New York and COVID-19 epidemics. - The two turning points were associated with implementation of SAHO on March 28 (affecting 48.5% of USA population in 22 states and DC) and the CDC’s face-masking recommendation on April 3. Similar turning points of COVID-19 daily new deaths were April 3 and April 15, which represented 10 and 12 days of delay, respectively. | |
| limitations | - First, the COVID-19 positive rates varied among states and by time, suggesting under-testing of the potential patients. - Second, there was a lag in COVID-19 reporting, which may lead to inaccurate estimation of the case numbers. However, the increases in the proportion of the tested population appeared stable in the USA, and was not a factor linked to COVID-19 epidemics in the multivariable models. It suggests that the lag in reporting may not change significantly as the time changes, and will have minimal impact on the trend analyses. - Third, the CDC changed the diagnostic criteria of COVID-19 on April 14, 2020. The timing and extent of these changes in each state were largely unknown, while the authors consider the data released before April 20 acceptable owing to the transition time of different criteria. Therefore, the data released after April 20 would have an unmeasurable bias and would not be suitable for comparing with those before April 20. Thus, the authors were not able to reliably compare the observed and the simulated COVID-19 trends after April 20. - Finally, the authors did not report the daily incidence. There were no significant changes in the USA population during the study-period. The daily new cases of COVID-19 thus should be proportional to its daily incidence in the USA but are easier to interpret than daily incidence and were used here. | |
| generalisability | The socioeconomic systems and the modalities and extents of public health interventions in the USA has been different from other countries which might decrease the generalizability of the study to other countries. | |
| current knowledge (how the findings fit with current knowledge.) | - Although Rt is one of the most widely used metrics for assessing transmission rate of infectious diseases and linked to the incidence decay with exponential adjustment model (known as IDEA) and Farr’s law, it is difficult to estimate. One of the challenges is the variances in the SARS-CoV2 serial intervals. The reported serial intervals of COVID-19 ranged from 3.96 to 7.5 days, and were first used to rigorously examine the changes of Rt associated with SAHO and face-masking recommendation in the USA. - The states’ neighboring relationship with the epicenter (New York) in the USA was linked to the COVID-19 epidemics in our multivariable models, while the number of states under SAHO, proportion of positive COVID-19 testing and the number and proportion of the populations under SAHO were not linked to them. Additional works are needed to better understand the geographic differences in state-level COVID-19 trends. - the simulation analysis shows early-implementation of SAHO would be associated with a significant reduction in daily new cases and deaths, while lift of SAHO would be associated with a significant increase in daily new cases and deaths | |
| changes in diagnostic tests and access and any adjustment | - The COVID-19 positive rates varied among states and by time, suggesting under-testing of the potential patients. The exact COVID-19 case numbers were thus not available, although efforts were made to estimate them using the Johns Hopkins’ data repository of COVID-19 cases. Given the data inconsistence the authors noticed, such an estimation was not optimal in their view. The authors were more confident in the reliability of the laboratory-confirmed case numbers. Inclusion of positive-test rate in the models may have alleviated the variances in test rate across time. Indeed, the positive-test rate was not a factor linked to COVID-19 epidemics in the multivariable models. - Second, there was a lag in COVID-19 reporting, which may lead to inaccurate estimation of the case numbers. However, the increases in the proportion of the tested population appeared stable in the USA, and was not a factor linked to COVID-19 epidemics in the multivariable models. | |

1. **Social distancing in São Paulo State: demonstrating the reduction in cases using time series analysis of deaths due to COVID-19(Cruz, 2020)**

| Data Collection Item | Item description / sub-groups | |
| --- | --- | --- |
| Bibiliographic Information | | |
| Author | Cruz et al. | |
| Title | Social distancing in São Paulo State: demonstrating the reduction in cases using time series analysis of deaths due to COVID-19 | |
| Year of publication | 2020 | |
| Country | Brazil | |
|  | | |
| Methods | | |
| Type of Study | Time series | |
| Time period and setting of the study | Social Distancing measures adopted by São Paulo State between March 16th and March 22nd. | |
| Intervention | Social Distancing Strategies | |
| description of intervention | In this period, state decrees were issued, establishing a system of work-from-home and the mandatory closure of non-emergency businesses and educational institutions throughout the state. | |
| setting of the intervention | São Paulo State and in Brazil | |
| Aim/ Objectives | We use a time series analysis of deaths due to COVID-19 referenced to the date of deaths (as opposed to the date in which the test results were obtained) | |
| Participant characteristics | Participants | COVID-19-suspected deaths, attributed by a certified physician. |
|  | Comparator details | Deaths attributed directly to COVID (suspected and confirmed) plus the excess deaths attributed by the ARPEN data to Severe Acute Respiratory Syndrome (SARS) in 2020 with respect to 2019 (deaths in 2020 minus the deaths in 2019, which we consider a baseline). |
| Outcome measures |  | Deaths due to COVID-19 on a daily basis |
|  |  | Death Growth rate (additional deaths per day) |
| Source of data | National Association of the Registrars for Natural Persons (Associação dos Registradores de Pessoas Naturais - ARPEN) | |
| Data analyiss | Descriptive statistics | |
|  | | |
| Results | | |
| Study parameters | Deaths due to COVID-19 on a daily basis and Death Growth rate (additional deaths per day) number | |
| outcomes (Mean values for the main categories of estimated outcomes of interest, as well as mean differences between the comparator groups.) | - The authors hypothesize that the abrupt change in the rising trend observed around April 5th can be attributed to the Social Distancing measures adopted by São Paulo State between March 16th and March 22nd. - It appears that at a SDI between 52 and 56%, makes the progression of the disease to cross the break-even point: an SDI of 56% causes decrease deaths 0.40 per day, whereas an SDI of 52% increase deaths to 0.82 per day. For the normal-life period (before March 16th) the disease progressed at a rate of 3.18 deaths per day. | |
| Characterising heterogeneity | São Paulo State vs São Paulo City. | |
|  | | |
| Discussion | | |
| Study findings, (Summarise key study findings and describe how they support the conclusions reached.) | - The data shown above demonstrate that SDS can change the course of the spread of disease. - Using a time series analysis referenced to the date of death, and not to the date of testing for COVID-19, we demonstrated that the Social Distancing Strategy adopted in São Paulo State brought substantial results in terms of reducing the number of infections. | |
| limitations | - A limitation in the approach, if it is to be used for fast response decisions, is the delay which exists between the death and the emission of the Death Certificate and its filing in the Civil Registry database, which can be as long as 15 days. | |
| generalisability | - | |
| current knowledge (how the findings fit with current knowledge.) | A gross estimate, using the data for São Paulo City, indicated that a Social Distancing Index (as defined and informed by the State Government) higher than 55% may be needed to reduce the number of deaths. | |
| changes in diagnostic tests and access and any adjustment | - | |

1. **The effect of state-level stay-at-home orders on COVID-19 infection rates(Castillo et al., 2020)**

| Data Collection Item | Item description / sub-groups | |
| --- | --- | --- |
| Bibiliographic Information | | |
| Author | Castillo et al. | |
| Title | The effect of state-level stay-at-home orders on COVID-19 infection rates | |
| Year of publication | 2020 | |
| Country | USA | |
|  | | |
| Methods | | |
| Type of Study | Observational | |
| Time period and setting of the study | 42 states and the District of Columbia; March- April | |
| Intervention | stay-at-home orders | |
| description of intervention | - Stay-at-home orders were defined as statewide mandates that included a) mandatory nonessential business closures, b) furloughs enforced for most government and commercial employees, c) prohibition of public events and gatherings, and d) travel restrictions including orders to avoid leaving the home except for necessities such as groceries and medical care. - Forty-two states and the District of Columbia were identified to have issued a stay-at-home order between March 19, 2020 and April 7, 2020. | |
| setting of the intervention | 42 states and the District of Columbia with stay-at-home orders | |
| Aim/ Objectives | examine the effect of stay-at-home orders on the rate of increase in COVID-19 diagnoses | |
| Participant characteristics | Participants | 42 states and the District of Columbia |
|  | Comparator details |  |
| Outcome measures | Infection Rates | Daily confirmed COVID-19 cases |
| Source of data | - Google searches were conducted daily to assess state-level responses to the COVID-19 pandemic. - Daily confirmed COVID-19 cases were obtained from the Johns Hopkins Center for Health Security Application and downloaded via GitHub. | |
| Data analyiss | - Linear regression techniques were used to determine slopes for log case count data, and meta analyses were conducted to combine data across states. - Meta-analytic techniques were used to combine data across states using the METAN command suite in Stata. - A sensitivity analysis was conducted using the same linear regression and meta-analytic approach to determine whether delaying the days counted as post-order by one week would impact the infection rates and R2 fit statistics. | |
|  | | |
| Results | | |
| Study parameters | Daily confirmed COVID-19 cases;  95% confidence intervals were obtained for all parameters. | |
| outcomes (Mean values for the main categories of estimated outcomes of interest, as well as mean differences between the comparator groups.) | - The average rate of increase pre-order was 0.113 (95% C.I.: 0.110, 0.115) per day and post-order was 0.047 (95% C.I.: 0.045, 0.048) per day. | |
| Characterising heterogeneity | Data for each state are presented in the manuscript. | |
|  | | |
| Discussion | | |
| Study findings, (Summarise key study findings and describe how they support the conclusions reached.) | The data suggest a remarkably consistent and important effect associated with the issuing of stay-at-home orders and are generally supportive of such measures. | |
| limitations | - First, it would be impossible to isolate the effect of these orders against the background of numerous other local, state, and federal interventions occurring at the same time. - Second, the expected COVID-19 expansion curve in the absence of interventions to reduce the transmission is unknown. As such, it is possible that the observed slope changes are driven by the natural history of the pandemic as well as the specific policies in question. - Third, states implemented stay-at-home orders in response to the pandemic and these observations are thus profoundly threatened by selection and indication biases. - Fourth, even with the use of trend data in this analysis, there is a threat of regression to the mean if stay-at-home orders were consistently placed at the peak of epidemic growth. - Finally, and perhaps most importantly, a major limitation of this work is the endogenous relationship between case counts and both the availability and use of testing. | |
| generalisability | - | |
| current knowledge (how the findings fit with current knowledge.) | Not only was the availability of tests evolving over this time period, but there is a direct relationship between the number of infections and public awareness of the pandemic and the number of tests performed. As such, it would be difficult to begin to parse out the effect of testing on the rate of incidence of COVID-19. That said, it is likely that the availability of tests may have an unmeasured impact on these results, but we cannot know if the impact was to increase or decrease the incidence rates. | |
| changes in diagnostic tests and access and any adjustment | The availability of testing was a significant barrier to COVID-19 diagnoses in the early stages of the pandemic in the U.S. and improved gradually over the month of March. Tests per day increased roughly tenfold between March 15th and March 31st and have increased at a more gradual rate of fourfold over the last six weeks. | |

1. **Comparison of estimated rates of coronavirus disease 2019 (COVID-19) in border counties in Iowa without a stay-at-home order and border counties in Illinois with a stay-at-home order(Lyu & Wehby, 2020)**

| Data Collection Item | Item description / sub-groups | |
| --- | --- | --- |
| Bibiliographic Information | | |
| Author | Lyu et al. | |
| Title | Comparison of estimated rates of coronavirus disease 2019 (COVID-19) in border counties in Iowa without a stay-at-home order and border counties in Illinois with a stay-at-home order | |
| Year of publication | 2020 | |
| Country | USA | |
|  | | |
| Methods | | |
| Type of Study | cross-sectional study with a difference-in-differences design | |
| Time period and setting of the study | Border counties in Iowa and Illinois; from March 15 (when the first case was reported in those counties) to March 21. | |
| Intervention | stay-at-home order | |
| description of intervention | Illinois issued a stay-at-home order on March 21, 2020. | |
| setting of the intervention | Seven Illinois counties bordering Iowa (ie, Carroll, Hancock, Henderson, Jo Daviess, Mercer, Rock Island, and Whiteside) | |
| Aim/ Objectives | To compare COVID-19 cases in border counties in Iowa, which did not issue a stay-at-home order, with cases in border counties in Illinois, which did issue a stay-at-home order. | |
| Participant characteristics | Participants | 7 Illinois counties (n= 272 385), which did issue a stay-at-home order; (population density =78.2 people per square mile); (population poverty= 0.8%) |
|  | Comparator details | 8 Iowa counties (n= 462 445), which did not issue a stay-at-home order; (population density= 114.2 people per square mile); (population poverty= 12.1%) |
| Outcome measures | Daily cumulative cases | cumulative cases of COVID-19 per 10 000 residents |
| Source of data | - County-level COVID-19 data come from a repository of state and local health agency reports (GitHub. An ongoing repository of data on coronavirus cases and deaths in the U.S.). | |
| Data analyiss | - Difference-in-differences regression using daily county-level COVID-19 data was performed. - The authors also performed two sensitivity analyses. The first sensitivity analysis accounted for differences in the timing of closing schools and nonessential businesses between the 2 states. The second sensitivity analysis examined whether there were differential trends in COVID-19 cases by county population density and poverty rates, reported by census data, that may have confounded the difference-indifferences estimates. - Data analyses were conducted with Stata/SE version 16.0 (StataCorp). Statistical significance was set at P < .05, and all tests were 2-tailed | |
|  | | |
| Results | | |
| Study parameters | Daily cumulative cases of COVID-19 per 10 000 residents. | |
| outcomes (Mean values for the main categories of estimated outcomes of interest, as well as mean differences between the comparator groups.) | - Trends of cumulative COVID-19 cases per 10 000 residents for the Iowa and Illinois border counties were comparable before the Illinois stay-at-home order, which went into effect at 5:00 PM on March 21 (March 15 to March 21: 0.024 per 10 000 residents vs 0.026 per 10 000 residents). After that, cases increased more quickly in Iowa and more slowly in Illinois. - Within 10, 20, and 30 days after the enactment of the stay-at-home order in Illinois, the difference in cases was −0.51 per 10 000 residents (SE, 0.09; 95%CI, −0.69 to −0.32; P < .001), −1.15 per 10 000 residents (SE, 0.49; 95%CI, −2.12 to −0.18; P = .02), and −4.71 per 10 000 residents (SE, 1.99; 95%CI, −8.64 to −0.78; P = .02), respectively. - The estimates indicate excess cases in the border Iowa counties by as many as 217 cases after 1 month without a stay-at-home order. This estimate of excess cases represents 30.4%of the 716 total cases in those Iowa counties by that date. - In the first sensitivity analysis accounting for differences in the timing of closing schools and nonessential businesses between the 2 states, the difference-in-differences estimates for the stay-at-home order were similar, although not statistically significant at 20 and 30 days (eg, difference in COVID-19 cases per 10 000 residents in Illinois at 10 days, −0.51; SE, 0.10; 95%CI, −0.71 to −0.31; P < .001; 20 days, −1.18; SE, 0.61; 95%CI, −2.39 to 0.04; P = .06; 30 days, −4.73; SE, 2.57; 95%CI, −9.81 to 0.35; P = .07). - In the second sensitivity analysis examining whether there were differential trends in COVID-19 cases by county population density and poverty rates that may have confounded the difference-in-differences estimates, the difference-in-differences estimates were robust to adding these covariates (eg, difference in COVID-19 cases per 10 000 residents in Illinois at 10 days, −0.50; SE, 0.10; 95%CI, −0.70 to −0.30; P < .001; 20 days, −1.13; SE, 0.53; 95%CI, −2.18 to −0.77; P = .04; 30 days, −4.80; SE, 2.517; 95%CI, −9.09 to −0.52; P = .03). | |
| Characterising heterogeneity | - | |
|  | | |
| Discussion | | |
| Study findings, (Summarise key study findings and describe how they support the conclusions reached.) | This cross-sectional study of counties along the border of Iowa and Illinois provides early evidence suggesting that issuing a stay-at-home order in Iowa while daily cases continued to increase may have helped limit the spread of COVID-19 cases in Iowa. | |
| limitations | - Findings should be interpreted cautiously considering that possible differences in COVID-19 testing across the border may confound the difference-in-differences estimates. | |
| generalisability |  | |
| current knowledge (how the findings fit with current knowledge.) | - In the last 2 days of the study period (ie, April 19-20), Iowa began announcing an increase in cases from surveillance testing following outbreaks in meat-processing facilities, including 1 facility in Louisa County. That outbreak appears to have been first reported on April 6. As described earlier, there was a trend of more cases in Iowa before April 6 and the trend increased during most of the study period. Therefore, the surveillance testing reported toward the end of the study period does not appear to explain the whole trend. - A meat-packing facility in Iowa had an outbreak, which increased surveillance. Whether stay-at-home orders affect the likelihood of such outbreaks and their spillovers into the community is an open question for future research. | |
| changes in diagnostic tests and access and any adjustment | Illinois had a greater increase in tests per 10 000 residents following the stay-at-home order. Both states started with a similar testing rate, but the testing rate in Illinois increased after the stay-at-home order at a faster rate than in Iowa (10 days, 28 vs 23 tests per 10 000 residents; 20 days, 69 vs 51 tests per 10 000 residents; 30 days, 117 vs 82 tests per 10 000 residents). | |

1. **Association of stay-at-home orders with COVID-19 hospitalizations in 4 states.(Sen et al., 2020)**

| Data Collection Item | Item description / sub-groups | |
| --- | --- | --- |
| Bibiliographic Information | | |
| Author | Sen et al. | |
| Title | Association of stay-at-home orders with COVID-19 hospitalizations in 4 states | |
| Year of publication | 2020 | |
| Country | USA | |
|  | | |
| Methods | | |
| Type of Study | Retrospective observational | |
| Time period and setting of the study | - States included in this sample were Colorado, Minnesota, Ohio, and Virginia; March 10 Through April 28, 2020. - Among the 4 states meeting the inclusion criteria, the earliest date with data on hospitalizations was March 10. Because the median incubation period of COVID-19 was reported to be 4 to 5.1 days3,4 and the median time from first symptom to hospitalization was found to be 7 days,5 we hypothesized that any association between stay-at-home orders and hospitalization rates would become evident after 12 days (median effective date). | |
| Intervention | Stay-at-home order. | |
| description of intervention | As of April 18, 2020, governors in 42 states had issued statewide executive “stay-at-home” orders to help mitigate the risk that COVID-19 hospitalizations would overwhelm their state’s health care infrastructure. | |
| setting of the intervention | 42 states had issued statewide executive “stay-at-home” orders | |
| Aim/ Objectives | To assess the association between these orders and hospitalization trends. | |
| Participant characteristics | Participants | Colorado, Minnesota, Ohio, and Virginia |
|  | Comparator details |  |
| Outcome measures | cumulative hospitalizations | Cumulative Hospitalizations Due to COVID-19 |
| Source of data | Data were obtained from each state’s department of health website on a daily basis. | |
| Data analyiss | - Exponential growth function was fit to cumulative hospitalization data. - 95% prediction bands were computed on the exponential fit line to determine if the observed number of hospitalizations fell within the interval. - All analyses were performed using Microsoft Excel version 14.1. | |
|  | | |
| Results | | |
| Study parameters | Cumulative Hospitalizations growth rates per day. | |
| outcomes (Mean values for the main categories of estimated outcomes of interest, as well as mean differences between the comparator groups.) | - In all 4 states, cumulative hospitalizations up to and including the median effective date of a stay-at-home order closely fit and favored an exponential function over a linear fit (R2 = 0.973 vs 0.695 in Colorado; 0.965 vs 0.865 in Minnesota; 0.98 vs 0.803 in Ohio; 0.994 vs 0.775 in Virginia). - However, after the median effective date, observed hospitalization growth rates deviated from projected exponential growth rates with slower growth in all 4 states. Observed hospitalizations consistently fell outside of the 95% prediction bands of the projected exponential growth curve. | |
| Characterising heterogeneity | - | |
|  | | |
| Discussion | | |
| Study findings, (Summarise key study findings and describe how they support the conclusions reached.) | - In 4 states with stay-at-home orders, cumulative hospitalizations for COVID-19 deviated from projected best-fit exponential growth rates after these orders became effective. - The deviation started 2 to 4 days sooner than the median effective date of each state’s order and may reflect the use of a median incubation period for symptom onset and time to hospitalization to establish this date. | |
| limitations | Limitations of the study include that these other factors could not be modelled in the analysis and that data on only 4 states were available. | |
| generalisability |  | |
| current knowledge (how the findings fit with current knowledge.) | - Other factors that potentially decreased the rate of virus spread and subsequent hospitalizations include school closures, social distancing guidelines, and general pandemic awareness. - In addition, economic insecurity and loss of health insurance during the pandemicmay have also decreased hospital utilization. | |
| changes in diagnostic tests and access and any adjustment | - | |

1. **Comprehensive public health evaluation of lockdown as a non-pharmaceutical intervention on COVID-19 spread in India: National trends masking state-level variations(Salvatore et al., 2020)**

| Data Collection Item | Item description / sub-groups | |
| --- | --- | --- |
| Bibiliographic Information | | |
| Author | Salvatore et al. | |
| Title | Comprehensive public health evaluation of lockdown as a non-pharmaceutical intervention on COVID-19 spread in India: National trends masking state-level variations | |
| Year of publication | 2020 | |
| Country | India | |
|  | | |
| Methods | | |
| Type of Study | Cohort study | |
| Time period and setting of the study | - From March 25 to May 31; India - All our analyses use data available from 15 March until 31 March, 31 March being the day India’s strict national lockdowns ended and ‘unlocking’ started. | |
| Intervention | Lockdown | |
| description of intervention | State level variations were adopted along with national lockdown.  Nationwide measures implemented as mentioned below:  **Phase 1 (25 March to 14 April):** All transport services—road, air and rail—were suspended, with exceptions for transportation of essential goods, fire, police and emergency services. Educational institutions, industrial establishments and hospitality services were also suspended. Services such as food shops, banks and ATMs, petrol pumps, other essentials and their manufacturing were exempted.  **Phase 2 (15 April to 3 May)**: Conditional relaxation promised after 20 April, subject to containment of spread. Lockdown areas classified into red, orange and green zones based on extent of spread of disease. Certain relaxations from 20 April: agricultural businesses, including dairy, aquaculture and plantations allowed to open. Cargo transportation vehicles allowed to operate. Banks and government centres distributing benefits allowed to open as well.  **Phase 3 (4–17 May):** Zonal classification of regions into red, orange and green zones continued, with normal movement allowed in green zones. Movement of private and hired vehicles allowed in orange zones and red zones remained in lockdown. Zonal classifications revised on a weekly basis  **Phase 4 (18–31 May):** Unlike the previous phases, states were given a larger say in the demarcation of green, orange and red zones and the implementation roadmap. Red zones were further divided into containment and buffer zones. Local administrative bodies were given the authority to demarcate containment and buffer zones. | |
| setting of the intervention | All states of India | |
| Aim/ Objectives | To evaluate the effect of four-phase national lockdown from March 25 to May 31 in response to the COVID-19 pandemic in India and unmask the state-wise variations in terms of multiple public health metrics. | |
| Participant characteristics | Participants | Confirmed COVID-19 cases nationally and across 20 states that accounted for >99% of the current cumulative case counts in India until 31 May 2020. |
|  | Comparator details |  |
| Outcome measures | time-varying  effective basic reproduction number, R | The average number of persons infected by an infected individual |
| Source of data | Publicly available data were used for all analyses (covid-19india. org and Our World In Data). | |
| Data analyiss | - The estimation of the time-varying R was performed using the EpiEstim package in R and daily case count data from COVID-19 India. - In particular, the authors used the vectors of daily new cases as the input using the ‘parametric_SI’ estimation method and a 5-day window (‘estimate_R’ function, which was used to describe the progression of the outbreak in Wuhan). - We also use a gamma distribution prior with a mean of 7 days and an SD of 4.5 days, based on research by Wu and colleagues, for the generation time (a distribution of the onset of disease used to estimate R). | |
|  | | |
| Results | | |
| Study parameters | Daily number of reported cases, fatalities and recovered cases;  7-day average estimate for reproduction number. | |
| outcomes (Mean values for the main categories of estimated outcomes of interest, as well as mean differences between the comparator groups.) | - The estimated effective reproduction number R for India was 3.36 (95% CI 3.03 to 3.71) on 24 March, whereas the average of estimates from 25 May to 31 May stands at 1.27 (95% CI 1.26 to 1.28). - Similarly, the estimated doubling time across India was at 3.56 days on 24 March, and the past 7-day average for the same on 31 May is 14.37 days. - The average daily number of tests increased from 1717 (19–25 March) to 113 372 (25–31 May) while the test positivity rate increased from 2.1% to 4.2%, respectively. However, various states exhibit substantial departures from these national patterns. | |
| Characterising heterogeneity |  | |
|  | | |
| Discussion | | |
| Study findings, (Summarise key study findings and describe how they support the conclusions reached.) | Patterns of change over lockdown periods indicate the lockdown has been partly effective in slowing the spread of the virus nationally. However, there exist large state-level variations and identifying these variations can help in both understanding the dynamics of the pandemic and formulating effective public health interventions. | |
| limitations | - First, the metrics presented do not include predictions of future daily active cases and thus do not inform us about projected healthcare needs. - Second, the methods do not account for age-sex structure and mobility patterns in India. - Third, the quality of the data is in question with the existence of evidence that reported case counts are significantly lower than true case counts. - Finally, we consider a narrow evaluation of the lockdown in terms of COVID-19-related outcomes using data up to 31 May. There are many long-term and broader consequences of the lockdown that this paper fails to capture. | |
| generalisability | - | |
| current knowledge (how the findings fit with current knowledge.) | - Differences in estimates of R and estimated DTs suggest that peaks will vary across states. - Steady decline in test positivity rates (TPRs) to 5% or less for at least 14 consecutive days may indicate the pandemic is in a control phase, that is, an indication that effective R is declining and less than 1. - The testing shortfall metric can then be gainfully employed to determine the number of tests that need to be done randomly in the community for surveillance during a control phase. In order to devise a testing strategy, it is important not just to think about the number of tests but consider various types of tests, including rapid antigen test, RT-PCR test and cost-efficient testing strategies such as pooled testing, stratified periodic sampling to capture asymptomatic individuals. The goal of testing be it for clinical diagnostic purpose, screening or surveillance should be clear. - The testing shortfall metric may indicate that we need to carry out a large number of tests that we do not have resources for, but this number can inform us when and where to scale up syndromic surveillance using community and government healthcare workers. - Given the spatial and temporal pattern of the pandemic’s spread, it is extremely important to prioritise policies. Resources must be mobilised to help one cluster of states and then move to the next cluster. | |
| changes in diagnostic tests and access and any adjustment | - | |

1. **The effect of lockdown regulations on SARS-CoV-2 infectivity in Gauteng Province, South Africa(Pillai et al., 2020)**

| Data Collection Item | Item description / sub-groups | |
| --- | --- | --- |
| Bibiliographic Information | | |
| Author | Pillai et al. | |
| Title | The effect of lockdown regulations on SARS-CoV-2 infectivity in Gauteng Province, South Africa | |
| Year of publication | 2020 | |
| Country | South Africa | |
|  | | |
| Methods | | |
| Type of Study | Retrospective cohort study | |
| Time period and setting of the study | Gauteng Province of South Africa during a 4-month period (5 March - 30 June 2020). | |
| Intervention | Lockdown | |
| description of intervention | - On 26 March 2020, the South African (SA) government initiated a 21-day national level 5 lockdown which was subsequently eased off and downgraded to level 4 on 1 May and to level 3 on 1 June. - ALERT LEVEL 5 ((5 - 31 March)): Drastic measures to contain the spread of the virus and save lives. - ALERT LEVEL 4 (1 - 31 May): Extreme precautions to limit community transmission and outbreaks, while allowing some activity to resume - ALERT LEVEL 3 (1 - 30 June): Restrictions on many activities, including at workplaces and socially, to address a high risk of transmission. - ALERT LEVEL 2: Physical distancing and restrictions on leisure and social activities to prevent a resurgence of the virus. - ALERT LEVEL 2: Most normal activity can resume, with precautions and health guidelines followed at all times Population prepared for an increase in alert levels if necessary. | |
| setting of the intervention | South Africa | |
| Aim/ Objectives | To measure the effects of lockdown measures introduced in SA on SARS-CoV-2 attack rates (ARs, the percentage of individuals who tested positive in a specified time period) in Gauteng Province during a 4-month period (March - June 2020). | |
| Participant characteristics | Participants | A total of 162 528 tests were performed at the laboratory between 5 March and 30 June 2020. Of these, 20 574 were positive (overall AR 12.7%). These positive tests constituted 44.8% of all positive cases reported in the province (20 574/45 944). |
|  | Comparator details |  |
| Outcome measures | Attack rates (AR) | the percentage of individuals who tested positive over a specified time period |
| Source of data | A comprehensive database from an independent pathology laboratory in Johannesburg, Gauteng, was used during a 4-month period (5 March 2020 - 30 June 2020). | |
| Data analyiss | A logistic regression model with outcome testing was used for assessment of the effects of lockdown on the AR. | |
|  | | |
| Results | | |
| Study parameters | AR was reported as the percentage of individuals who tested positive over a specified time period;  percentage of individuals who tested positive | |
| outcomes (Mean values for the main categories of estimated outcomes of interest, as well as mean differences between the comparator groups.) | - The numbers of tests performed during March, April, May and June were 12 430 (7.6%), 11 384 (7.0%), 37 409 (23.1%), and 101 305 (62.3%), respectively. There was a decrease during level 5 lockdown (April), followed by increases during level 4 (May) and level 3 (June). - Individuals in the age groups 31 - 40 and 41 - 50 years had the highest number of tests done (27.5% and 19.6%, respectively). Individuals aged <10 years and >60 years had 4.5% and 11.6% of all tests, respectively. More females (54.8%) than males were tested. The AR was significantly higher in males (13.2%) compared with females (12.1%) (χ2 test, p<0.0001). - All regions showed a remarkable increase in ARs in June. - There were differential increases in AR for all age groups over time. For all individuals, the AR during pre-lockdown, level 5 lockdown, level 4 lockdown and level 3 lockdown was 4.1%, 2.2%, 3.4% and 18.5%, respectively. There was a rapid increase in AR during level 3, when additional restrictions were lifted. - For the age group 0 - 20 years, the AR during pre-lockdown and levels 5, 4 and 3 lockdown was 3.5%, 1.0%, 2.7% and 15.9%, respectively. A rapid increase in AR was noted in level 3. Relative to the pre-lockdown period, the odds of testing positive for this age group were 70% lower in level 4 (adjusted odds ratio (aOR) 0.3; 95% confidence interval (CI) 0.15 - 0.61;p<0.01) and 21% lower in level 3 (aOR 0.79; 95% CI 0.53 - 1.18). In level 5, the odds of infection were 427% higher compared with the pre-lockdown level. - For the age group 21 - 60 years, the AR during pre-lockdown and levels 5, 4 and 3 lockdown was 4.0%, 2.4%, 3.8% and 19.3%, respectively. A rapid increase in AR was also noted in level 3. Relative to the prelockdown period, the aOR was 42% lower in level 5, 2% lower in level 4 and 469% higher in level 3. In this age group, males had a 1.1 times (95% CI 1.06 - 1.13) higher odds of testing positive compared with females. - For the age group >60 years, the AR during pre-lockdown and levels 5, 4 and 3 lockdown was 5.4%, 2.0%, 2.2% and 15.3%, respectively. Again, a rapid increase in AR was noted for this age group in level 3. Relative to the pre-lockdown period, the aOR for infection was 65% and 62% lower in level 5 and level 4, respectively, whereas it increased by 215% in level 3. - ARs decreased from pre-lockdown rates to a lower rate in level 5 (most restrictive period) and level 4 (when a number of essential services were allowed). The AR then increased sharply in level 3 (when many industries and schools reopened). The trends were similar when 14-day moving average ARs were weighted for age and gender. | |
| Characterising heterogeneity | - | |
|  | | |
| Discussion | | |
| Study findings, (Summarise key study findings and describe how they support the conclusions reached.) | - This analysis showed that the AR of SARS-CoV-2 infection increased rapidly from 3.3% (March - May) to 18.3% (June) after lockdown was eased to level 3. Although the number of tests increased in June, the magnitude of the spread is reflected in the increase in the ARs across all age groups - The infectivity respite during the lockdown created an opportunity for healthcare planning and preparation. The ‘hard’ level 5 lockdown may merely have delayed the surge of infection observed during subsequent levels. | |
| limitations | - This study is based on retrospective analysis of secondary data from a private pathology laboratory. - Anonymised data did not allow the authors to quantify these individuals. In addition, it is not clear to what extent the change to a more selective testing policy influenced requests made to private laboratories (and the high AR). | |
| generalisability | This study is based on retrospective analysis of secondary data from a private pathology laboratory. The findings may therefore not be generalisable. However, this laboratory covers all the districts in the province, and 40% of the positive cases in the province during the 4-month study period was analysed. Also, a number of RT-PCR tests were requests made by National Health Laboratory Service. | |
| current knowledge (how the findings fit with current knowledge.) | - While the imposed lockdown slowed down viral transmission, the economic and financial trade-off was non-sustainable. Preservation of the economy is largely dependent on the 20 - 60-year age group. The age groups <20 and >60 years jointly comprise 40% of the Gauteng population. Our data indicate that both these groups contribute significantly to the AR and infectivity. Balancing lockdown regulations with ongoing economic activity using age and geography as discriminators needs careful consideration. - The relatively low number of tests done in the age groups 0 - 20 and >60 years is a concern in view of the findings of this study and would require further attention. | |
| changes in diagnostic tests and access and any adjustment | - | |

1. **Effective control of SARS-CoV-2 transmission in Wanzhou, China(Shi & Hu, 2021)**

| Data Collection Item | Item description / sub-groups | |
| --- | --- | --- |
| Bibiliographic Information | | |
| Author | Shi et al. | |
| Title | Effective control of SARS-CoV-2 transmission in Wanzhou, China | |
| Year of publication | 2021 | |
| Country | China | |
|  | | |
| Methods | | |
| Type of Study | Observational epidemiological study | |
| Time period and setting of the study | Wanzhou district at Chongqing province; Between 21 January and 10 April 2020 | |
| Intervention | Face mask wearing, thorough contact tracing, mass testing, identification and early diagnosis of presymptomatic and asymptomatic cases and strict quarantine of close contacts. | |
| description of intervention | - Following the lockdown of Wuhan city, the municipality of Chongqing initiated the first-level response to major public health emergencies on 24 January 2020. - On 6 February 2020, a protocol, which necessitated close contacts of infected persons to either home quarantine or further testing and examinations, was amended to require that all close contacts be quarantined in centralized locations (in a hotel if they did not report symptoms and in a hospital if they reported symptoms) rather than at home and tested by RT–PCR twice during the centralized quarantine. Wanzhou CDC adopted this amended protocol on 8 February 2020. - All close contacts who were identified before 8 February 2020 and were home quarantined took the RT–PCR test on 8 February 2020 or immediately after. All COVID-19 cases confirmed by RT–PCR, regardless of symptom status, were treated in the designated hospitals. | |
| setting of the intervention | Chongqing province | |
| Aim/ Objectives | To present the full course of the outbreak in this city and to assess the effectiveness of control measures through an analysis of SARS-CoV-2 transmission in each generation by asymptomatic, presymptomatic and symptomatic cases and an examination of the contact patterns that may have facilitated transmission in the study population during the outbreak. | |
| Participant characteristics | Participants | - Wanzhou district with 1.74 million population size is a city in the municipality of Chongqing bordering the western side of Hubei Province, of which Wuhan is the capital city. - Between 21 January and 10 April 2020, 183 confirmed cases and 1,983 close contacts who tested negative for SARS-CoV-2 were identified in Wanzhou - The samples were 2166 Covid-19 cases and their close contacts with a mean age of 42.7 years (1170 men and 9988 women) |
|  | Comparator details |  |
| Outcome measures | Reproductive number | The number of people on average that an infected person can transmit the virus to—by using transmission over each generation, and its 95% CI was derived by bootstrapping with 5,000 resamples. |
| Source of data | Data were thus extracted from (1) epidemiological investigation reports of all positive cases tested by RT–PCR from 21 January through 10 April 2020 and (2) contact-tracing records of close contacts identified by Wanzhou CDC. | |
| Data analyiss | - Modelling approach proposed by Lloyd-Smith and colleagues to calculate the Reproductive number. - Risk factors for SARS-CoV-2 infection were analyzed using multilevel logistic regression (two-tailed likelihood ratio test, P < 0.01). - Data analyses were performed with SAS v9.4 (SAS Institute), R v4.0.2 (R Core Team) and Visual C++ v6.0 | |
|  | | |
| Results | | |
| Study parameters | SARS-CoV-2 transmission over five generations (G1 to G5);  Percentage of cases. | |
| outcomes (Mean values for the main categories of estimated outcomes of interest, as well as mean differences between the comparator groups.) | - The estimated G1-to-G2 reproductive number was 1.64 (95% CI: 1.16–2.40). After strict control measures were implemented, the reproductive number decreased dramatically to 0.39 (95% CI: 0.24–0.58) for G2-to-G3 transmission and to 0.31 (95% CI: 0.12–0.58) for G3-to-G4 transmission. - Stratifying by case type, the G1-to-G2 reproductive numbers were 1.63 (95% CI: 1.03–2.59) and 2.44 (95% CI: 2.12–6.75) for symptomatic and asymptomatic source cases, respectively. The offspring distribution of symptomatic and asymptomatic   cases both had large individual variation. | |
| Characterising heterogeneity | - | |
|  | | |
| Discussion | | |
| Study findings, (Summarise key study findings and describe how they support the conclusions reached.) | - Dining together was the major mode of contact and accounted for 56% of the contacts before 25 January 2020, and it was reduced to 39% between 25 January and 13 February 2020 due to social distancing. - No contact via dining together occurred after 13 February 2020. - The substantial decrease in the number of contacts over time as a result of the above-described control measures may have contributed to the rapid reduction in reproductive numbers in G2–G4. | |
| limitations | - First, since contact tracing was conducted via interview, recall bias could lead to inadequate contact tracing, particularly for asymptomatic cases who had a longer transmission risk period. - Secondly, the majority of asymptomatic cases were identified after 8 February 2020 when all close contacts were required to be tested by RT–PCR. The contact tracing for asymptomatic cases who were labeled as ‘home-quarantined close contacts with no symptoms’ before 8 February 2020 could be insufficient as these asymptomatic cases may not be able to recall all the close contacts due to the long time window between having contact with their source cases and being diagnosed, leading to a smaller ratio of cases to close contacts for asymptomatic cases than for symptomatic cases. Moreover, close contacts were identified according to the definitions used by the National Health Commission of China, by which a relatively large symptomatic ratio of cases to close contacts (1:18) was identified in the population. | |
| generalisability | - Around 47 other Chinese cities in 22 provinces have a similar population size to Wanzhou (1.5 to 2.5 million) and implemented the same containment measures to tackle the COVID-19 outbreak. - In addition, Wanzhou is comparable in population size to medium- to large-sized cities in Europe and the United States. - These characteristics make Wanzhou a suitable example for better understanding the transmission dynamics and risk factors of SARS-CoV-2 infection, as well as the effectiveness of control measures, for both researchers and policy makers in countries and cities that are heavily hit by the COVID-19 outbreak. - However, the lack of consistent criteria may limit the interpretation and generalization of our results into other contexts. | |
| current knowledge (how the findings fit with current knowledge.) | - The spread of COVID-19 was effectively controlled in Wanzhou by social distancing, including face mask wearing, thorough contact tracing, mass testing, identification and early diagnosis of presymptomatic and asymptomatic cases and strict quarantine of close contacts. Targeting the main risk factors—the timing, frequency and duration of contact—will be key interventions for mitigating COVID-19 and for better handling of possible resurgence in the future. | |
| changes in diagnostic tests and access and any adjustment | - | |

1. **The effects of containment measures in the Italian outbreak of COVID-19(Supino et al., 2020)**

| Data Collection Item | Item description / sub-groups | |
| --- | --- | --- |
| Bibiliographic Information | | |
| Author | Supino et al. | |
| Title | The effects of containment measures in the Italian outbreak of COVID-19 | |
| Year of publication | 2020 | |
| Country | Italy | |
|  | | |
| Methods | | |
| Type of Study | Observational; cross-sectional | |
| Time period and setting of the study | February 24^th^ to April 5^th^ ; Italia   - We expect the effect on the number of ICU patients to appear within about 2 weeks (i.e., the maximum incubation time), and the effect on the number of deaths to appear in about 3 weeks (i.e., the time from infection to death). - Because of the saturation of the ICU beds in several Italian regions, the number of ICU beds occupied in Italy was lower than the patients that required intensive care. Therefore, we analyze the Italian data excluding the regions where the ICUs had saturated. This leaves us with 15 of the 20 regions, excluding about 28% of the Italian population. | |
| Intervention | lockdown measures | |
| description of intervention | Italy imposed two major containment measures: the lockdown on March 9th 2020, and the full lockdown on March 21st 2020, where all non-essential companies and manufacturing plant were closed. | |
| setting of the intervention |  | |
| Aim/ Objectives | To assess short-term and long-term lockdown effects on intensive care units and number of deaths | |
| Participant characteristics | Participants | 15 of the 20 regions in Italy, excluding about 28% of the Italian population |
|  | Comparator details |  |
| Outcome measures | ICU patients |  |
|  | COVID-19 deaths |  |
| Source of data |  | |
| Data analyiss | - The data points were divided into three groups, before March 14th, between March 14th and March 24th, and up to April 5th. - The non-linear least squares problems have been solved using Levenberg-Marquardt algorithm, with NumPy library (Python) | |
|  | | |
| Results | | |
| Study parameters | ICU patients growth rate;  COVID-19 deaths growth rate. | |
| outcomes (Mean values for the main categories of estimated outcomes of interest, as well as mean differences between the comparator groups.) | - The number of ICU patients grows slower than exponential starting from March 14th, i.e., five days after the lockdown of March 9th. - After 5 days after the lockdown the growth of ICU patients start changing from exponential to linear, and in about 28 days reaches the upper plateau of the logistic trend. - Regarding the number of deaths, after about 11 days the growth changes from exponential to linear, and after about 120 days the number approaches a plateau. | |
| Characterising heterogeneity | - | |
|  | | |
| Discussion | | |
| Study findings, (Summarise key study findings and describe how they support the conclusions reached.) | The Italian case demonstrates that the national lockdown is effective in reducing the growth of ICU patients. | |
| limitations | - | |
| generalisability | - COVID-19 epidemic in Italy can be used as a precedent for other countries; also, the measures used in this study, ICU hospitalization is much more dependent to the infection than to the characteristics of countries such as testing capacity and strategy. | |
| current knowledge (how the findings fit with current knowledge.) | - The saturation of the ICUs in many Italian regions suggests that containment measures were taken too late. Using Italy as precedent, other countries should impose these confinement measures at earlier stages of the outbreak to be able to protect their population from COVID-19. - We show that countries can predict the date of saturation of their ICUs early on, as soon as an exponential growth of intensive care patients is observed, as it was in Lombardy region. | |
| changes in diagnostic tests and access and any adjustment | - | |

1. **Impact of mass screening on the number of confirmed cases, recovered cases, and deaths due to COVID-19 in Iran: An interrupted time series analysis(Soodejani et al., 2020)**

| Data Collection Item | Item description / sub-groups | |
| --- | --- | --- |
| Bibiliographic Information | | |
| Author | Soodejani et al. | |
| Title | Impact of mass screening on the number of confirmed cases, recovered cases, and deaths due to COVID-19 in Iran: An interrupted time series analysis | |
| Year of publication | 2020 | |
| Country | Iran | |
|  | | |
| Methods | | |
| Type of Study | Interrupted time series analysis | |
| Time period and setting of the study | Cases reported daily to the Ministry of Health and Medical Education (MOHME) from February 19 through May 6, 2020. | |
| Intervention | Mass screening | |
| description of intervention | - At the onset of the epidemic in Iran February 19, 2020, testing and surveillance for COVID-19 were passive, with cases reported from health centers where individuals were treated. - After March 23, 2020, mass screening by COVID-19 PCR testing and CT scan of the chest wasscaled up throughout the country. Case reporting thereafter occurred through several avenues, including completion of an online self-declaration form, health authorities making phone calls to people in the community, and contact tracing. - People with symptoms of COVID-19 were referred to the nearest designated center for further examination and diagnostic testing. In combination, these methods led to the screening of more than 70 million people, which is over 90% of the Iranian population. | |
| setting of the intervention | Community of Iran | |
| Aim/ Objectives | To evaluate the impact of the change towards mass screening on new cases reported, cases recovered, and deaths due to COVID-19 | |
| Participant characteristics | Participants | - A total of 519 544 diagnostic COVID-19 PCR tests were performed in Iran. Overall, 101 650 cases were confirmed (case/test ratio 19.6%). - During this period, 81 578 confirmed cases recovered and 6418 died (case fatality ratio 6.3%); 13 654 (13.4%) were hospitalized with unknown outcome. |
|  | Comparator details |  |
| Outcome measures | Confirmed cases | Number of reported COVID-19 cases confirmed by positive polymerase chain reaction (PCR) test, |
|  | Recovered cases | The number recovered from infection by 14 days after resolution of symptoms and a negative PCR test result |
|  | COVID-19 deaths | Number of reported deaths due to COVID-19. |
| Source of data | Data available to the public on the MOHME website8 and from the World Health Organization (WHO) | |
| Data analyiss | - Interrupted time series analysis - Diagnostic plots were used to check linear assumptions. - Durbin-Watson statistic was used to detect autocorrelation. | |
|  | | |
| Results | | |
| Study parameters | Daily reports on the number of new cases;  Confirmed cases rate change;  Recovered cases rate change;  COVID-19 deaths rate change;  Weekly percent change was calculated. | |
| outcomes (Mean values for the main categories of estimated outcomes of interest, as well as mean differences between the comparator groups.) | - The pre-mass screening period shows a significant increase in the rate of reported deaths over time (β1 109.7, 95% CI 90.3, 129.1). No significant change was seen in the number of reported deaths immediately pre- versus post-mass screening (β2 6.9, 95% CI, -12.7, 26.5). The number of deaths reported due to COVID-19 after mass screening decelerated, but not significantly (β3 -22.1, 95% CI -46.6, 2.4). - Changes in the number and percentage of confirmed cases, recovered cases, and deaths were investigated at the end of six weeks after the mass screening. As a differential from the expected (baseline or counterfactual), the observed results were an increase by 27.0% in confirmed cases, 85.4% in cases recovered, and a decrease of 13.4% in deaths. - The case/fatality ratio of COVID-19 before and after mass screening was 7.8% (95% CI 7.5, 8.2%) and 5.8% (95% CI 5.7, 6.0%), respectively. | |
| Characterising heterogeneity | - | |
|  | | |
| Discussion | | |
| Study findings, (Summarise key study findings and describe how they support the conclusions reached.) | Mass testing, with active case detection through contact tracing and isolation of cases, can ultimately help the country reduce morbidity and mortality due to COVID-19. | |
| limitations | - Data and analysis cannot prove that the mass screening measures caused reductions in the COVID-19. - Closure of schools, universities, religious centers, and commercial areas, as well as implementation of social distancing are likely to have impacted the course of the COVID-19 epidemic in Iran. | |
| generalisability | - | |
| current knowledge (how the findings fit with current knowledge.) | - With scale-up of mass screening for COVID-19 in Iran, the number of cases detected and reported to the MOHME accelerated substantially compared to the baseline period of passive detection and reporting. Detecting the highest number of persons with infection as early as possible can guide and improve control efforts such as isolation, contact tracing, and targeting of further testing. - Acceleration was seen in the cases recovered from COVID-19 following the scale-up of mass screening compared to baseline. Two explanations for this finding are possible. First, without mass screening, many COVID-19 cases, even those who were symptomatic, would have been undiagnosed and unreported. Therefore, their recovery from illness would also have been unreported. Second, mass screening would also diagnose mild and asymptomatic cases who would not have come to medical attention. | |
| changes in diagnostic tests and access and any adjustment | - | |

1. **Impact of mitigating interventions and temperature on the instantaneous reproduction number in the COVID-19 pandemic among 30 US metropolitan areas(Yu, 2020)**

| Data Collection Item | Item description / sub-groups | |
| --- | --- | --- |
| Bibiliographic Information | | |
| Author | Yu et al. | |
| Title | Impact of mitigating interventions and temperature on the instantaneous reproduction number in the COVID-19 pandemic among 30 US metropolitan areas | |
| Year of publication | 2020 | |
| Country | USA | |
|  | | |
| Methods | | |
| Type of Study | Time-serries data analysis | |
| Time period and setting of the study | 30 largest metropolitan areas in the USA; July 15, 2020   - Two time scales were considered in the analysis. The first was calendar date to present the trends of reproduction numbers for all metropolitan areas, starting from the date with at least 10 total reported cases. Staggered entrances into the outbreak were preserved. The second scale was the time since the beginning of the outbreak, regardless what calendar date the outbreak happened. This was to compare the declining patterns of Rt across metropolitan areas. We also realigned the time scale from the peak of the outbreak. The first two weeks of Rt estimates were excluded, as the first week Rt were zeros, and the second week estimates were too variable due to small number of cases. | |
| Intervention | stay at home rule | |
| description of intervention | CA Los Angeles, St. Francisco-St. Jose, Sacramento- Oakland; 19-Mar-20  CO Denver; 11-Apr-20  DC Washington DC - Baltimore; 1-Apr-20  FL Miami-Fort Lauderdale, Orlando; 3-Apr-20  GA Atlanta; 2-Apr-20  IL Chicago; 21-Mar-20  IN Indianapolis; 24-Mar-20  KS Kansas; 30-Mar-20  MA Boston; 24-Mar-20  MI Detroit; 24-Mar-20  MN Minneapolis-St. Paul; 27-Mar-20  MO St. Louis; 3-Apr-20  NC Durham-Raleigh, Charlotte; 30-Mar-20  NV Las Vegas; 1-Apr-20  NY New York 29.8% 40.4% 22-Mar-20  OH Cleveland, Columbus, Cincinnati; 22-Mar-20  OR Portland; 23-Mar-20  PA Philadelphia, Pittsburgh; 1-Apr-20  TX Houston, Dallas-Fort Worth; 2-Apr-20  UT Salt Lake City; 27-Mar-20  WA Seattle; 23-Mar-20  WI Milwaukee; 25-Mar-20 | |
| setting of the intervention | USA states | |
| Aim/ Objectives | To evaluate how mitigating interventions affected the epidemic process in the 30 largest metropolitan areas in the US and whether temperature played a role in the epidemic process. | |
| Participant characteristics | Participants | All metropolitan areas had at least 1.5 million people in 2019 and over 1000 confirmed cases. |
|  | Comparator details |  |
| Outcome measures | Time varying  (instantaneous) effective reproduction numbers (Rt) | The overall Rt at time t of the epidemic is the average number of secondary cases for all prior infected cases who are still infectious at a time window (t-s, t). |
|  |  |  |
| Source of data | - Data on daily COVID-19 cases and deaths at the US county level were obtained from the data repository provided by New York Times (https:// github.com/nytimes/covid-19-data, accessed on July 15,2020). - The sizes of total population and people aged 65 or above for each metropolitan area were obtained from census bureau website. - Information about stay-at-home rule for each state was scraped from popular news media. - The historical daily average temperature was obtained from national climate data online (<https://www7.ncdc.noaa>). gov/CDO), mostly based on temperature collected from stations at each metropolitan's main airport. | |
| Data analyiss | - Descriptive statistics and bivariate associations were reported. The sizes of total population and people aged 65 or older, and the percent of positive tests at each date were used for adjustment. - R package EpiEstim was used to estimate Rt. The Rt based on retrospective moving average were used to explore the trends. - Student t-tests were used to compare temperature and peak Rt cross-sectionally. - P value less than 0.01 was considered statistically significant to take account of many statistical comparisons involved. | |
|  | | |
| Results | | |
| Study parameters | The ratios of deaths to the size of elderly population | |
| outcomes (Mean values for the main categories of estimated outcomes of interest, as well as mean differences between the comparator groups.) | - The instantaneous Rts in all areas reached peaks or some stable points after two to three weeks, decreased significantly since the end of March, and most areas reached a Rt of around 1 after April 15. However, some small and short rebounds were presented in some areas. This might be due to case reporting and detection issues, but could also indicate some true rebounds. Throughout June and July, the Rts remained around 1 for most areas and reached 1.5 or higher in some areas, suggesting a persistent epidemic in those areas. It is of note that around the week of March 25, many schools were closed and many companies started offering employees working from home. The US government has issued COVID-19 coping guideline to all US citizens, and many states also issued stay-at-home. On the other hand, after June, the intervention mandates were relaxed in some areas, leading to large rebounds of Rt. - Boston, Chicago, New York and Philadelphia started the epidemic earlier, had higher peak Rts than that of Miami, Orlando, Houston, and Los Angeles. After some initial increases (though peaked at different dates), all northern cities declined sharply after the mid March. In addition, the trajectories of Houston and Los Angeles were similar with initial peaks at around March 18, somewhat decreased and then were stable around March 25. For Miami and Orland, the Rts were quite stable during the week of March 25, and declined sharply after about March 28, but had several rebounds in June and July. However, the slopes of decline, when aligned by the time since the peak Rt, were similar except a few spikes in Miami, Houston and Los Angeles. - On the other hand, the Rt curves were indistinguishable between upper midwestern cities and other southern cities. Upper Midwestern cities except Pittsburg all had earlier interventions in the mid-March, while Minneapolis-St. Paul area had some rebounds over the course of epidemic. In addition, the west coastal cities had an early start of the epidemic, and Rt curves were less volatile than that of other cities during the study period. The unusually high Rt in Salt Lake City in the early epidemic may be due to a small number of cases during that period. - After realigning the starting time from their respective outbreak peaks for all metropolitan areas, the overall declining patterns were similar across all regions. - Most cities had a peak Rt between 1 and 5. At the low temperature, peak Rts varied significantly. Cities with cold average temperature generally had higher Rts than those with warm temperature. However, upper midwestern cities such as Minneapolis-St. Paul, Milwaukee, and Columbus had much lower peak Rts than the rest of cities. The average peak of Rts in Boston, Chicago, New York, and Philadelphia were marginally (but not statistically significant) higher than that of Houston, Los Angeles, Orlando and Miami (average peak Rt 4.01 vs. 3.15, p = 0.07). In addition, the authors also arbitrarily examined the Rt patterns on the 15th day after the outbreak and on March 24, 2020 when most interventions had not fully executed. These cross-sectional analyses demonstrated similar patterns to that of peak Rt. | |
| Characterising heterogeneity | - As of July 15, 2020, the reported case fatality rates varied from 0.76 per 100 cases in Salt Lake City, UT to 10.77 per 100 cases in Detroit, MI. - Since about 80% of deaths occurred among elderly people, we compared the ratios of deaths to the size of elderly population among metropolitan areas. The ratios were generally lower in areas with warm weather (mean 0.13, range 0.03- to 0.29 per 100 elderly), and higher in areas with cold temperature (mean: 0.33, range 0.06 to 0.74 per 100 elderly) (p for difference = 0.008). | |
|  | | |
| Discussion | | |
| Study findings, (Summarise key study findings and describe how they support the conclusions reached.) | - Overall, since the end of March, the instantaneous reproduction number (Rt) declined over time similarly in 30 largest metropolitan areas, and after April 15, Rts in almost all areas reached 1 or below. Since then, the Rts remained around 1 in most areas and there were also a few small and short rebounds in some regions, suggesting the epidemic was persistent in those areas. The main decline was concurrent with the implementation of aggressive interventions in the US, while the occurrence of rebounds in some southern areas was coincident with the relaxation of interventions. - These suggested stringent interventions were effective in halting the epidemic. However, there were large geographic variations in the Rt patterns, partly due to different levels of interventions, geographic altitudes, and partly might be due to temperature variations. | |
| limitations | - Inability to account for the diverse detection capacities across regions. In regions with lower detection capacity, not only were there fewer cases detected (especially missing those with no or mild symptoms), but also the eligibilities for detection were more stringent. - The authors assumed a universal distribution of serial interval for all regions and over the whole time period. Serial interval may change due to interventions, regional characteristics, and the stage of epidemic. - The authors were not able to rigorously evaluate the virulence of SARS -CoV- 2. Although the authors briefly compared death rates across regions, due to large and unknown delays between virus infection and deaths in the US, most deaths would be diagnosed several weeks before. There was also a delay in death certifications. Additionally, most died cases were elderly people or those with existing chronic conditions. Therefore, assessing the virulence should untangle the confounding effects by health care resource capacities, evolving treatments, and patient's characteristics. - Secondary data analysis based on existing aggregated data suffer many types of unmeasured confounding. | |
| generalisability | - | |
| current knowledge (how the findings fit with current knowledge.) | - Interventions were effective in halting the epidemic. Without effective interventions, the peak of epidemic will reach higher and the epidemic process will last longer. Thus, the reproduction numbers will not decline until a large proportion of susceptible people are infected. - For example, during the week of March 25, Rts in Houston, Miami, and Orlando were relatively stable. After the end of March, due to national efforts in mitigating the epidemic, all Rt curves started declining. The state of Florida, however, did not officially issue the stay-at-home rule until April 3, 2020, where the curves already declined significantly. This posed some difficulties in assessing the intervention effects precisely. - On the other hand, a few cities demonstrated some significant impact of interventions on mitigating the epidemic. For example, some upper midwestern cities (e.g., Minneapolis-St. Paul and Milwaukee) implemented interventions earlier, ad lower peak Rts, and their Rts started declining early, while some small but possible negative association between temperature and virus transmissibility. However, there were large variations in the peak Rts among regions with lower temperature, partly due to different intervention effects and also might be due to cultural and social differences. It is also likely that other environmental factors such as living conditions may affect virus transmission. | |
| changes in diagnostic tests and access and any adjustment | There were large variations in case ascertainment criteria and availability of detection kits among different regions. | |

1. **Reduction of COVID-19 Incidence and Nonpharmacologic Interventions: Analysis Using a US County-Level Policy Data Set(Ebrahim & Ashworth, 2020)**

| Data Collection Item | Item description / sub-groups | |
| --- | --- | --- |
| Bibiliographic Information | | |
| Author | Ebrahim et al. | |
| Title | Reduction of COVID-19 Incidence and Nonpharmacologic Interventions: Analysis Using a US County-Level Policy Data Set | |
| Year of publication | 2020 | |
| Country | USA | |
|  | | |
| Methods | | |
| Type of Study | Analysing time-series data | |
| Time period and setting of the study | 1320 US counties; from March 1 to July 20, 2020, | |
| Intervention | Nonpharmacologic Interventions | |
| description of intervention | - 7 distinct nonpharmacologic Interventions (NPIs) policies, including the most widely deployed and accepted NPIs. (1) closure of nonessential workplaces, (2) shelter-in-place/stay-at-home orders, (3) enforcement of shelter-in-place/stay-at-home orders, (4) size restrictions on public gatherings, (5) school closures, (6) public transit closures, and (7) publicly available testing. - Nonessential workplace closures and shelter-in-place/stay-at-home orders was the target of observation in this study. | |
| setting of the intervention | 1320 counties from all 50 states in the United States | |
| Aim/ Objectives | To create a granular NPI data set at the county level and then analyze the relationship between NPI policies and changes in reported COVID-19 cases. | |
| Participant characteristics | Participants | - The full county NPI data set, hereon referred to as the “all policies” data set, yielded 2704 observations of NPI policies in 1320 counties from all 50 states in the United States. - The authors analyzed the all policies data set (n=2704) as well as the consolidated current version of the data set, hereon referred to as the “current policies” data set (n=1320), containing only the most recent timestamp for county NPIs. |
|  | Comparator details |  |
| Outcome measures | Case growth rates |  |
| Source of data | - A combination of county-level NPI data for 1320 US counties with multiple data sources, including daily county COVID-19 cases and deaths sourced from The New York Times. - Political demographics at the county level were drawn from the Kaiser Family Foundation and the MIT Election Data and Science Lab. | |
| Data analyiss | - Time-series correlational analyses were conducted combining the county-level NPI data for 1320 US counties. - Correlations were assessed with COVID-19 effective Rt estimates from the RT Live project, and political demographics at the county level. - T tests and chi-square tests with a Bonferroni correction applied for multiple hypothesis testing. - All analyses were conducted in Python notebooks that are available open source for review and global use under the Apache 2.0 license. | |
|  | | |
| Results | | |
| Study parameters | Weekly case growth rates;  NPI policy in each county;  All observations within the last 24 hours were pooled;  The mean was calculated for each binary | |
| outcomes (Mean values for the main categories of estimated outcomes of interest, as well as mean differences between the comparator groups.) | - Weekly case growth rates decreased over the 14 days following a workplace NPI by larger magnitudes for counties that had such nonessential workplace closure policies as compared to those that had openings (P=.004). - Decreasing case propagation following workplace closure was similarly observed in state-level estimates of Rt 14 days after the workplace policy (from RT Live) (P<.001). The effect for Rt was recapitulated at 28 days after the workplace policy (P<.001). These effects are statistically significant at our Bonferroni corrected α level of .0071. - To assess whether counties that had free public testing before a workplace policy observation were more likely to have had a workplace closure for that observation, counties were grouped based on their timelines into the categories of no testing, testing after workplace closure, and testing before workplace closure. A chi-square test for significance found no significant difference in workplace openings versus workplace closures for these categories (P=.08). - There were weak correlations between the political parties of local leadership and electorate and the policy of shelter-in-place enforcement. In the all policies data set, shelter-in-place enforcement was weakly correlated with Democratic party State House leadership (R=0.22) and Democratic voting proportion in the 2016 presidential election (R=0.21); all other correlations had an absolute value of R<0.2. - No correlations greater than 0.2 were observed between county political parties and the dates at which a positive intervention was initially made for a particular NPI policy. | |
| Characterising heterogeneity | As a consistent trend, for both heavily populated urban counties like Los Angeles County (10 million  residents) and sparsely populated rural counties like Siskiyou County (44,000 residents), the end of a workplace closure appears to precede an increase in cases by 2-6 weeks. | |
|  | | |
| Discussion | | |
| Study findings, (Summarise key study findings and describe how they support the conclusions reached.) | - The analysis revealed a wide variation in county-level policies both within and among states. - The authors identified a correlation between workplace closures and lower growth rates of COVID-19 cases. - They also found weak correlations between shelter-in-place enforcement and measures of Democratic local voter proportion and elected leadership. | |
| limitations | - Firstly, the data collection, while rigorous, was affected by a number of factors both inherent to the study and external that could skew outcomes. - Secondly, the Hikma Health data set comprises 2 timepoints for 2 of the 7 NPI policies originally assessed. The analyses are subject to the caveat that changes in the other 5 unrecorded NPI policies may theoretically confound any associations. Of these NPI policies, enforcement of shelter-in-place/stay-at-home orders, school closures, and publicly available testing were unlikely to change before August 2020, whereas size restrictions on public gatherings and public transit closures may have been reversed and therefore might be more significant confounds. - Finally, the analysis identifies correlations in the data set without any implication of causality. In order to establish causation, NPI policies would have to be implemented as a coordinated randomized controlled trial across counties, which is unlikely. | |
| generalisability | The outcomes were associated with when public testing started in each county and the rate at which counties were able to test. | |
| current knowledge (how the findings fit with current knowledge.) | - The authors found that across the United States, there was significant variability in NPI policy implementation among counties, both within and among states. NPI policies themselves for any given county are largely uncorrelated, with the singular exception of workplace closure and shelter-in-place NPIs. This finding supports previous research that has also found wide variation in policies across states, which was associated with statistically significant differences in rates of COVID-19 transmission. - For both heavily populated urban counties like Los Angeles County (10 million residents) and sparsely populated rural counties like Siskiyou County (44,000 residents), the end of a workplace closure appears to precede an increase in cases by 2-6 weeks. This heatmap timeline could be confounded by when public testing started in each county and the rate at which counties were able to test. - Across all counties, those with work closures had significantly lower rates of subsequent COVID-19 case growth compared to counties that did not. However, these results do not in any way demonstrate causation but rather a temporally informative correlation between NPI policy and COVID-19 rates. - Current research on political affiliation and attitudes toward COVID-19 policies has shown that differences between Democrats and Republicans are more significant than differences across race or gender. Reports from the Pew Research Center have shown that Democrats are more likely to see COVID-19 as a serious threat. | |
| changes in diagnostic tests and access and any adjustment | - To assess whether counties that had free public testing before a workplace policy observation were more likely to have had a workplace closure for that observation, counties were grouped based on their timelines into the categories of no testing, testing after workplace closure, and testing before workplace closure. - While the authors have recorded the date at which public testing became available in each county, the rate of testing by county remains generally unknown. | |

1. **Effects of social distancing on the spreading of COVID-19 inferred from mobile phone data(Khataee et al., 2021)**

| Data Collection Item | Item description / sub-groups | |
| --- | --- | --- |
| Bibiliographic Information | | |
| Author | Khataee et al. | |
| Title | Effects of social distancing on the spreading of COVID-19 inferred from mobile phone data | |
| Year of publication | 2021 | |
| Country | Italy, Spain, France, UK, Germany, Switzerland, Netherlands, Belgium and Sweden | |
|  | | |
| Methods | | |
| Type of Study |  | |
| Time period and setting of the study | Nine selected European countries (Italy, Spain, France, UK, Germany, Switzerland, Netherlands, Belgium and Sweden.); over 90 days starting from 13-January-2020   - Time 0 corresponds to the day when a country first reported ­ 5 daily deaths. - Daily volume of requests made to Apple Maps for directions by transportation type per country compared to a baseline volume on 13-January-2020. | |
| Intervention | Social distancing and lockdown | |
| description of intervention | - Varied by countries | |
| setting of the intervention | Community of nine selected European countries (Italy, Spain, France, UK, Germany, Switzerland, Netherlands, Belgium and Sweden.) | |
| Aim/ Objectives | To analysed the interdependence of epidemic and mobility data and identified a quantitative relation between parameters of social distancing and key characteristics of the COVID-19 pandemic. | |
| Participant characteristics | Participants | - 60,385,788 people (Italy); 46,754,778 people (Spain); 65,273,511 people (France); 67,886,011 people (UK); 83,783,942 people (Germany); 8,654,622 people (Switzerland); 17,134,872 people (Netherlands); 11,492,641 people (Belgium); 10,099,265 people (Sweden) |
|  | Comparator details |  |
| Outcome measures | basic reproductive  Number (R0) | i.e. number of new secondary infections caused by a single infected in a fully susceptible population |
|  | Number of daily deaths |  |
| Source of data | - To characterise social distancing responses, mobile phone mobility trend data from Apple Inc was used | |
| Data analyiss | - Pearson correlation coefficients was used to test the relashionships. | |
|  | | |
| Results | | |
| Study parameters | Number of daily deaths per million (1M) population;  Mitigation efforts are estimated from mobile phone tracking data (A day is defined as midnight-to-midnight, US Pacific time). | |
| outcomes (Mean values for the main categories of estimated outcomes of interest, as well as mean differences between the comparator groups.) | - The time to the peak from the official national lock-down tc − tNL varies in a range from 10 days in Italy to more than 3 weeks in case of Switzerland, and the time from the change in mobility to the peak, tc − teff , ranges from 19 days (Italy and Spain) up to 34 days (Sweden). - Neither the time of the peak tc nor the parameters characterising the time and strength of social distancing teff, tNL , μ correlates—as a single parameter—well with the total number of deaths. - The results indicate a strong positive correlation between the drop in basic reproductive number, R01 − R02 and the restriction of mobility μ. - The time elapsed between the peak and the social distancing, tc − teff , correlates negatively with the severity of the mobility restrictions. | |
| Characterising heterogeneity | - The choice of countries was motivated by the requirements that each of these countries (i) had a relatively large disease-associated death toll (i.e. typically above 10/day and more than 2000 overall) so we can assume that the deterministic component of the epidemic dynamics dominates over random fluctuations. (ii) The selected countries spent a suitably long time in the decaying phase of the epidemic, thus allowing its precise characterisation. - Statistical data on COVID-19-related deaths are considered to be more robust than that of daily cases of new infections. The latter is affected by the number of tests performed as well as by the testing strategy – e.g. its restriction to symptomatic patients – which may be highly variable across countries and often changes during the course of the epidemic. The time course of daily deaths can be considered as a more reliable indirect delayed indicator of daily infections. The authors thus did not address apparent differences in the case fatality ratio, and restrict our focus to the recorded COVID-19-associated death toll. | |
|  | | |
| Discussion | | |
| Study findings, (Summarise key study findings and describe how they support the conclusions reached.) | - Our analysis thus indicates that social distancing has two effects: it reduces the basic reproduction number of the infection as expected, and shortens the time required for the epidemic to peak. | |
| limitations | - Although the mobility data may have bias in the mobility signal, it may have relatively little direct effect on data reported for the countries studied here. | |
| generalisability |  | |
| current knowledge (how the findings fit with current knowledge.) | - | |
| changes in diagnostic tests and access and any adjustment | Each country had different testing stratifies and testing access. | |

1. **Impact of lockdown on COVID-19 prevalence and mortality during 2020 pandemic: observational analysis of 27 countries(Meo et al., 2020)**

| Data Collection Item | Item description / sub-groups | |
| --- | --- | --- |
| Bibiliographic Information | | |
| Author | Meo et al. | |
| Title | Impact of lockdown on COVID-19 prevalence and mortality during 2020 pandemic: observational analysis of 27 countries | |
| Year of publication |  | |
| Country | Algeria; Argentina; Australia ; Bahrain ; Belgium ; Chile ; China ; Colombia ; Cuba ; Djibouti ; Egypt ; France ; Germany ; Ghana ; India ; Italy ; Nepal ; Nigeria ; Pakistan ; Philippines ; Republic of Congo ; Russian Federation ; Saudi Arabia ; South Africa ; Spain ; The United Kingdom ; United States of America. | |
|  | | |
| Methods | | |
| Type of Study | Observational study | |
| Time period and setting of the study | Twenty-seven countries were randomly selected from the different continents; 12/01/2020/to 19/04/2020   - 15 days before, 15 days during and 15 days after lockdown. | |
| Intervention | Lockdown | |
| description of intervention | - Date of lockdown in each country: March 24, 2020 Algeria ; March 20, 2020 Argentina ; March 23, 2020 Australia ; February 25, 2020 Bahrain ; March 12, 2020 Belgium ; March 18, 2020 Chile ; January 23, 2020 China ; March 24, 2020 Colombia ; April 1, 2020 Cuba ; March 15, 2020 Djibouti ; March 25, 2020 Egypt ; March 17, 2020 France ; March 23, 2020 Germany ; March 30, 2020 Ghana ; March 25, 2020 India ; March 9, 2020 Italy ; March 24, 2020 Nepal ; March 30, 2020 Nigeria ; April 1, 2020 Pakistan ; March 15, 2020 Philippines ; March 24, 2020 Republic of Congo ; March 31, 2020 Russian Federation ; March 25, 2020 Saudi Arabia ; March 26, 2020 South Africa ; March 14, 2020 Spain ; March 23, 2020 The United Kingdom ; March 17, 2020 United States of America. | |
| setting of the intervention | Community of the countries | |
| Aim/ Objectives | To assess the impact of 15 days before, 15 days during, and 15 days after the lockdown on the trends in the prevalence and mortality in 27 countries during COVID-19 pandemic. | |
| Participant characteristics | Participants | Population of Twenty-seven selected countries. |
|  | Comparator details |  |
| Outcome measures | mean number of deaths |  |
|  |  |  |
| Source of data | - The daily reports on COVID-19 published by the World Health Organization were carefully reviewed and data were collected. - The lockdown data were obtained from concerned countries and their ministries however, populations of the countries were obtained from the world bank. | |
| Data analyiss | - Correlation analysis. | |
|  | | |
| Results | | |
| Study parameters | Mean number of deaths 15 days before, 15 days during and 15 days after the lockdown in 27 countries; | |
| outcomes (Mean values for the main categories of estimated outcomes of interest, as well as mean differences between the comparator groups.) | - The findings showed that 15 days after the lockdown there was a trend toward a decline, but no significant decline in the mean prevalence and mean mortality rate due to the COVID-19 pandemic compared to 15 days before, and 15 days during the lockdown in 27 countries. - The mean growth factor for number of cases was 1.18 and for mortality rate was 1.16. | |
| Characterising heterogeneity |  | |
|  | | |
| Discussion | | |
| Study findings, (Summarise key study findings and describe how they support the conclusions reached.) | - The findings indicate that 15 days after the lockdown, daily cases of COVID-19 and the growth factor of the disease showed a declined trend, but there was no significant decline in the prevalence and mortality. | |
| limitations | - The authors were unable to investigate confounding factors including how much people vary in: (1) adherence to lockdown, (2) adoption of protocols of social distancing, (3) practice of health hygienic conditions and (4) experience disease testing systems of their individual countries. | |
| generalisability |  | |
| current knowledge (how the findings fit with current knowledge.) | - The study has shown evidence that lockdown measures are consistently beneficial, with quarantining of people who were exposed to confirmed or suspected cases preventing 44% to 81% of new cases and 31% to 63% deaths, compared to a lack of any lockdown measure. The authors showed that a lockdown may prove helpful in controlling the COVID-19 outbreak. - We found that daily cases of COVID-19 and growth factor results show a declined trends 15 days after the lockdown period. The present study findings did not support the hypothesis that lockdown will significantly decrease the number of cases. | |
| changes in diagnostic tests and access and any adjustment | - | |

1. **Effectiveness of contact tracing and quarantine on reducing COVID-19 transmission: a retrospective cohort study** (Malheiro et al., 2020)

| Data Collection Item | Item description / sub-groups | |
| --- | --- | --- |
| Bibiliographic Information | | |
| Author | Malheiro et al. | |
| Title | Effectiveness of contact tracing and quarantine on reducing COVID-19 transmission: a retrospective cohort study | |
| Year of publication | 2020 | |
| Country | Portugal | |
|  | | |
| Methods | | |
| Type of Study | Retrospective cohort study | |
| Time period and setting of the study | Eastern Porto; from March 1st, 2020, and April 30th, 2020.   - Since the incubation period for COVID-19 is, on average, 5-6 days, we decided to set a lag of 7 days from the Decree’s execution date, by selecting March 29th as the cutoff point after which the effects of lockdown measures may have become apparent. | |
| Intervention | Contact tracing and quarantine of close contacts | |
| description of intervention | - Contact tracing was defined as the systematic identification, through a detailed 94 interview with the COVID-19 patient or their caregiver, of all household, family, work/school and social contacts who have had contact with a confirmed case, from 2 days prior to symptom onset of   the case and up to 14 days afterwards. Identified contacts were then classified into close and casual contacts and informed of their exposure. Close contacts (high-risk) were defined as individuals who have spent 15 minutes or more in close proximity (2 meters or less) to, or in 100 a closed space with, a case.   - All close contacts were placed under mandatory quarantine, and assessed once daily, by 102 telephone, for potential symptoms of COVID-19. Follow-up of close contacts ended 14 days 103 after the last exposure, or if the contact was, in the meantime, diagnosed with COVID-19. 104 Work-related contacts of healthcare and nursing home staff were excluded, as they were 105 identified and managed following different guidelines. Close contacts with fever (temperature 106 ≥38°C), cough, dyspnea, and/or other mild symptoms were transferred directly to a 107 healthcare facility for further evaluation and testing. | |
| setting of the intervention |  | |
| Aim/ Objectives | To assess the effectiveness of those measures on reducing transmission of SARS-CoV-2 in a community setting. | |
| Participant characteristics | Participants | N= 98 (17.8%) cases  The intervention group comprised all COVID-19 confirmed cases that were either identified as close contacts of an index case, or returned from affected areas and placed under mandatory quarantine, with daily follow-up until laboratory confirmation of SARS-CoV-2 infection. |
|  | Comparator details | N=453 (82.2%) cases  The control group included all COVID-19 confirmed cases that were not subject to contact tracing nor to quarantine measures preceding the diagnosis. |
| Outcome measures | Attack rates | Attack rates were calculated by dividing the number of secondary cases by the number of close contacts. |
|  |  |  |
| Source of data | - Data source was the notification database of SINAVE, and protected databases used for the 121 daily monitoring registration of close contacts follow-up. | |
| Data analyiss | - Chi-squared and Mann-Whitney-U tests were used to evaluate the distributions of categorical and continuous variables, respectively, between the intervention and control groups. - Analyses were performed using Microsoft Excel 2016 and R, version 4.0.0. | |
|  | | |
| Results | | |
| Study parameters |  | |
| outcomes (Mean values for the main categories of estimated outcomes of interest, as well as mean differences between the comparator groups.) | - Cases under mandatory quarantine and daily follow-up at the time of diagnosis showed a 163 significantly lower median number of close contacts (median: 2, IQR 1-4) when compared to 164 the control group (median: 0, IQR 0-2, p-value<0.001). - The median number of secondary cases by index case was lower for the cases reported under the State of Emergency at a 7-day lag (median: 0, IQR 0-0, in both periods, p-value=0.039), with no difference between cohorts. - The intervention group had a shorter time between symptom onset and specimen collection (median: 3 days, IQR 1-6, vs. median: 5 days, IQR 2-7, p-value=0.004) and fewer close contacts (median: 0, IQR 0-2, vs. 23 median: 2, IQR 1-4, p-value<0.001). | |
| Characterising heterogeneity | - | |
|  | | |
| Discussion | | |
| Study findings, (Summarise key study findings and describe how they support the conclusions reached.) | - In the present study, contact tracing and quarantine of close contacts appear to have no role in reducing the number of secondary cases of COVID-19. | |
| limitations | - Contact identification is highly dependent on the information provided by COVID-19 cases or their caregivers. - Travelers from affected countries were often unknown to local authorities prior to their diagnosis, thus limiting the ability to block the transmission chain in this population. - Finally, it is possible other national measures with a lesser impact may have contributed to some residual confounding. | |
| generalisability | - | |
| current knowledge (how the findings fit with current knowledge.) | - Local public health measures are effective at reducing both the time between symptom onset and laboratory diagnosis and the median number of close contacts per case. No effect was apparent on secondary cases figures, suggesting that further measures may be required to break the transmission chain of COVID-19. Nevertheless, national restriction measures appear to have an impact on reducing transmission of SARS-CoV-2. | |
| changes in diagnostic tests and access and any adjustment | - | |
