# Supplementary material for: Effectiveness of non-pharmaceutical public health interventions against COVID-19: A systematic review and meta-analysis
Source: PLoS One. 2021 Nov 23;16(11):e0260371. doi: 10.1371/journal.pone.0260371 (PMC8610259; doi:10.1371/journal.pone.0260371)
Supplement: S4 Table — (DOCX) [file pone.0260371.s008.docx]

**S4 Table: Results of the EPOK risk of bias assessment for interrupted time series studies**

|  | **Studies** | **Intervention independent of other changes?** | **Shape of the intervention effect pre-specified?** | **Intervention unlikely to affect data collection?** | **Knowledge of the allocated interventions?** | **Incomplete outcome data adequately?** | **Selective outcome reporting?** | **Other risks of bias?** | **Final score** |
| --- | --- | --- | --- | --- | --- | --- | --- | --- | --- |
| 1 | (Adly et al., 2020) |  |  |  |  |  |  |  | 2 out of 7 |
| 2 | (Alimohamadi et al., 2020) |  |  |  |  |  |  |  | 5 out of 7 |
| 3 | ([Auger et al., 2020](#_ENREF_3)) |  |  |  |  |  |  |  | 5 out of 7 |
| 4 | ([Camila Alves dos Santos et al., 2020](#_ENREF_4)) |  |  |  |  |  |  |  | 5 out of 7 |
| 5 | ([Cobb et al., 2020](#_ENREF_6)) |  |  |  |  |  |  |  | 3 out of 7 |
| 6 | ([Courtemanche et al., 2020](#_ENREF_7)) |  |  |  |  |  |  |  | 6 out of 7 |
| 7 | ([Ji et al., 2020](#_ENREF_10)) |  |  |  |  |  |  |  | 5 out of 7 |
| 8 | ([Kraemer et al., 2020](#_ENREF_12)) |  |  |  |  |  |  |  | 5 out of 7 |
| 9 | ([Lee et al., 2020](#_ENREF_13)) |  |  |  |  |  |  |  | 5 out of 7 |
| 10 | ([Liang et al., 2020](#_ENREF_14)) |  |  |  |  |  |  |  | 5 out of 7 |
| 11 | ([McGrail et al., 2020](#_ENREF_17)) |  |  |  |  |  |  |  | 6 out of 7 |
| 12 | ([Mitra et al., 2020](#_ENREF_19)) |  |  |  |  |  |  |  | 4 out of 7 |
| 13 | ([Padalabalanarayanan et al., 2020](#_ENREF_20)) |  |  |  |  |  |  |  | 2 out of 7 |
| 14 | ([Pan et al., 2020](#_ENREF_21)) |  |  |  |  |  |  |  | 4 out of 7 |
| 15 | ([Patel et al., 2020](#_ENREF_22)) |  |  |  |  |  |  |  | 4 out of 7 |
| 16 | ([Rubin et al., 2020](#_ENREF_24)) |  |  |  |  |  |  |  | 5 out of 7 |
| 17 | ([Saez et al., 2020](#_ENREF_25)) |  |  |  |  |  |  |  | 5 out of 7 |
| 18 | ([Siedner et al., 2020](#_ENREF_29)) |  |  |  |  |  |  |  | 4 out of 7 |
| 19 | ([Silva et al., 2020](#_ENREF_30)) |  |  |  |  |  |  |  | 4 out of 7 |
| 20 | ([Tobias, 2020](#_ENREF_33)) |  |  |  |  |  |  |  | 5 out of 7 |
| 21 | ([Xu et al., 2020](#_ENREF_34)) |  |  |  |  |  |  |  | 5 out of 7 |
| 22 | ([Cruz, 2020](#_ENREF_8)) |  |  |  |  |  |  |  | 5 out of 7 |
| 23 | ([Castillo et al., 2020](#_ENREF_5)) |  |  |  |  |  |  |  | 3 out of 7 |
| 24 | ([Sen et al., 2020](#_ENREF_27)) |  |  |  |  |  |  |  | 6 out of 7 |
| 25 | ([Salvatore et al., 2020](#_ENREF_26)) |  |  |  |  |  |  |  | 5 out of 7 |
| 26 | ([Pillai et al., 2020](#_ENREF_23)) |  |  |  |  |  |  |  | 6 out of 7 |
| 27 | ([Shi et al., 2021](#_ENREF_28)) |  |  |  |  |  |  |  | 5 out of 7 |
| 28 | ([Supino et al., 2020](#_ENREF_32)) |  |  |  |  |  |  |  | 6 out of 7 |
| 29 | ([Soodejani et al., 2020](#_ENREF_31)) |  |  |  |  |  |  |  | 4 out of 7 |
| 30 | ([Yu, 2020](#_ENREF_35)) |  |  |  |  |  |  |  | 4 out of 7 |
| 31 | ([Ebrahim et al., 2020](#_ENREF_9)) |  |  |  |  |  |  |  | 4 out of 7 |
| 32 | ([Khataee et al., 2021](#_ENREF_11)) |  |  |  |  |  |  |  | 6 out of 7 |
| 33 | ([Meo et al., 2020](#_ENREF_18)) |  |  |  |  |  |  |  | 5 out of 7 |
| High-risk; Low-risk; Uncear-risk | | | | | | | | | |
