# Supplementary material for: Effectiveness of non-pharmaceutical public health interventions against COVID-19: A systematic review and meta-analysis
Source: PLoS One. 2021 Nov 23;16(11):e0260371. doi: 10.1371/journal.pone.0260371 (PMC8610259; doi:10.1371/journal.pone.0260371)
Supplement: S5 Table — (DOCX) [file pone.0260371.s009.docx]

**S5 Table: results of the EPOK risk of bias assessment for studies with a separate control group**

|  | Stiudies | **Random sequence generation?** | **Allocation concealment?** | **Baseline outcome measurements similar?** | **Baseline characteristics similar?** | **Incomplete outcome data?** | **Knowledge of the allocated interventions?** | **Protection against contamination?** | **Selective outcome reporting?** | **and other risks of bias** | **Final score** |
| --- | --- | --- | --- | --- | --- | --- | --- | --- | --- | --- | --- |
| 1 | ([Lyu et al., 2020](#_ENREF_15)) |  |  |  |  |  |  |  |  |  | 6 out of 9 |
| 2 | ([Malheiro et al., 2020](#_ENREF_16)) |  |  |  |  |  |  |  |  |  | 5 out of 9 |
| High-risk; Low-risk; Uncear-risk | | | | | | | | | | | |
